# Retinal ganglion cell repopulation for vision restoration in optic neuropathy: a roadmap from the RReSTORe Consortium

**DOI:** 10.1186/s13024-023-00655-y

**Published:** 2023-09-21

**Authors:** Jonathan R. Soucy, Erika A. Aguzzi, Julie Cho, Michael James Gilhooley, Casey Keuthan, Ziming Luo, Aboozar Monavarfeshani, Meher A. Saleem, Xue-Wei Wang, Juilette Wohlschlegel, The RReSTORe Consortium, Jonathan R. Soucy, Erika A. Aguzzi, Julie Cho, Michael James Gilhooley, Casey Keuthan, Ziming Luo, Aboozar Monavarfeshani, Meher A. Saleem, Xue-Wei Wang, Juilette Wohlschlegel, Abdelrahman Y. Fouda, Ajay Ashok, Ala Moshiri, Alain Chedotal, Amberlynn A. Reed, Amjad Askary, An-Jey A. Su, Anna La Torre, Archana Jalligampala, Ariadna Silva-Lepe, Arupratan Das, Barbara Wirostko, Benjamin J. Frankfort, Benjamin Sivyer, Bhagwat Alapure, Brent Young, Brian Clark, Bryan William Jones, Chase Hellmer, Claire Mitchell, Claire Ufongene, Dan Goldman, David Feldheim, David H. Gutmann, David J. Calkins, David Krizaj, David M. Gamm, Diana C. Lozano, Diane E. Bovenkamp, Dong Feng Chen, Elena Vecino, Ephraim F. Trakhtenberg, Feng Tian, Fengquan Zhou, Gillian J. McLellan, Harry A. Quigley, Hashem Abu Serhan, James R. Tribble, Jason Meyer, Jeff Gross, Jeff S. Mumm, Jeremy M. Sivak, Jingliang Simon Zhang, Jiun L. Do, Jonathan Crowston, Julie Chen, Juliette McGregor, Kalyan C. Vinnakota, Kang-Chieh Huang, Karen Peynshaert, Katherine E. Uyhazi, Keith Martin, Ken Muller, Kevin K. Park, Kin-Sang Cho, Kun-Che Chang, Larry Benowitz, Leonard A. Levin, Levi Todd, Lies De Groef, Lieve Moons, Luis Alarcon-Martinez, Mandeep S. Singh, Manuel Vidal-Sanz, Mariana S. Silveira, Marina Pavlou, Matthew B. Veldman, Matthew Van Hook, Melanie Samuel, Mengming Hu, Micalla Peng, Michael Young, Michel Cayouette, Mohammad H. Geranmayeh, Mollie Woodworth, Monica Vetter, Nicholas R. Marsh-Armstrong, Pete A. Williams, Pratheepa Kumari Rasiah, Preeti Subramanian, Qi N. Cui, Rebecca M. Sappington, Reem Amine, Richard Eva, Robert J. Johnston, Roman J. Giger, Ross Ethier, Sadaf Abed, Sehrish Nizar Ali Momin, Seth Blackshaw, Shane A. Liddelow, Stella Mary, Stephen Atolagbe, Supraja Varadarajan, Tareq I. Nabhan, Tasneem Khatib, Tasneem Putliwala Sharma, Thomas Brunner, Tom Greenwell, Tonia S. Rex, Trent Watkins, Tudor C. Badea, V. Vrathasha, Venkata Ramana Murthy Chavali, Viviane M. Oliveira-Valença, Wai Lydia Tai, Wyndham M. Batchelor, Xian-Jie Yang, Yong Park, Yuan Pan, Petr Baranov, Adriana Di Polo, Brad Fortune, Kimberly K. Gokoffski, Jeffrey L. Goldberg, William Guido, Alex L. Kolodkin, Carol A. Mason, Yvonne Ou, Thomas A. Reh, Ahmara G. Ross, Brian C. Samuels, Derek Welsbie, Donald J. Zack, Thomas V. Johnson, Petr Baranov, Adriana Di Polo, Brad Fortune, Kimberly K. Gokoffski, Jeffrey L. Goldberg, William Guido, Alex L. Kolodkin, Carol A. Mason, Yvonne Ou, Thomas A. Reh, Ahmara G. Ross, Brian C. Samuels, Derek Welsbie, Donald J. Zack, Thomas V. Johnson

**Affiliations:** 1 Department of Ophthalmology, Schepens Eye Research Institute of Mass. Eye and Ear, Harvard Medical School, Boston, MA USA; 2 The Institute of Ophthalmology, University College London, London, England, UK; 3 Spencer Center for Vision Research, Byers Eye Institute, Stanford University School of Medicine, Palo Alto, CA USA; 4 Moorfields Eye Hospital, London, England, UK; 5 Department of Ophthalmology, Wilmer Eye Institute, Johns Hopkins University School of Medicine, Baltimore, MD USA; 6 Center for Brain Science and Department of Molecular and Cellular Biology, Harvard University, Cambridge, MA USA; 7 Kirby Neurobiology Center, Boston Children’s Hospital, Boston, MA USA; 8 Bascom Palmer Eye Institute, University of Miami Health System, Miami, FL USA; 9 Department of Orthopaedic Surgery, The Johns Hopkins University School of Medicine, Baltimore, MD USA; 10 Department of Biological Structure, University of Washington, Seattle, WA USA; 11 Department of Neuroscience, University of Montreal, Montreal, QC Canada; 12 University of Montreal Hospital Research Centre, Montreal, QC Canada; 13 Discoveries in Sight Research Laboratories, Devers Eye Institute and Legacy Research Institute, Legacy Health, Portland, OR USA; 14 Department of Ophthalmology, Roski Eye Institute, University of Southern California, Los Angeles, CA USA; 15 Department of Anatomical Sciences and Neurobiology, School of Medicine, University of Louisville, Louisville, KY USA; 16 The Solomon H Snyder, Department of Neuroscience, Johns Hopkins University School of Medicine, Baltimore, MD USA; 17 Departments of Pathology and Cell Biology, Neuroscience, and Ophthalmology, College of Physicians and Surgeons, Zuckerman Mind Brain Behavior Institute, Columbia University, New York, NY USA; 18 Department of Ophthalmology, University of California, San Francisco, CA USA; 19 Departments of Ophthalmology and Neurology, University of Pennsylvania, Philadelphia, PA USA; 20 Department of Ophthalmology and Visual Sciences, Callahan Eye Hospital, University of Alabama at Birmingham, Birmingham, AL USA; 21 Shiley Eye Institute and Viterbi Family Department of Ophthalmology, University of California, San Diego, CA USA; 22 Glaucoma Center of Excellence, Wilmer Eye Institute, Johns Hopkins University School of Medicine, Baltimore, 21287 MD USA; 23 Departments of Neuroscience, Molecular Biology & Genetics, and Genetic Medicine, Johns Hopkins University School of Medicine, Baltimore, MD USA; 24 Cellular & Molecular Medicine Program, Johns Hopkins University School of Medicine, Baltimore, 21287 MD USA

**Keywords:** Retinal ganglion cells, Transplantation, Neuroprotection, Organoids, Stem cells, Regenerative medicine, Ophthalmology, Glaucoma, Optic neuropathy

## Abstract

Retinal ganglion cell (RGC) death in glaucoma and other optic neuropathies results in irreversible vision loss due to the mammalian central nervous system’s limited regenerative capacity. RGC repopulation is a promising therapeutic approach to reverse vision loss from optic neuropathies if the newly introduced neurons can reestablish functional retinal and thalamic circuits. In theory, RGCs might be repopulated through the transplantation of stem cell-derived neurons or via the induction of endogenous transdifferentiation. The RGC Repopulation, Stem Cell Transplantation, and Optic Nerve Regeneration (RReSTORe) Consortium was established to address the challenges associated with the therapeutic repair of the visual pathway in optic neuropathy. In 2022, the RReSTORe Consortium initiated ongoing international collaborative discussions to advance the RGC repopulation field and has identified five critical areas of focus: (1) RGC development and differentiation, (2) Transplantation methods and models, (3) RGC survival, maturation, and host interactions, (4) Inner retinal wiring, and (5) Eye-to-brain connectivity. Here, we discuss the most pertinent questions and challenges that exist on the path to clinical translation and suggest experimental directions to propel this work going forward. Using these five subtopic discussion groups (SDGs) as a framework, we suggest multidisciplinary approaches to restore the diseased visual pathway by leveraging groundbreaking insights from developmental neuroscience, stem cell biology, molecular biology, optical imaging, animal models of optic neuropathy, immunology & immunotolerance, neuropathology & neuroprotection, materials science & biomedical engineering, and regenerative neuroscience. While significant hurdles remain, the RReSTORe Consortium’s efforts provide a comprehensive roadmap for advancing the RGC repopulation field and hold potential for transformative progress in restoring vision in patients suffering from optic neuropathies.

## Introduction

Retinal ganglion cell (RGC) dysfunction is the pathological feature of all optic neuropathies and, in the case of RGC death, results in irreversible vision loss [1, 2]. RGC axons transmit visual information from the eye to the brain, and progressive vision loss occurs when RGCs and their axons degenerate. Blindness caused by optic neuropathies is irreversible due to the limited regenerative capacity of the mammalian central nervous system (CNS) [3]. Therefore, developing innovative regenerative medicine approaches to restore vision loss to optic neuropathies would be transformative.

Ophthalmology is at the frontier of regenerative cell therapy as applied to chronic neurodegenerative diseases and has had several success stories. Transplantation of retinal pigment epithelium is under active investigation in patients as a part of an ongoing clinical trial, and transplantation of photoreceptors derived from human pluripotent stem cells is poised to begin phase I clinical trials soon [4]. These interventions are pioneering the replacement of dysfunctional retinal cells to restore vision and support the premise that RGC transplantation may be capable of reversing vision loss from optic neuropathies.

There are several reasons why the field of RGC repopulation is relatively immature compared to repopulation efforts for other types of retinal cells, but there is reason for optimism. RGCs are inherently more diverse than retinal pigment epithelium and retinal photoreceptors, comprising more than 40 subtypes in mice and more than 15 in primates [5–7]. Moreover, their wiring properties are more intricate than photoreceptors, receiving synaptic input from a variable number (up to dozens) of bipolar and amacrine cells and extending a long axon that must navigate through the optic nerve and optic chiasm into one of several subcortical visual nuclei in the brain [8]. Although RGC repopulation has long been considered [9], recent scientific advances (reviewed here) suggest that this audacious goal may be feasible [10, 11].

To help propel the field forward, the RGC Repopulation, Stem Cell Transplantation, and Optic Nerve Regeneration (RReSTORe) Consortium was established to bring together a diverse group of investigators (more than 200 worldwide) from complementary fields and with broad expertise (http://rrestore.info) [12]. From January through April 2022, the RReSTORe Consortium engaged in a virtual consensus-building process to identify the most pressing challenges and questions that need to be addressed to bring RGC repopulation towards clinical translation. On April 29^th^, 2022, consortium members met in Denver, Colorado, to engage in a daylong workshop designed to delineate these challenges, review the current state of the field, and brainstorm experimental frameworks to advance goals of vision restoration in optic neuropathies. Workshop discussions were held by five *subtopic discussion groups (SDGs)* running concurrently, which addressed a comprehensive set of goals that need to be obtained to restore functional vision in patients suffering from severe optic neuropathy. The SDGs included: (1) RGC Development and Differentiation, (2) Transplantation Methods and Models, (3) RGC Survival, Maturation, and Host Interactions, (4) Inner Retinal Wiring, and (5) Eye-to-Brain connectivity. Subsequently, the RReSTORe Consortium has continued to foster international collaboration through a series of discussions. Herein, pertinent aspects of those discussions are summarized, briefly recapitulating the state of the field and, more importantly, focusing on the most critical outstanding questions and obstacles that must be overcome within the next several years to enable the clinical translation of RGC replacement to prevent and reverse blindness. Notably, some of the obstacles/goals identified transcend RGC replacement. Focused work in these areas may also be applicable to neuroprotective or regenerative paradigms that seek to preserve or enhance function in optic neuropathies short of overt neuronal replacement.

## SDG #1. RGC development and differentiation

**Fig. 1:**
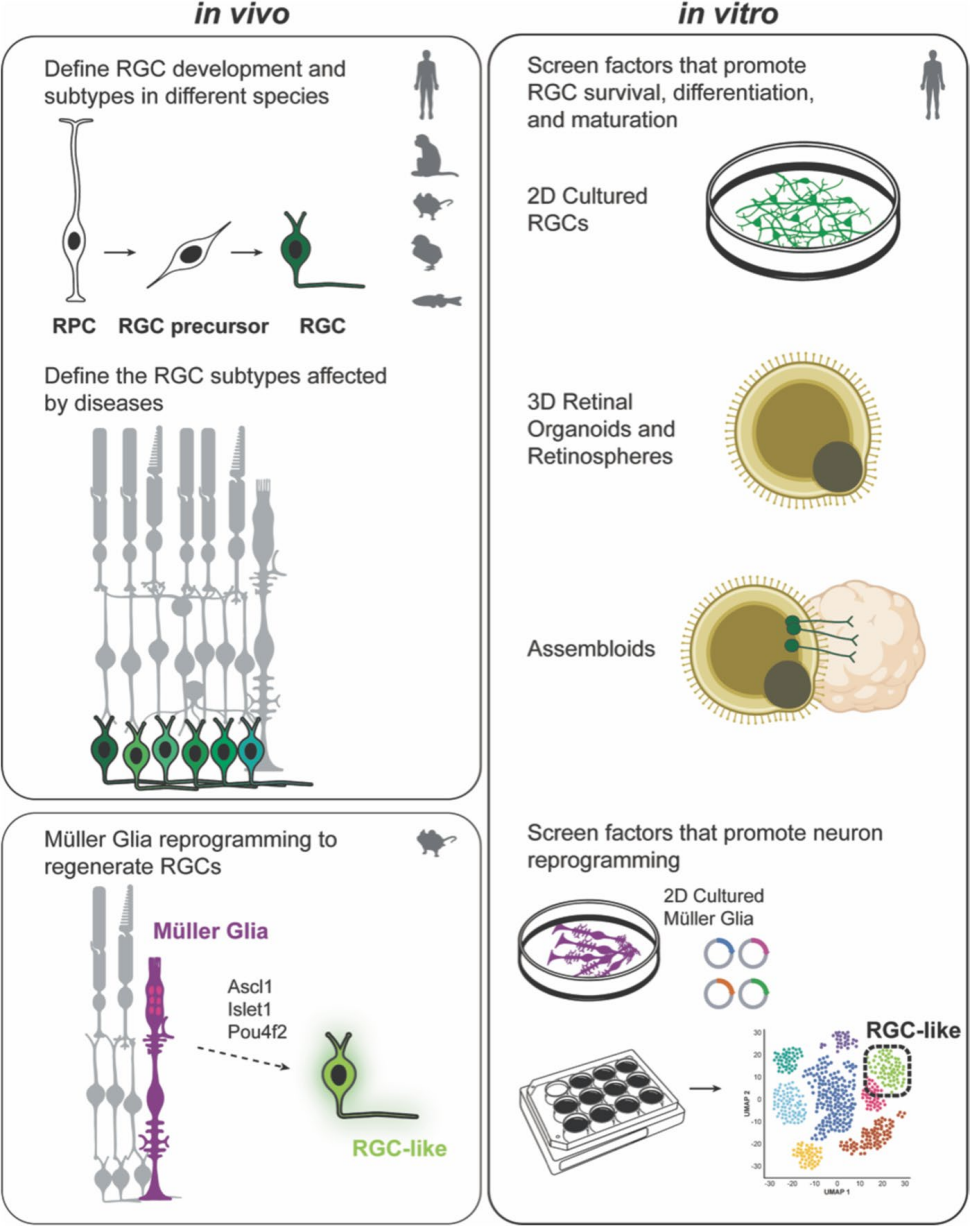
RGC development, subtype specification, differentiation, and regeneration. Retinal progenitor cells (RPC), RGC precursors, and mature RGCs can be defined and isolated from various species. Neuronal replacement therapies will be advanced by defining the RGC subtypes affected by various optic neuropathies and developing methods to target donor RGC maturation into specific subtypes. In vitro systems serve as a source of RGCs for cell replacement therapies and are useful for screening factors that promote RGC differentiation, maturation, and survival. RGCs can be cultured in monolayers, 3D retinal organoids, retinospheres, and assembloids. Through direct reprogramming in vivo and in vitro, Müller glia can also be a source for newborn mammalian RGCs, and factors that promote neuron reprogramming can be identified. While RGCs can be isolated from various species, RGC repopulation through reprogramming is currently only studied in mice, but in vitro studies can be performed using human samples

A fundamental challenge to functional RGC repopulation is the derivation of *bona fide* RGCs from de novo sources. Advances in stem cell biology have yielded multiple approaches for differentiating RGCs from pluripotent cells [13–15]. Indeed, developing techniques to reliably produce RGC-like cells from pluripotent stem cells at the scale needed for transplantation is a key achievement from the past decade and supports the current feasibility of RGC transplantation approaches. A second approach, induced transdifferentiation of RGCs from endogenous retinal cells, as exhibited by several non-mammalian species [16, 17], might obviate the need for transplantation, especially if such an approach can be harnessed efficiently and safely. Because the molecular mechanisms underlying RGC differentiation from pluripotent cells and endogenous retinal cells overlap, collaborative efforts focusing on RGC specification and maturation from stem cell differentiation and endogenous transdifferentiation provide an opportunity to advance the field (Fig. 1).

Although they do not yet recapitulate the mature retina, retinal organoids derived from pluripotent stem cells are a promising tool for broadening our understanding of RGC development in humans, as they mimic the development, structure, and function of the retina. However, RGCs that develop within organoids are often immature compared to their in vivo counterparts, tend to degenerate as the organoids age, and lack their postsynaptic target, limiting their usefulness for studying long-term RGC survival and maturation [18–20]. As we will discuss, the field would benefit from further identifying the mechanisms underlying RGC development and inevitable degeneration in retinal organoids. Knowledge gained will help researchers identify strategies to promote donor neuron survival and maturation for cell repopulation and will also lead to the development of new neuroprotective therapies for diseases affecting the retina.

### Müller glia transdifferentiation for RGC replacement

Retinal regeneration occurs naturally following damage or injury in some lower vertebrates, and recent advances have suggested genes important in this process [21, 22]. For instance, in teleost fish, this process is driven by Müller glia (MG), support cells that dedifferentiate into progenitor cells, proliferate, and replace damaged retinal neurons [23–26]. In mammals, this regenerative process is absent, and MG instead respond to injury by generating an inflammatory gliotic state [27]. Over the past decades, considerable advancements have been made in our understanding of the molecular mechanisms underlying MG transdifferentiation in regenerative species and neuroinflammatory gliotic responses in mammals, which may help translate endogenous regeneration strategies to humans. While MG transdifferentation can repopulate the outer retina, RGCs do not regenerate de novo after optic nerve injuries in zebrafish; rather they are highly resilient to primary degeneration [28], Therefore, this model may not be the most informative for RGC repopulation. Nevertheless, comparative species studies identified upregulation of a pro-neural transcription factor, *Ascl1*, in MG after injury in fish and birds but not mammals [29]. Overexpressing *Ascl1* in MG can stimulate the regeneration of functional neurons in adult mice after injury [30]. These cells express markers of bipolar neurons and wire into pre-existing retinal circuits. Conditional deletion of NFI_a/b/x_ factors also induces MG to generate cells expressing bipolar neuron markers, though it is unknown whether these cells are functional [31]. The addition of *Atoh1* in combination with *Ascl1* increases the number of new neurons generated through MG transdifferentiation [21]. Importantly, this is currently the only combination of transcription factors not requiring retinal damage to induce regeneration in the adult mouse retina [21].

Most regeneration models for RGCs are injury-based, where damage prompts a regenerative response. However, following an injury, RGCs exhibit signs of active cell death, which raises questions about the applicability of the findings for the repopulation of healthy RGCs. Studying teleost models, like zebrafish, in which RGCs are preserved and then shift into a regenerative state, may provide insights for improved regeneration in other organisms. Specifically, additional combinations of transcription factors have demonstrated enhanced potential for regenerating disease-relevant neurons in mammals. Induced expression of *Pou4f2*, *Isl1*, and *Ascl1* in MG was recently shown to produce RGC-like cells in adult mice after injury [22]. These newborn RGC-like cells transcriptionally resembled immature RGCs sequenced from the developing mouse retina and formed synapses within the inner retinal circuitry, but axon regeneration into the optic nerve and towards their cerebral targets has yet to be demonstrated. Though further work remains to demonstrate RGC subtype specification or long-distance axonal extension from endogenously regenerated neurons, these data suggest that RGC reprogramming from MG is achievable in mammals.

A critical goal in the study of RGC repopulation is to establish a definitive demonstration of inducing newly generated RGCs from endogenous neurons, which may have survived a neurodegenerative insult [32]. Typically, neural differentiation is defined based on the expression of reporter constructs meant to label only newly derived neurons. However, multiple factors can lead to artifactual labeling of endogenous host neurons with such reporters, which can prompt erroneous conclusions about transdifferentiation approaches to retinal neuronal repopulation. Such artifacts have arisen in studies based on AAV-mediated expression of transgenes, which are a common approach to inducing endogenous transdifferentiation of MG. A clear drawback of AAV-mediated retinal transduction from MG is that glial-specific promoters lose their cell type specificity when certain transcription factors are expressed, resulting in leaky expression within endogenous neurons [33–35]. This surprising result has led to dubious claims of MG reprogramming that remain to be validated with rigorous controls [36]. To avoid similar issues from impeding progress, the research community must be aware of these pitfalls and provide strong validations for RGC repopulation. Induction of MG transdifferentiation, using transgenic mouse lines rather than AAV, partially mitigates this concern [29], but using transgenic mice is not directly translatable to primate models. Other methods of verifying newborn cell origin involve lineage tracing using EdU and/or BrdU to label actively proliferating cells and track their differentiated progeny. However, these methods require that transdifferentiation occurs through a proliferative progenitor intermediary and would not label new neurons derived from direct transdifferentiation. Importantly, MG-derived neurons are rarely identical to normal retinal neurons in morphology, gene expression, or physiology, and these cells often retain a glial-specific transcriptional signature. Therefore, rigorous transcriptional, microscopic, and electrophysiological assays that document evolution over time can typically parse newborn neurons from endogenous retinal cells.

Although induction of endogenous regeneration in the rodent retina is possible, several questions must be answered before the translational potential of this approach can be appreciated. These include whether MG reprogramming is feasible in primates, including humans, whether the transcription factors capable of driving functional reprogramming of MG into RGC phenotypes are conserved among species, and whether there are any negative long-term effects of MG depletion after reprogramming. Designing a reprogramming system that is narrowly targeted or with less than 100% efficiency might be used to leave a percentage of MG unmodified to support retinal function and ameliorate the effects of MG depletion. Unfortunately, data describing the mechanisms underlying RGC specification in humans remain sparse due to limited tissue availability and ethical restrictions on studying human embryos. This limitation is further complicated because RGCs are the first neurons to appear in the retina, and early time points are particularly challenging to study [37]. Therefore, developmental studies of human retinal organoids will be important because they can provide answers to at least some of these questions.

### Sources of RGCs and models to study RGC development

Translational approaches to therapeutic RGC repopulation will require the study of human RGCs, but access to human and primate tissue is limited. Therefore, there is a need for relevant, in vitro methods of studying RGC specification, maturation, physiology, and survival. Stem cell-derived RGCs, 3D retinal organoids, and brain-retinal assembloids can support developmental research when human tissue cannot be readily accessed [19, 38, 39]. Direct differentiation of plated stem cells produces relatively pure RGC populations by converting pluripotent cells to neurons through activation of relevant transcription factors, such as *Neurog2, Atoh7, Isl1, Ascl1,* and *Pou4f2*, using small molecule signals or transfection [40–42]. In contrast, 3D organoids drive RGC differentiation by pushing stem cells through retinal development stages and yielding complex tissues with multiple interacting cell types. However, differences in RGC phenotype and maturation states have been identified when cells are derived by monolayer as compared to 3D culture approaches. For this reason, comparative studies are needed to determine which approach might be best suited for specific goals.

One potential source of RGCs for transplantation is to collect MG from donor retinas, as MG can transdifferentiate into RGCs [43]. However, the current limitations in maintaining MG in vitro for an extended period and the constraint of obtaining these cells from patients render them a less viable source for generating RGCs in vitro.

An alternative and more promising source of RGCs is pluripotent stem cells, which include both embryonic stem cells and induced pluripotent stem cells (iPSCs). These cells have the potential to differentiate into all cell types in the human body, and their ability to be maintained indefinitely in vitro makes them a potentially unlimited source for generating specific cell types, including RGCs [44]. Notably, patient-derived iPSCs, which can be reprogrammed from adult somatic cells, offer a unique advantage as they could be genetically and immunologically matched to the patient, increasing the potential for disease- or injury-based cellular therapies. Multiple studies have demonstrated the successful generation of RGC-like cells from differentiated rodent and human stem cells through direct and organoid-based differentiation [45–53].

While retinal organoids contain several RGC subtypes, including J-RGCs, alpha RGCs, intrinsically photosensitive RGCs (ipRGCs), and direction-selective ganglion cells [54, 55], RGC specification and maturation remain to be exhaustively characterized through multiple developmental states. Indeed, clarifying in greater detail the RGC subtypes unique to primates, including humans, is an important goal. Studies in rodents have identified RGC subtypes with particularly high resilience, susceptibility to optic nerve injury, and/or propensity for axon regeneration [6, 56]. Identifying correlates in primates may yield translational insights that could augment regenerative and/or neuroprotective paradigms. Critically, retinal organoids lack a fovea (which contains the highest density of midget RGCs), do not recapitulate topographic (i.e., dorsal–ventral, nasal-temporal) differences, and fail to form a retinal pigment epithelium layer as the retina does in vivo. Developing specific methodologies to specify RGC subtypes overrepresented in the macula and exhibiting regional heterogeneity may be necessary to restore high-acuity central and lower-acuity peripheral vision, respectively. Retinal pigment epithelium may be required to support retinal organoid maturation in vitro [57, 58], and it is conceivable that promoting outer retinal organoid development may drive secondary maturation of inner retinal circuits and RGCs. Supplementing 2D retinal cultures and 3D retinal organoids with other cell types, such as microglia, astrocytes, or pericytes, would likely also augment the development and maturation of retinal neurons.

Retinospheres are an alternative approach for the long-term in vitro culture of fetal retinal tissue to differentiate and study human RGCs [39, 59]. Retinospheres are generated directly from retinal fetal tissues rather than stem cells and maintain better lamination than retinal organoids [59], potentially providing a more physiologically relevant environment for RGC development. While single-cell sequencing has uncovered differences in gene expression between the fetal retina and retinal organoids, the signaling pathways that regulate RGC development in retinospheres remain largely undefined. However, despite their potential advantages, both retinospheres and retinal organoids suffer from the limitation that RGCs, more so than other retinal neurons, eventually die in culture [60], potentially due to the lack of retrograde transport of survival cues, such as brain-derived neurotrophic factor (BDNF), from central subcortical targets. To overcome this challenge, novel approaches, such as assembloids, which fuse 3D retinal and cerebral organoids, might be employed to increase the survival of RGCs in vitro [19]. Interestingly, RGCs can survive for lengthy periods in 2D culture; therefore, the lack of postsynaptic target innervation in retinal organoids may not independently explain the improved RGC survival documented in brain-retinal assembloids. Further investigations into the factors that support RGC survival and differentiation across in vitro culture models will be essential to developing effective cell replacement products for optic neuropathy.

### RGC specification

Various signaling pathways tightly control RGC specification, patterning, and differentiation during development. Several key transcription factors are involved in RGC fate determination, including *Atoh7*, *Isl1*, and *Pou4f2* [7, 61–65]. *Atoh7* is expressed early in RGC development and is essential for RGC differentiation and survival [61]. *Isl1* is a critical transcription factor that regulates RGC fate, while *Pou4f2* controls RGC survival, maintenance, and fate [66]. Whereas transcription factors that maintain RGC fate are well described, much less is known about factors repressing RGC specification. While the Sonic Hedgehog pathway regulates RGC numbers during development by shifting progenitor cell differentiation towards other retinal cell types [67], a more comprehensive understanding of the gene regulatory networks that positively and negatively influence RGC specification would improve our ability to generate this cell type efficiently.

Extrinsic mechanisms, including signals from neighboring cells and extracellular matrix components, also play a crucial role in RGC differentiation and survival. For example, neurotrophins, such as BDNF, promote RGC survival and neurite outgrowth [68, 69]. Laminin at high concentrations in basement membranes, including the internal limiting membrane (ILM), promotes RGC differentiation, polarization, and axon guidance [70–72]. These findings suggest that a complex interplay between intrinsic and extrinsic factors regulates RGC specification and differentiation, which might indicate that incorporating a more complex signaling milieu could enhance RGC generation from pluripotent sources.

Epigenetic modifications are essential for regulating gene expression and cellular differentiation, including RGC development, plasticity, and survival. For example, histone deacetylase inhibitors drive RGC differentiation and promote neurite outgrowth [73, 74]. Furthermore, resetting the epigenetic modifications associated with aging in RGCs induces significant endogenous RGC regeneration in injury models [75]. Moreover, following *Pten* knockout, ipRGCs downregulate several genes, including those involved in subtype specification, indicating that subtype specification is not a determinant of intrinsic regeneration capacity but rather a function of proximity to a particular transcriptomic state [76]. In fact, ipRGCs are more similar to the embryonic RGC transcriptome state compared to other subtypes, and *Pten* knockout causes ipRGCs to become even more transcriptomically similar to embryonic RGCs and have a greater regenerative capacity as a result [76]. Such approaches to “reset” or dedifferentiate RGCs may help identify molecular targets to prime RGCs for repopulation.

In addition to expressing RGC-specific genes, it may be relevant whether donor cells "match" the recipient’s age through similar epigenetic modifications. RGCs derived directly from fibroblasts retain the age of the original cell [77], whereas RGCs derived from iPSCs are epigenetically "younger." It remains unclear how relevant epigenetic state or biological clock “age” is to the ability of newborn RGCs to integrate into retinal circuitries, and this represents an interesting question for further research.

In sum, a more comprehensive understanding of the positive and negative genetic and epigenetic regulators of RGC specification and maturation will likely improve protocols aimed at generating RGCs that are highly efficient at integrating into recipient retinas and functioning within the visual pathway.

### RGC subtypes and vulnerability (susceptibility) to injury

RGC diversity is necessary to process the various features of visual information (i.e., motion, color, direction, contrast, and non-vision forming inputs to subnuclei entraining circadian signals) that comprise our perception of the world. Multiple RGC subtypes have been identified in the retina of various species using physiological, morphological, and molecular criteria [8, 78]. RGC subtypes also form distinct postsynaptic connections with different retinorecipient brain targets. However, it remains undetermined whether all these RGC subtypes need to be re-established to restore visual function after optic neuropathy or if some rudimentary vision restoration is possible by regenerating only the most prevalent or functionally critical subtypes. RGC subtype heterogeneity has been shown to underlie visual functions in mice and zebrafish [79, 80], but these relationships have yet to be rigorously explored in other species, including humans and non-human primates.

The numbers of RGC subtypes vary dramatically across species, and it remains unclear which populations of cells should be regenerated/transplanted to reverse blindness. For instance, 30–60 RGC subtypes have been identified in mice compared to only 18 in macaques and humans [8]. Moreover, the distribution and frequency of these subtypes vary across species [81]. In humans, more than 86% of RGCs are midget types (with roughly equal distributions of ON and OFF subtypes) [82], while in mice, the most frequent RGC subtypes (W3 RGCs) comprise only ~ 7.5% of all RGCs [81]. From an evolutionary perspective, animals with a high-acuity visual system may have whittled away such vast RGC subtype diversity over time in favor of greater central processing of visual stimuli [83]. Moreover, unique retinal characteristics, such as foveation in primates, may underlie this RGC subtype variation among different species. While single-cell transcriptomic studies have identified key species differences in subclasses of RGCs, some studies may have underestimated the number of RGC subtypes due to limited RGC capture, as RGCs typically represent less than 1% of cells in the adult retina [84, 85]. Therefore, further evolution in single-cell transcriptomics will likely increase sensitivity and identify greater complexity among RGC subtypes, especially during development or disease.

One issue underlying apparent discrepancies in reported distributions of RGC subtypes relates to the multiple methods by which subtypes have been defined. Recent efforts have been made to unify the classification of RGC subtypes across morphologic, electrophysiologic, and transcriptomic modalities [79]. Advancements in techniques like Patch-seq enable simultaneous transcriptomic and physiological profiling of cells [86]. Spatial transcriptomics at cellular resolution can also be leveraged to capture subtype differences on both the morphological and transcriptomic levels [87], using platforms such as 10 × Xenium (https://10xgenomics.com), MERFISH [88], and NanoString GeoMx (https://nanostring.com). For example, MERFISH has been applied to the retina as part of the Cell Atlas Eye [89]. Additional questions, such as whether specific RGC subtypes are preferentially lost with age and whether RGC subtypes differ based on retinal location and sex, may also be answered through these technologies.

A crucial but unresolved question in glaucoma and other optic neuropathies is whether and why distinct subtypes of RGCs are vulnerable to damage and, therefore, preferentially lost in these conditions. Topographical patterns of RGC loss corresponding to visual field defects characteristic of specific optic neuropathies have been appreciated for decades. The earliest RGCs to exhibit degeneration in glaucoma tend to be localized to the temporal retina and correspond to a “nasal step” defect [90]. In contrast, toxic and nutritional optic neuropathies are classically defined by cecocentral defects arising from the loss of macular RGCs. While there are some morphological and functional differences in RGCs based on their topographical location, specific transcription factors have also been associated with ipsilateral/contralateral targeting segregation [59], retinal eccentricity, and axonal location within the optic nerve [91], which may also underlie potential differences in vulnerability.

Whether RGC subtypes exhibit differential vulnerability to optic nerve insult independent of topographic location remains to be determined. Selective vulnerability of human RGC subtypes in glaucoma was first reported in the 1980s and described as a preferential loss of RGCs with larger somas and axon diameters [92, 93]. Subsequently, numerous other studies evaluated the selective loss of human RGCs in glaucoma [94–108], of which more than half failed to identify such RGC type-specific loss [101–108]. Interestingly, only one of the contradictory reports evaluated the loss of RGCs in glaucomatous tissues using histological approaches [108], suggesting that methodological differences may have contributed to conflicting conclusions. Indeed, both anatomical and functional studies of non-human primates on the selective vulnerability of RGCs in glaucoma have remained inconclusive. Specifically, RGCs with larger somas and axon diameters may be preferentially vulnerable in glaucomatous monkeys [109–116] and pigs [117]. On the other hand, results from multiple functional and anatomical studies showed the opposite, suggesting a universal exposure of RGC subtypes in nonhuman primate models of glaucoma [118–124].

Subsequently, an overwhelming number of studies in cat [125–130], rat [131–138], and mouse [81, 139–156] glaucoma or other RGC degeneration models—spanning from 1989 to 2020—reported significant variation among RGC subtypes in their response to experimental perturbations including elevated IOP, optic nerve trauma, and excitotoxicity. Few mouse studies have failed to identify a selective vulnerability of RGC subtypes in glaucoma [157, 158]. The vulnerability of RGC subtypes in mouse and other animal models of RGC degeneration (e.g., hypertensive microbead injection glaucoma model, optic nerve crush model, and DBA/2 J mice) appears well established. For instance, direction selective-RGCs and alpha-RGCs are more vulnerable after an injury in mice, while ipRGCs are more likely to survive than other subtypes [138]. Spatially, the mid-peripheral and peripheral RGCs are more vulnerable in experimental glaucoma in pigs and rats [117, 159, 160]. Interestingly, there is also an increase in the RGC soma size in experimental glaucoma [117, 159, 161, 162]. The propensity for selective loss of RGC subtypes in humans (and non-human primates) requires further clarification. Although new technologies, like single-cell RNA sequencing technologies, have greatly facilitated the identification and quantification of molecularly distinct subtypes, answering this question in humans will be challenging due to variations in the severity (stage) of various optic neuropathies and availability of tissues, which enable only cross-sectional evaluations.

For several reasons, it is necessary to understand whether human RGC subtypes differ in their vulnerability to damage. First, the molecular and cellular features of susceptible and/or resistant RGCs could be identified and leveraged to develop molecularly targeted neuroprotective treatments. Second, RGC subtype specificity could influence the design of therapeutics to repopulate specific RGC subtypes in the eye [163, 164]. Finally, understanding the molecular mechanisms underlying RGC subtype susceptibility to optic nerve damage would significantly advance our understanding of disease pathophysiology [81]. Indeed, care must be taken not to promote the regeneration of one RGC subtype at the expense of a more vulnerable, but functionally necessary, population.

Several experimental approaches have been proposed to identify which RGC subtypes are most relevant to target from a therapeutic standpoint. These include individually depleting all but one RGC subtype in the retina through transgenic approaches to better understand isolated subtype function, prioritizing the study of rare diseases preferentially affecting specific RGC subtypes, using transgenics to more comprehensively characterize RGC subtype development and functions [165], and performing highly specific visual modality testing in patients with optic neuropathy to identify potential subtype-specific deficits [166]. In addition to commonly used automated perimetry, other assessments such as contrast sensitivity, direction sensitivity, color perception, and specialized electroretinography (ERG) metrics might complement visual fields in measuring RGC subtype-specific functions. Specifically, contrast and color vision tests directly measure specific attributes of RGC function and subtype specification [166]. However, their added value and potential for predicting disease progression earlier than visual fields alone require further study, and any value in integrating these tests with conventional perimetry in routine glaucoma practice rather than for research purposes remains to be determined.

### Future directions for RGC development and differentiation

Over the past ten years, methodologies have significantly expanded for driving RGC differentiation from stem cells. These protocols hold great promise for developing cell-based therapies for retinal degenerative diseases, such as glaucoma. However, which protocols are best suited to generate RGCs for experimental or therapeutic goals remains to be determined. We propose that direct comparisons (based on morphology, transcriptomics, and electrophysiology) of human RGCs generated through independent approaches (e.g., gradual differentiation in monolayers, directed differentiation in monolayers, and within retinal organoids) would be highly informative. Furthermore, comparisons of RGC engraftment following transplantation into the eye using these various sources could help elucidate the best source for donor RGCs.

One key question is whether it is necessary to replicate progression through all stages of RGC development, as occurs in retinal organoids, or if it is possible to bypass specific developmental steps and directly induce RGC differentiation using vectors that drive the expression of specific transcription factors. It is unknown if doing so would enable subtype specificity, but recent data suggest that identifying and manipulating the molecular and cellular factors that regulate RGC subtype development in vivo may offer a solution [167]. This approach might involve single-cell transcriptomics to identify subtype-specific gene expression profiles and manipulate signaling pathways involved in RGC subtype specification in vitro and in vivo. If achieved, directed differentiation of RGCs into specific subtypes would provide a valuable tool for shedding light on the selective vulnerability of RGC subtypes in humans and could also be critical to repopulating the most visually relevant RGC subtypes in patients. Therefore, developing novel methods for controlled subtype specification of stem cell-derived RGCs is a crucial area for future research.

Establishing protocols to generate RGC subtypes may yield targeted interventions to restore specific aspects of vision. For example, the fovea subserves high acuity central vision and contains a high density of midget RGCs, for which no specific differentiation protocol yet exists. Developing a fovea in organoids is under investigation in several labs and may help address this challenge. Although increasing our knowledge of RGCs subtypes is crucial for transplantation-based RGC repopulation, an alternative might involve engineering a ‘generic’ RGC that would be robust and resilient once integrated into the host injured environment. However, the ultimate level of visual function that such an RGC could support remains unclear. Ongoing work in optogenetics and implantable visual prostheses may partially answer this question since current technologies activate RGCs indiscriminately, without regard for their subtype.

Access to primary retinal tissue from human postmortem donors would help validate whether these findings are relevant to patients. According to recent work, primary neurons from human postmortem donors can survive in specially designed culture conditions for up to 6 weeks [168]. Using this tissue, human-derived-MG cultures could be used to screen a large set of transcription factors and focus on the most competent ones for RGC reprogramming before performing more complex experimental tests in vivo. For in vitro screens, relative transcription factor expression levels must be considered. The ability to study human cells in culture could help answer whether gene signaling networks underlying regeneration are evolutionarily conserved. However, studying species close to humans, like non-human primates, represents an experimental alternative.

In summary, various models exist for studying RGC development and generating this class of neurons for experimental and therapeutic purposes, each with advantages and limitations. Stem cell-derived RGCs, 3D retinal organoids, retinospheres, brain-retinal assembloids, and dissociated primary cultures are helping investigators to better understand RGC biology and develop cell-based therapies for retinal degeneration. However, further research is necessary to improve these models and ensure their relevance to human vision disorders (Table 1).

**Table 1:** Future directions for RGC development and differentiation (SDG1)

Research Area	Future Goals
RGC subtype identification	Identify the human RGC subtypes most affected by diseases like glaucoma and study the underlying mechanisms of their vulnerability. Prioritize research into diseases that might give insights into RGC subtypes most relevant from a therapeutic standpoint
RGC differentiation protocols	Determine how bypass of developmental differentiation stages with directed induction of RGC specification may influence or enable subtype specification
Subtype-specific gene expression profiles	Use cutting-edge transcriptomic technologies to identify subtype-specific gene expression profiles across species and manipulate relevant signaling pathways to better understand RGC subtype specification in vitro and in vivo
Subtype-specific transplantation protocols	Develop protocols for RGC subtype-specific differentiation to advance the study of RGC subtype biology and pathophysiology, and to enable subtype specific transplantation protocols
Fovea in organoids	Develop a methodology for generating foveated human retinal organoids, which would be scientifically valuable and may also provide an efficient way to generate midget RGCs, which could support high acuity vision if transplanted
Glial reprogramming	Use human stem cells or postmortem tissue to derive MG for screening a large set of transcription factors for RGC reprogramming, followed by further evaluation in human retinal organoids and/or nonhuman primates

## SDG #2. Transplantation methods and models

**Fig. 2:**
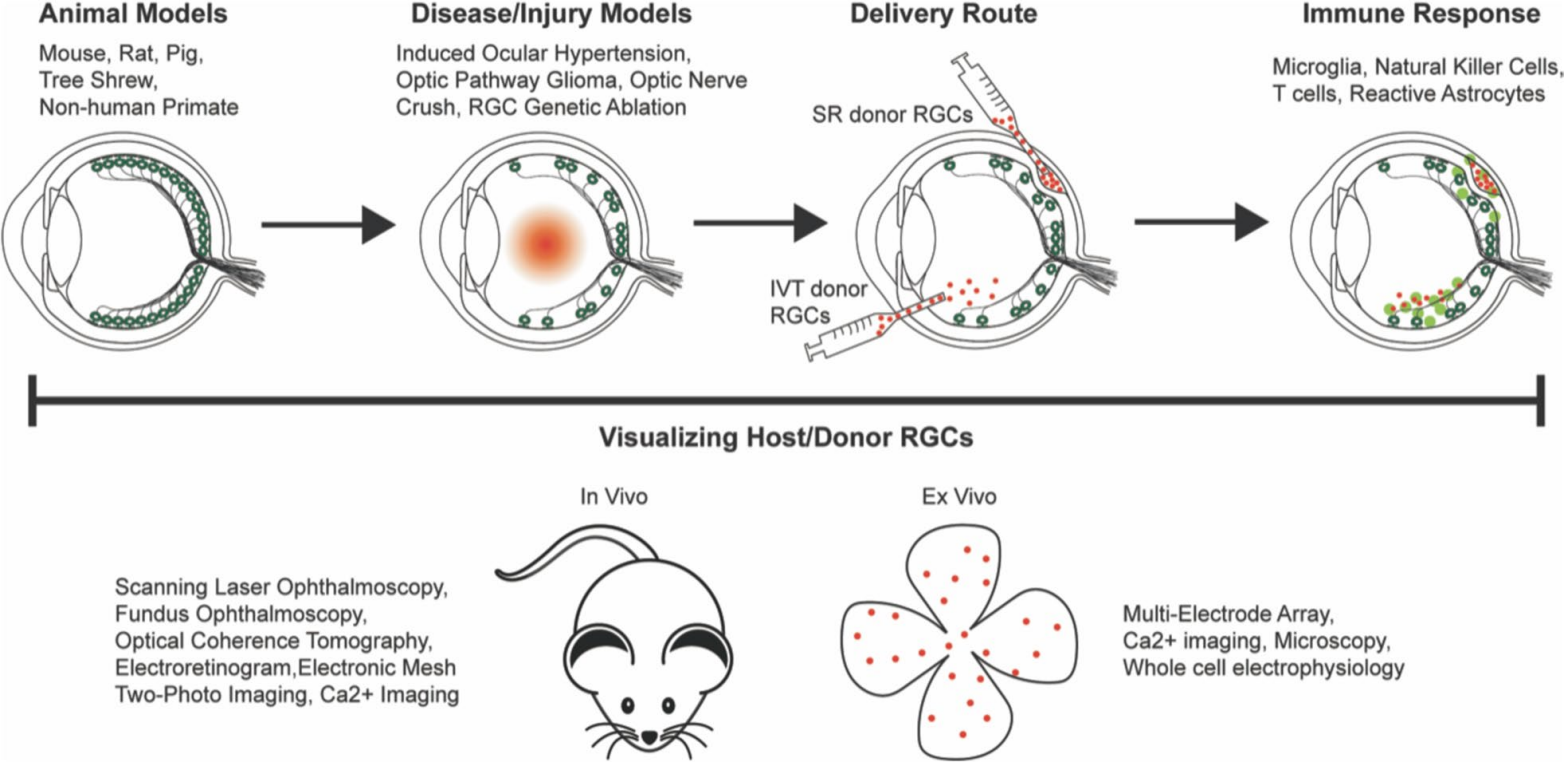
RGC transplantation models, methods, and assessment. Each animal and disease/injury model possesses advantages and disadvantages for studying essential aspects of RGC replacement and mimicking different characteristics of optic neuropathies. Donor RGCs can be delivered to the intravitreal (IVT) or subretinal (SR) space, but each route has unique barriers to overcome to achieve structural integration. In addition to integrating within the host retina, donor RGCs must avoid being targeted by the adaptive and innate immune systems. Visualizing donor and host RGCs is essential to translate cell replacement therapies to the clinic, and a combination of techniques is required to properly assess the structural and functional integration of the transplanted cells

Once exogenously derived RGCs are introduced into a diseased eye, many significant obstacles must be overcome to yield survival and engraftment of large numbers of donor neurons. Lessons learned from the retinal pigment epithelium and photoreceptor transplantation fields inform some approaches to achieving these goals. Animal models remain essential to studying and developing treatments for human diseases (Fig. 2). These preclinical models must recapitulate human anatomy, physiology, and genetics to the greatest extent possible as they relate to optic neuropathy pathophysiology and cell transplantation methodology. Each model has unique advantages and disadvantages for answering experimental questions in a clinically translatable manner, and some models are better suited than others for studying aspects of RGC transplantation. Rigorous efforts utilizing multiple independent disease models and species have the highest potential for avoiding disappointing results in human clinical trials.

### Preclinical animal models

Given that the recipient microenvironment influences the survival and behavior of transplanted neurons, consideration of specific optic neuropathy models for evaluating RGC transplantation is critical. Many distinct inherited, traumatic, excitotoxic, and ocular hypertension-induced RGC loss and damage models have been developed [169, 170]. RGC cytotoxicity induced by NMDA, glutamate, or kainic acid is rapid, severe, and exhibits low variability, but their clinical relevance is limited by the mechanism of action and concomitant loss of inner retinal neurons beyond RGCs [171, 172]. Traumatic injury to the optic nerve, including transection, crush, and ocular blast injury, is more clinically relevant but leads to profound and sometimes variable RGC loss.

Optic neuritis and ischemic optic neuropathy models represent other clinically relevant strategies to cause the death of RGCs [173–175]. Optic neuritis occurs in the murine model of multiple sclerosis, wherein myelin oligodendrocyte glycoprotein is administered to induce experimental autoimmune encephalomyelitis [176]. Ischemic optic neuropathy can be induced in rabbits using a sustained endothelin-1 release via minipump [177]. Alternatively, selective capillary occlusion at the optic nerve head can be accomplished in both rodents and non-human primates by the intravenous injection of a photosensitive dye (e.g., Rose Bengal) in conjunction with low-intensity laser light to generate dye-induced superoxide radicals that circulate within the optic nerve capillaries [175, 178].

Ocular hypertension-induced optic neuropathy is most relevant to human glaucoma. This form of optic neuropathy can be achieved in multiple species through inherited or virally transduced gene mutations (e.g., DBA/2 J, Myocilin, Angiopoietin, TGFβ, etc.) [179, 180], intracameral microbead or silicon oil injection, laser photocoagulation of the trabecular meshwork, and episcleral vein cauterization or sclerosis via hypertonic saline injection [181–183]. Importantly, more than 30% of glaucoma patients experience progressive optic neuropathy despite intraocular pressure (IOP) in the normal range (≤ 20 mmHg). Corresponding models of “normal tension” glaucoma have been developed [184, 185], such as through mutation of the *Optn* gene, which encodes an adaptive protein involved in vesicle trafficking and autophagy [186]. These models differ in the timing and extent of IOP elevation and the degree of endogenous RGC loss, enabling evaluation of neuronal transplantation in various glaucoma-like pathological contexts.

Rather than inducing active neurodegeneration, mouse strains exhibiting developmental defects in RGC specification also offer an opportunity to evaluate the influence of endogenous RGCs on donor engraftment. Knockout of *Brn3b (Pou4f2)*, *Brn3a (Pou4f1)*, and *Math5 (Atoh7)* leads to a developmental depletion of RGCs [187, 188]. These models provide an opportunity to test the hypothesis that endogenous RGCs impact donor RGC integration without the confounding effects of an actively neurodegenerative retinal environment, which is inherently accompanied by chronic neuroinflammation and may be a hostile environment for donor cells.

Additionally, murine models of optic glioma provide opportunities to study chronic axonal injury, RGC degeneration, and vision loss. Leveraging conditional transgenesis approaches, *Neurofibromatosis type 1* (*Nf1)*-mutant mice have been genetically engineered to develop optic nerve gliomas, which subsequently result in RGC death and impaired performance on virtual optokinetic system testing [189, 190], mimicking the clinical progression of optic pathway gliomas (OPGs) and associated vision loss in NF1 [191]. Understanding the cell-intrinsic [192] and cell-extrinsic [193] molecular and cellular events that culminate in RGC loss may yield new insights into therapeutic approaches for this and other chronic optic neuropathies [194].

With their genetic and anatomical similarities to humans, zebrafish are another valuable model for studying vision loss [195] and various therapeutic interventions [196]. Specific zebrafish mutants, such as brass and bugeye, exhibit chronic elevated IOP, resulting in symptoms akin to glaucoma, including retinal stretching, RGC loss, and progressive optic nerve damage [197]. Detailed investigations into these models have promoted ou understanding of the genetic underpinnings of glaucoma, such as the involvement of the low-density lipoprotein receptor-related protein 2 gene in the bugeye mutant [197, 198]. Understanding the behavior of RGCs under elevated IOP conditions and the mechanisms of RGC death in these models can provide insights into therapeutic strategies to prevent RGC loss or promote their recovery. Additionally, due to the transparency of zebrafish embryos, the process of RGC development, death, and replacement can be directly visualized, offering an opportunity to assess multiple aspects of RGC biology in vivo, including function and subtypes [80].

Each model and species present advantages and disadvantages for studying various aspects of RGC replacement, including cellular derivation, formulation, and dose; surgical methodologies; immunogenicity and tumorigenesis; and neural circuit integration. Ex vivo tissue culture models and retinal organoids enable high throughput experiments with significant microenvironmental control at the expense of enabling long-term studies or incorporating the peripheral immune or vascular systems. Nonetheless, postmortem human retinal tissue and human retinal organoids enable studies of donor RGC integration into clinically relevant host tissue.

Mice are advantageous as transplant recipients because they are broadly accessible, inexpensive, and have been the subject of many inducible and developmental optic neuropathy models [199]. However, several fundamental differences between human and murine ocular anatomy represent drawbacks to their translatability. Rodent retinas do not contain a macula [200], and their RGC subtypes and inner retinal wiring patterns differ substantially from primates, including humans [8, 82]. Furthermore, rodents possess a glial, rather than collagenous, lamina cribrosa, which is thought to be a critically important site for glaucomatous injury of RGCs in human and non-human primate eyes. As donor RGCs will need to extend axons through the optic nerve head to reach subcortical visual targets, it may be necessary to study axonal pathfinding and efferent synaptogenesis in species with a collagenous lamina cribrosa. While remodeling of the lamina cribrosa is a hallmark of glaucoma in humans [201], it is unknown if there are differences in the ability of RGC axons to regenerate through a collagenous vs. a non-collagenous structure before or after remodeling. It may also be important to understand differential effects on axonal regeneration and pathfinding of varying stages of connective tissue remodeling and reactive gliosis within the optic nerve head, just as the microenvironment of the retina will be important for survival and integration of replacement RGCs.

Ocular size is another vital consideration for cell replacement experimentation and eventual translation to human eyes. Though cell suspensions can be injected into the vitreous cavity of rodents, larger animal models are amenable to clinically relevant techniques such as pars plana vitrectomy, ILM peeling, and intravitreal (IVT) implantation of semi-rigid scaffolds.

Cell replacement in non-human primates may be the best predictor of vision restoration in humans and represent the best model to investigate translatable cell delivery strategies, but they are often cost- and resource-prohibitive at the early stages of research. The tree shrew is a para-primate and an alternative animal model with distinct advantages over the mouse, non-human primate, and ex vivo models [202]. Tree shrews have been bred in captivity, have a collagenous lamina cribrosa, exhibit excellent ocular optics enabling high-resolution noninvasive ophthalmoscopy, and have been subject to well-characterized models of optic neuropathies [203]. However, tree shrews have complex colony management requirements, can be challenging to work with, given their temperament and small eyes, and their collagenous lamina cribrosa is structured differently than in primates, including humans. Further, molecular and biochemical reagents to study tree shrews have not been well characterized.

In addition to non-human primates and tree shrews, domestic animals such as cats, dogs, and pigs exhibit anatomical and physiologic ocular parameters relevant to human glaucoma and other optic neuropathies and RGC replacement therapies. For example, both beagles and cats can be affected by glaucoma spontaneously in some lines [204, 205]. Experimentally induced optic neuropathy models in cats closely recapitulate the optic nerve pathology of human glaucoma [206–208]. Furthermore, pigs have similar eye anatomy, vasculature, and photoreceptor distribution to humans [209], and RGCs in the pig retina have been well described [210–212]. Moreover, there are several well-characterized models of experimental glaucoma in pigs [117, 160] that exhibit changes in the retinal microvasculature [213] and trabecular meshwork that are similar to humans [214, 215].

Besides differences in ocular anatomy between models, differences in the retinal microenvironment may be relevant to donor RGC growth, maturation, axon extension, and metabolic demands. Each model used to study cell replacement therapies will provide insights into controlling the retinal microenvironment in optic neuropathies to support successful donor RGC grafting. Studying various animal species, specifically non-human primates, can provide important information on immune compatibility and potential rejection issues, which must be addressed before conducting human trials. Due to their genetic versatility, rodents offer fundamental insights into basic mechanisms. Pigs are valuable for disease models due to their similar ocular anatomy and larger eye size, making it easier to perform surgical techniques and transplantation methods. However, larger animals are more difficult and expensive to house.

To establish safety, the first patients to be enrolled in RGC transplantation trials will likely be those with advanced optic neuropathy and severe to complete vision loss. However, subsequent efficacy studies must consider and target the patient populations most likely to attain functional improvement, and preclinical experimental work would benefit from studying animal models of analogous diseases. To this end, there is a rationale to conduct early RGC replacement clinical trials in patients with an orphan disease, such as NF1-OPG [216]. In this case, younger patients with pathology primarily localized posterior to the optic nerve head may provide a more favorable environment for donor RGC integration into the retina and could exhibit enhanced neural plasticity. While models of NF1-OPG are highly variable in their presentation, improving upon the current models by varying the cell of origin [217] or genetic mutation [218, 219], as well as developing NF1-OPG models in different species, like pigs [220], to study RGC replacement may enable these therapies to enter the clinic with fewer regulatory hurdles if given an orphan disease designation. Alternatively, there are many phenotypes of glaucoma, and patients with greater intrinsic RGC susceptibility (such as those harboring the OPTN-E50K mutation) might be particularly favorable subjects for early RGC replacement therapies based on the hypothesis that cell-extrinsic pathogenic drivers may be less active in such patients and wild type RGCs transplanted into this environment may exhibit greater survival.

Lastly, acute ischemic optic neuropathies may represent favorable substrates for RGC replacement since the insult is typically acute and often occurs in relatively younger patients. In zebrafish, signaling between retinal glia and the endothelial cells that comprise the blood vessels in the retina is necessary for retinal regeneration [221]. Therefore, perhaps patients with significant neurodegeneration yet good blood flow, slow or remote (rather acute and active) neurodegeneration, low levels of chronic inflammation, and well-controlled IOP at the time of transplant will yield the greatest graft success. Standardizing the “ideal” optic neuropathy patient to mirror in an experimental model is of great significance going forward.

In addition to selecting the “ideal” experimental model to study RGC repopulation, it is essential to investigate the long-term effects of various interventions before they can be used in patients. While some studies suggest that donor RGCs survive for > 1 year in rodents [54], most transplantation studies have focused on shorter time points (up to 3 months) [222]. More studies need to be designed to include later-stage time points after treatment. These studies may be included as a part of the principal research or as flow-up reports to better facilitate the rapid dissemination of promising results.

#### Graft specifications

Previous studies show that the survival rate of intraocular primary RGC transplants is around 1% [10, 223]. Although the survival rate is relatively low, transplanting more neurons may not necessarily increase donor cell survival. Indeed, more donor RGCs survived in one study when 40,000 cells were injected intravitreally in rats compared to 60,000 cells [10]. There are several possible reasons for this outcome. First, transplanting a greater number of cells into the volume-limited rodent eye requires increasing the cell density of the payload, which may lead to cell aggregation and can increase mechanical shear stress on the neurons as they pass through the needle and/or result in clumping rather than dispersion on the host retina. Second, the metabolic burden from donor cells may worsen the existing metabolic deficiency in pathologic conditions. Transplanting too many donor cells may further deprive the host retina of nutrients, harming the donor cells and remaining host RGCs [224]. Finally, more donor cells may induce a greater local and systemic immune response. Therefore, further investigation is required to determine the optimal number of donor cells that should be transplanted across the range of model species.

Most prior studies transplanting RGCs have injected cells purified using RGC-specific markers [222]. However, there is precedent for non-RGC cells contained within a graft as beneficial to support the health and integration of the target RGC population [225]. Depending on the various differentiation protocols or development stages, it might be advantageous to transplant the donor cells as a mixture of RGCs and other cells, such as retinal microglia, astrocytes, MG, and/or retinal progenitor cells and semi-differentiated retinal ganglion neuroblasts, rather than pure RGC populations. RGC-microglia and RGC-astrocyte cocultures have led to more robust functional responses and complex morphology in RGCs [226, 227]. Hence, whether it is necessary or optimal to purify the donor cells before transplantation remains to be determined. Furthermore, donor cells must have limited proliferation ability to obviate the risk of tumorigenesis regardless of the preparation protocol.

### Transplantation timing

Cell replacement therapy requires careful consideration of the timing, both in terms of the recipient’s disease stage and the developmental stage of donor cells. Some optic neuropathies, such as glaucoma, pose a particular challenge in terms of treatment timing as the disease is often chronic and disproportionately affects elderly adults. The aging retina undergoes para-inflammatory dysregulation, and alterations in microglial phenotype contribute to neurodegeneration [228]. Furthermore, aging-related oxidative stress accumulation promotes neuroinflammatory dysregulation of the nervous system, possibly contributing to neurodegeneration [228, 229]. An inflammatory microenvironment might be necessary for regeneration [230], but it also might negatively affect the survival and integration of donor neurons.

Moreover, RGC death is heterogeneous in glaucoma. Advanced glaucoma is associated with reactive gliosis, scarring, and biomechanical remodeling of the optic nerve head, possibly hindering engraftment. Therefore, it appears critical to rigorously explore the influence of recipient age and disease stage in clinically relevant animal models of optic neuropathy on the efficacy of RGC transplantation. Most rodent models commonly used in RGC transplantation studies are 2–6 months old [231], roughly equivalent to a 20- to 30-year-old human. In mice, the incorporation of donor brain progenitor cells seems to decrease as host age increases [232]. In addition, neural progenitor cell transplantation in the Brazilian opossum retina demonstrates more extensive survival and morphological integration in the developing retina (postnatal 15 and younger) than in older retinas (postnatal 35 and older) [233]. Interestingly, a study of photoreceptor transplantation showed greater cell integration in older mice [234]. However, older mice with retinal degeneration exhibit greater photoreceptor loss, which confounds the association with age alone. Thus, it remains to be determined whether greater photoreceptor integration is due to age or degenerative state, and it is critical to explore this association for RGC replacement in aged animals.

Many human patients blind from optic neuropathy seen as potential RGC repopulation candidates are elderly. Moreover, the first-in-human RGC transplantation procedures will likely be in individuals with late-stage vision loss and no light perception. Consequently, exploring the anatomical and physiological changes in models with severe optic neuropathy and how later disease stages relate to engraftment will be necessary.

Some models, like optic nerve crush, induce rapid and robust RGC death, while others, such as microbead-occlusion and silicon oil-occlusion models, cause sub-acute RGC injury, ultimately resulting in the loss of only a minority of RGCs. However, none of these models fully mimic the natural disease progression or fully recapitulate the role of immune reaction and gliosis in human pathology. A combination of multiple disease models is likely required to comprehensively understand how transplanted RGCs respond to the heterogeneity of human disease.

In preclinical models, optimizing the timeline is crucial for successful engraftment. As optic neuropathy advances, the retina may become less receptive to engraftment due to reactive gliosis, scarring, and optic nerve head changes. While achieving RGC engraftment in the early stages of optic neuropathy may be more practical, studying engraftment in end-stage disease on aged animals is vital for translational purposes. For instance, treatments could significantly differ in terms of efficacy and off-target effects as a function of recipient age. Moreover, topographically heterogenous RGC loss occurs in many optic neuropathies and requires validating that attempts to repopulate new RGCs do not disrupt the survival or function of surviving, endogenous RGCs. Understanding the role that host cells and environmental changes play in engraftment will enable RGC transplantation optimization, thereby enhancing its efficacy in both experimental preclinical models and clinical translation to human patients.

### Transplant location and technique

Donor neurons must be transplanted in a manner that facilitates migration into the retina, elaboration of dendrites within the inner plexiform layer, formation of synapses within specific retinal circuits, and axonal growth through the optic nerve before visual function can be restored. Delivery location is a fundamental consideration for RGC transplantation that will influence the feasibility of meeting these challenges.

The subretinal (SR) delivery route is the standard for photoreceptor transplantation, as it provides direct access to the outer retina and sequesters donor neurons near the retina in a metabolically supported space between the photoreceptors and retinal pigmented epithelium [235]. However, while SR transplantation provides reliable donor-host contact, this procedure results in retinal detachment (at least locally) and causes secondary injury to the native photoreceptors. While this may be acceptable for treating outer retinal degeneration, it is challenging to rationalize induction of photoreceptor injury for treating optic neuropathies wherein the outer retinal is otherwise largely intact. The larger the volume that is injected into the SR space, the greater the extent of photoreceptors that are damaged, which limits the volume and number of donor RGCs that can be delivered via this route. Regardless, donor RGCs transplanted in the SR space must migrate through the host retina to arrive at the ganglion cell layer (GCL), which may decrease integration efficiency. For SR delivery, the RGC payload would also be sequestered topographically to the extent of the SR bleb, necessitating lateral migration if RGCs are to be replaced more peripherally within the retina. Methods to address this challenge are under development [236]. Specifically, by establishing an exogenous stromal cell-derived factor-1 gradient within the retina through IVT delivery of the purified protein, donor RGCs can be stimulated to migrate into the inner retina in vivo following SR delivery [236]. However, the efficiency of trans-retinal migration and potentially deleterious effects on host retinal circuits of that intraretinal migration remain unclear.

IVT or epiretinal transplantation can circumvent these obstacles and provide the shortest route for donor cells to migrate and integrate into their ultimate anatomical location [222]. However, donor RGCs may require more direct metabolic support after delivery into the vitreous cavity because of the relative extent of hypoxia in that compartment. Moreover, they must navigate through the highly viscous vitreous known to limit nanoparticle displacement [237], potentially hindering donor RGC migration into the retina. Although the vitreous cavity is a relatively immune-privileged site, it is less immune-privileged than the sub-retinal space, and peripheral immune cell infiltration can and does occur following intraocular surgery [238, 239].

Intravitreally transplanted cells also face the obstacle of the ILM, a basement membrane composed of extracellular matrix proteins such as laminin and collagen IV. The ILM is a physical barrier to transplanted stem cells, viral vectors, therapeutic compounds, and nanoparticles [240, 241]. By impeding donor cell migration, the ILM can block RGCs from engraftment. Indeed, developmental ILM dysgenesis and enzymatic digestion of the ILM are associated with increased donor RGC survival, retinal localization, and dendrite lamination within the inner plexiform layer (IPL) [242, 243]. Though enzymatic ILM degradation has been successful in rodents, there may be a better approach for clinical translation since intravitreal enzymes can elicit an inflammatory response and may cause off-target retinal toxicity. Fortunately, the ILM is routinely surgically peeled in human patients to treat macular holes and vitreomacular traction, providing a translatable approach to enhancing RGC engraftment. Indeed, surgical ILM removal in large animal models is feasible [244]. As an alternative to *en bloc* ILM removal, laser photoporation is being developed to create focal, precisely patterned ILM defects in a manner that obviates underlying neuroretinal damage and elicits minimal immune response [245].

Despite ILM disruption or removal potentially increasing RGC integration following IVT transplantation, IVT injection of a dissociated cell suspension has only been successful in rodent eyes with proportionately larger lenses and smaller vitreous cavities. The narrow vitreous cavity facilitates sufficient spread and contact for donor cells to the host retina. In larger animal models with ocular anatomy more similar to humans, the injected cells are more likely to be trapped in the vitreous far from the retina and aggregate [246]. Thus, in larger animal models, transplantation into the inner retina may benefit from using biocompatible cellular scaffolds, which can sequester the donor neurons near the target engraftment site [247]. Epiretinal transplantation of RGCs on a biocompatible scaffold may also be beneficial to abrogate the mechanical stresses imposed by intravitreally injecting a dissociated cell suspension. Several types of scaffolds have been developed, such as natural tissue scaffolds, alloplastic synthetic scaffolds, and biodegradable electrospun scaffolds for donor RGC transplantation [246–250]. In general, a scaffold should act as a supportive microenvironment to improve the survival of RGCs, direct the growth of axons radially and towards the optic nerve head, be optically transparent, and cause no impediment to the functional electrophysiological properties of transplanted cells. The delivery of a scaffold will likely require a vitrectomy and the development of new surgical methods, including techniques for fixating the scaffold in place.

Lastly, sub-ILM injection has been developed as an attractive alternative to viral vector delivery that bypasses the ILM [251]. While sub-ILM delivery would allow donor RGCs to be sequestered within microns of their final positions in a nutrient-rich tissue, like SR injection, it would require extensive lateral migration of RGCs to subserve the peripheral retina. Moreover, this method has yet to be successful for cell delivery despite numerous attempts in large animals, and it is incompatible with small rodents.

### Barriers to graft survival and integration

The success of donor RGC transplantation in the retina is challenging due to several additional factors, including the inherent stress that donor cells experience during preparation and transplantation. RGCs, like most neurons, are inherently fragile cells, and a significant source of stress includes suboptimal nutrient support, oxidative stress, and pH changes in their culture media during short-term storage while waiting for the transplantation procedure. To dissociate cells for transplantation, they must be removed from their extracellular matrix, which results in anoikis [252], leading to decreased donor cell viability. The process of dissociation can also lead to membrane rupture, as neurites are forcibly broken from the cell soma [253]. Additionally, donor cells experience shear stress during transplantation due to the physical forces involved in injecting them into the vitreous cavity. Moreover, donor cells must also face a vitreous cavity microenvironment relatively devoid of oxygenation and metabolic support once transplanted until they can migrate into the retina. This lack of support can further increase donor cell stress and decrease survival rates.

One potential solution to address these issues is to embed donor cells in hydrogels, which can provide mechanical support and buffer metabolic stress during transplantation. Hydrogels are water-based materials that mimic the retina’s extracellular matrix and provide an environment conducive to cell survival and migration. Several studies have demonstrated the benefits of hydrogels in enhancing donor cell survival and integration in the retina [248, 249, 254]. Utilizing other 3D culture-based strategies, such as microtopographic or Matrigel scaffolds, may help to improve donor neuron viability [255, 256]. Another potential strategy to improve donor cell survival is hypoxic preconditioning which can enhance the survival and integration of donor cells in multiple tissues and improve their functional outcomes [257]. Hypoxic preconditioning involves subjecting donor cells to a low-oxygen environment before transplantation, increasing their resistance to stress and improving their survival rates in the retina.

### Glia and immune system responses

The pathogenesis of optic neuropathies often involves the failure to resolve inflammation, either as a primary (e.g., optic neuritis) or secondary feature. Understanding the inflammatory microenvironment of animal optic neuropathy models and how they relate to the immunological responses of human patients is of great importance. Neuroinflammation is driven primarily by the inflammatory reactions of macroglia (e.g., astrocytes and MG) and immune cells (e.g., resident microglia and macrophages) and results in complex alterations in gene expression, morphology, and function in those cells. While numerous heterogeneous populations of ‘reactive’ astrocytes [258, 259] and microglia [260, 261] are defined at the transcriptomic level, very few are further defined at the functional level. One example that highlights interactions between immune cells and astrocytes, which, once activated, through tumor necrosis factor (TNF), interleukin 1 alpha (IL-1α), and complement component 1q (C1q) signals from myeloid cells, can induce an inflammatory and neurotoxic response in the CNS [262–265]. Genetic deletion of *Tnf*, *Il1a*, and *C1qa* prevents induction of this reactive astrocytic sub-state and ameliorates RGC death in both acute optic nerve crush [264, 266] and chronic glaucoma models [222, 267]. Importantly, blocking these neurotoxic functions preserves RGC numbers and maintains some electrophysiological functions [266, 268]. An update to this model highlights that *Fatty Acid Elongase 1* can be genetically deleted only from astrocytes to preserve RGC numbers while maintaining global reactivity intact [269] – suggesting reactivity itself is not ‘bad’ per se but that specific reactive astrocyte functions can be detrimental to RGC health. However, astrocyte responses to neurodegeneration may also be protective, as evidenced by their ability to promote neuronal survival and repair by upregulating neurotrophic factor production in murine glaucoma [264] or by providing a scaffold for axon regeneration following spinal cord injury [270].

Although different glia may share immunological functions, there are instances where cell-specific interactions are particularly important. Deciphering the specific microglial and macrophage interactions activated at various stages of the disease is vital when interpreting RGC transplant outcomes in models of optic neuropathy [271]. The formation of reactive astrocytes and microglia is a response to multiple inflammatory signalss, including the TNF, nuclear factor kappa-light-chain-enhancer of activated B cells, tenascin-C, and Toll-like receptor signaling pathways [272]. Despite early adaptive and protective features of astrocyte and microglia responses, such as the modulation of the microenvironment of individual neurons, maintenance of tissue integrity, immunomodulation, ion homeostasis, and uptake of neurotransmitters [273–275], prolonged reactivity of resident glia can also create a toxic microenvironment to RGCs and their axons [276–278]. A contributor to these interactions is the profusion of anti- and pro-inflammatory cytokines, reactive oxygen species, and toxic lipids, which have also been reported in clinical and experimental glaucoma models [279, 280]. The corresponding responses of astrocytes and microglia to glaucoma-related stress or neuron injury are critical in propagating neuroinflammation and neuroprotection; however, these interactions differ depending on the disease stage and genetic predisposition of individuals, culminating in varying chronic neurodegenerative outcomes. Controlling these neuroinflammatory mediators that drive endogenous RGC loss will be essential for cell replacement therapies because they could put repopulated neurons at risk.

Adaptive immunogenicity of donor RGCs may be a critical characteristic that threatens their survival following transplantation, particularly in clinical applications. When the immune system identifies non-autologous cells, alloreactive cytotoxic CD8^+^ T cells are activated through binding to the major histocompatibility complex class I and T cell receptors, especially in the presence of non-matching major histocompatibility complex (human leukocyte antigen (HLA) in humans) [281, 282], which leads to graft rejection. Transplantation efforts in experimental settings will require developing immunosuppression techniques that target these pathways in RGC transplantation efforts. As immune responses are complicated and event-dependent, traditional immunosuppression is often insufficient; therefore, genetic editing of HLA genes in induced pluripotent stem cells has been proposed to minimize T-cell and natural killer cell activity [283, 284]. An alternative to genetic editing is to use stem cells derived from a patient’s blood, skin, or urine samples (i.e., iPSCs) for autologous transplantation. Autologous transplantation of iPSC-derived neurons has already been shown to elicit a minimal immune response in the brain [285]. However, an advantage of allogeneic treatments is that many doses can be manufactured simultaneously from a single batch of iPSCs. In contrast, obtaining and validating iPSCs or banked HLA-matched donor cells for each patient would be expensive, time-consuming, burdensome from a regulatory perspective, and challenging to produce at scale [286, 287].

Despite efforts to limit the immune response to donor RGC delivery, it is not possible to put a needle into the eye without causing some inflammation. Therefore, an anti-inflammatory regime will likely be required, irrespective of the RGC repopulation strategy. Nevertheless, inflammation may also promote axonal regrowth [230, 288]. Inflammation stimulates the recruitment of macrophages into the retina to express pro-regenerative secretion factors and promote axon regrowth via paracrine signaling [230]. Consequently, while inflammation may negatively affect donor RGC grafting, macrophage-centered strategies may also be a promising approach to promote donor RGC regeneration.

### Visualizing and evaluating donor cells in the host retina

The success of cell replacement therapy relies on both structural and functional integration of the transplanted cells. Relying solely on endpoint assessment after enucleating the eye as a primary criterion to quantify transplantation outcomes will undoubtedly increase the resources needed to develop cell replacement therapies. Furthermore, endpoint evaluation is incompatible with human trials, so implementing non-invasive strategies to assess donor cell integration longitudinally in vivo is necessary. Early development of innovative functional and morphological readouts will allow investigators to avoid dead ends and improve the potential for successful translation into the clinic. It is also crucial to distinguish between host and donor cells to understand mechanisms of action, such as trophic support, material transfer, or *bona fide* cell replacement – this assessment can only be performed at the single-cell level.

The eye provides an advantageous setting to combine cell transplantation, molecular sensors [289, 290], electrophysiology [291–293], and advanced in vivo imaging techniques [290, 294–296] for the evaluation of functional engraftment. The intraretinal position of donor cells in the x-, y-, and z-axis needs to be defined to study donor cell distribution and structural integration following transplantation. In vivo imaging techniques, such as scanning laser ophthalmoscopy (SLO) and fundus ophthalmoscopy, may resolve the topographic positions of donor cells, provided there is sufficient contrast between donor and host cells [297, 298]. In preclinical studies, specificity has been achieved by genetically modifying the donor cells to express fluorescent reporter proteins. These proteins can be imaged with customized fundus cameras or at the cellular scale with SLO to evaluate neurites extending from donor cells [298].

Newer techniques, such as 3D optical coherence tomography (OCT), two-photon microscopy, and SLO with adaptive optics (AO), can provide depth resolution in the z-axis [299]. Volumetric information is difficult to obtain from fluorescence imaging in patients and non-human primates due to the low numerical aperture of the primate eye. Conversely, in rodents, the higher numerical aperture makes some depth discrimination possible [300], but superior depth resolution is provided by OCT, albeit at the expense of lateral resolution. Generating contrast between donor cells and the host environment in OCT is more challenging. To improve this contrast, it may be possible to genetically alter the transplanted cells to overproduce lipids or self-generate air bubbles, or to load cells with lipid-encapsulated gold nanorods or nanowires [301–304]. Pairing adaptive optics with OCT has recently opened the door to both exquisite lateral and axial resolution in normal retinas, with the contrast arising from natural organelle motility inside cells. With any contrast-enhancing approach, special care and consideration must be given to determine the possibility of material transfer that would result in the mischaracterization of host and donor cells [32] and to ensure the risk of inflammation or foreign body response is minimized [305, 306].

Despite potential mistargeted viral-based expression in RGC transplantation [32, 307], to date, no evidence for neuronal mislabeling via intercellular material transfer in RGC transplantation has been identified. However, material transfer has occurred between donor cell reporters and host cells, resulting in the misidentification of cell origin in previous photoreceptor transplantation studies [32, 308]. Material transfer can potentially occur in any cell transplantation study, including RGC transplantation. As a result, researchers must accurately characterize and label their cells to ensure clarity and interpretation of their experimental results [32]. Implementing rigorous quality control measures throughout the transplantation process is critical to ensure that the transplanted cells are accurately labeled and that any mislabeling due to intercellular material transfer is minimized. Additionally, investigators should validate their experimental results using multiple complementary methods to confirm the accuracy of their cell origin determination.

Functional integration may be assessed using a combination of in vivo and ex vivo electrophysiology techniques, such as ERGs, whole mount explants on multi-electrode arrays [309, 310], implanted electronic meshes [291], and the use of genetically encoded calcium indicators in combination with advanced imaging techniques [311]. As a non-invasive measurement of retinal electrophysiology, ERG is currently unable to resolve single-cell activity nor distinguish between host and donor RGCs [312]. On the other hand, high-density multi-electrode arrays can quantify single-cell activity, but assigning individual electrodes to specific cells can be challenging, and recording from the retina is limited to a single endpoint [309, 310]. Unlike MEAs, implanted electronic meshes can record from single neurons chronically in the retina of live mice, but such systems have yet to be used to study transplanted RGCs and are limited in scale and number of recording electrodes [291]. Genetically encoded calcium indicators have shown the most utility in cell transplantation experiments and provide scale to study hundreds to thousands of single donor and host RGCs simultaneously [311, 313]. Still, their use has received mixed acceptance. These approaches require expensive and highly customized imaging systems because visible light stimulates photoreceptors and confounds the ability to image the fluorescent signal from the calcium indicators. Consequently, there is a demand for label-free approaches to measure donor RGC function in the retina. A promising label-free strategy to image RGC function in humans has recently been developed based on full-field swept-source OCT technology, but this approach cannot yet provide cellular scale resolution [314].

Irrespective of the approach, the signal-to-noise ratio remains a significant hurdle for parsing the functional integration of donor cells from endogenous neural activity. Implementing a neuronal activity control mechanism, such as Designer Receptors Exclusively Activated by Designer Drugs (DREADDs) or optogenetic techniques that allow researchers to control the activity of specific neurons using small molecules and light, respectively, may be useful for distinguishing between donor and host RGCs [8, 315, 316].

Many of the same parameters and instruments used to study the retina in animals (especially large animals) may be used in human trials [317]. However, unlike preclinical research, where transgenic expression of fluorescent or other proteins can facilitate graft visualization, engineering of cell products specifically for optical monitoring is unlikely to be advisable in clinical trials. Recent developments in adaptive optics OCT have enabled label-free visualization of single ganglion cell somas and single nerve fiber bundles [294], and so this technology will likely constitute the primary structural outcome in human patients. While the availability of AO-OCT instruments is currently limited to a few specialized labs, ultimately, this technology should be applied to preclinical RGC transplantation experiments, ideally in conjunction with the detection of fluorescent reporter tags in SLO or AO-SLO to advance our understanding of how to interpret these label-free techniques until OCT contrast agents are further developed. Lastly, in addition to studying transplanted RGCs, in vivo imaging techniques may be applied to analyze and better understand the mechanisms of retinal neuron degeneration in glaucoma and other optic neuropathies. This will enable the development of models that better recapitulate human disease and our understanding of how the host retina is affected at the cellular scale by the various intervention strategies currently under development.

### Future directions for transplantation methods and models

Transplantation research has made significant strides in recent years, but many areas still require further development. An important future direction for RGC transplantation methods and models involves further developing and characterizing models that accurately mimic the anatomical features of the human eye, such as the macula and the collagenous lamina cribrosa, and that pathophysiologic changes that occur in these structures in disease. Additionally, more emphasis must be placed on creating models for studying neuronal transplantation in specific pathological contexts, such as normal-tension glaucoma, and models that do not induce active neurodegeneration, such as knockout models of *Brn3b* (*Pou4f2*), *Brn3a* (*Pou4f1*), and *Math5* (*Atoh7*). Exploring autoimmune disease models, including multiple sclerosis, may also improve clinical translatability to various pathological targets. Finally, larger animal models, such as primates and pigs, may be better positioned than rodent models to develop clinically relevant transplantation techniques, including pars plana vitrectomy, ILM peeling, and implantation of semi-rigid scaffolds. Therefore, focused efforts in transplanting RGCs in these models will likely yield significant progress in the field.

Ongoing development of preclinical optic neuropathy models should prioritize those that can better mimic the natural progression of human disease and demonstrate the effect of aging on the survival and integration of donor cells in the retina. While it may be easier experimentally to attain RGC engraftment in early disease stages, clinical translation necessitates success in late-stage disease models. Enhancing engraftment in advanced optic neuropathy may necessitate immunomodulatory approaches to reduce inflammation and scarring. Additionally, extracellular matrix molecules, hydrogels, or biocompatible scaffolds should be rigorously evaluated for their potential to enhance cell survival and integration in various injury models.

In addition to evaluating the optimal source and developmental stage for donor RGCs, additional focus on cell dosage and composition with non-RGC support cells would be valuable. Such studies might investigate the optimal ratios of different cell types on donor RGC engraftment. Graft rejection and immune responses remain significant challenges for successful transplantation. However, this may represent a more formidable obstacle for experimental xenografts than eventual clinical trials of autologous or allogenic cell sources. Nonetheless, it will be essential to examine the immunogenicity of donor RGCs in clinically relevant models and optimize retinal-specific immunosuppression techniques that target alloreactive T cells or engineer donor RGCs that evade immune surveillance.

Ongoing research must implement rigorous quality control measures, including monitoring for material transfer throughout the transplantation process and validation of results using multiple complementary and standardized methods to facilitate accurate characterization and labeling of transplanted cells. Particular attention should also be given so that dead RGCs inside phagocytosing cells are not counted in successful RGC transplantation results. A combination of in vivo and ex vivo electrophysiology techniques can be used to assess functional integration. For these systems, chemogenetic or optogenetic systems can overcome the high signal-to-noise ratio for discriminating functional integration from endogenous neural activity. Lastly, improving image capabilities in larger eyes for both experimental and translational purposes will be essential, with OCT metrics being the primary structural outcome in human patients (Table 2).

**Table 2:** Future directions for transplantation methods and models (SDG2)

Research Area	Future Goals
Anatomically accurate models	Evaluate RGC transplantation in models that accurately mimic the human eye’s anatomical features, including the macula and the collagenous lamina cribrosa, to study neuronal transplantation in various pathological contexts
Disease models	Establish models for studying neuronal transplantation in different pathological contexts such as normal aging, normal-tension glaucoma, autoimmune disease, and developmental models that do not induce active neurodegeneration
Larger animal models	Prioritize larger animal models to develop clinically relevant transplantation techniques, including procedures like pars plana vitrectomy, internal limiting membrane (ILM) peeling, and implantation of rigid scaffolds
First-in-human trials	Define an “ideal” optic neuropathy patient suitable for initial clinical trials and establish an experimental model to mirror this clinical phenotype
Transplantation timing	Investigate the effect of disease progression and aging on the survival and integration of donor cells
Overcoming barriers to engraftment	Evaluate use of immunomodulatory agents and extracellular matrix modulators to promote cell survival and integration
Graft specifications	Investigate the effects of different cell doses on graft survival, integration, and functional outcomes. Explore the potential benefits and optimal ratios of transplanting a mixture of RGCs and non-RGC support cells
Immune responses	Explore methods for promoting immunotolerance of transplanted RGCs, such as immunosuppressive drugs, gene editing techniques, or extracellular matrix modulators that may improve cell survival and integration by inhibiting reactive gliosis and immune cell infiltration
Scaffolds	Explore new techniques for delivering donor RGCs to the retina, such as developing improved scaffolds or designing methods that allow for safe and efficient migration of donor cells from the epiretinal surface
Delivery methods	Evaluate and develop alternative cell delivery methods, such as sub-ILM transplantation, which may offer better donor cell survival and integration outcomes
Preconditioning techniques	Investigate diverse preconditioning methods to improve donor cell resistance to hypoxia, para-inflammation dysregulation, and oxidative stress
Quality control and validation	Implement quality control measures throughout the transplantation process and validate results using multiple complementary and standardized methods to facilitate accurate characterization and labeling of transplanted cells, including the possibility of material transfer
Imaging capabilities	Improve imaging for experimental and translational purposes, benchmarked to OCT metrics as the primary structural outcome in human patients

## SDG #3. RGC survival, maturation, and host interactions

Challenges in achieving engraftment and long-term survival of donor RGCs within the host mammalian retina are at least partly related to the highly organized tissue structure and interactions among the various resident cell populations. Prior work investigating mechanisms of retinal development, neurodegeneration, and neuroprotection provides numerous potential avenues for promoting long-term survival and maturation of transplanted RGCs, enhancing neurite outgrowth, and supporting appropriate donor neuron function [318], which is necessary for transplanted cells to integrate into the existing neural circuitry and contribute to visual signaling.

### RGC survival and neuroprotection

Long-term survival of transplanted RGCs is central to sustained visual improvement and is a significant limitation of most published studies. While some human cells may successfully migrate following SR or IVT injection into a non-human primate retina, the survival rate is typically below 1% [319]. Several methods might be used to improve the survival rate of transplanted cells.

RGCs are particularly vulnerable to metabolic insults and rely on mitochondrial oxidative phosphorylation for their high energy demands [320]. Therefore, ensuring adequate metabolic support for newly transplanted cells is critical for survival in the peri-transplant period when most cell death occurs. Anabolic activity and aerobic glycolysis positively correlate with cell survival in neurogenerative contexts [321]. Nicotinamide, the amide form of vitamin B3, has emerged as a potential neuroprotective agent [322, 323]. Nicotinamide aids in producing NAD^+^, a crucial coenzyme in mitochondrial respiration and cellular energy production, prevents axon degeneration (Wallerian degeneration), and improves visual function in existing glaucoma patients [324]. Supplementing the graft with nicotinamide may provide a metabolic boost that helps the cells survive the peri-transplant period.

The vitreous cavity, the site of RGC transplantation, is relatively hypoxic [325], undermining oxidative phosphorylation. This environment might further strain the metabolic machinery of the newly transplanted cells, leading to an increased likelihood of cell death. Therefore, strategies promoting metabolic homeostasis in this challenging environment are essential, and hypoxic preconditioning may be beneficial. Another approach for improving donor RGC survival may be to supplement the vitreous cavity with pyruvate [326]. Pyruvate is critical in cellular metabolism, serving as a key intersection point between anaerobic glycolysis and aerobic oxidative phosphorylation. It serves as a substrate to generate adenosine triphosphate in hypoxic conditions and, in combination with nicotinamide, resulted in significant short-term improvement in visual function in glaucoma patients [327]. Pyruvate is normally supplied to neurons by oligodendrocytes [328], so in addition to providing exogenous pyruvate, promoting metabolic coupling with oligodendrocytes may also be essential for the long-term survival of donor RGCs and should be investigated. Interventions aimed at improving the antioxidant capacity of transplanted cells may also be beneficial. Since oxidative phosphorylation can generate harmful reactive oxygen species, enhancing the ability of the cell to neutralize these compounds might help prevent cell death due to oxidative stress [329]. Efforts made to better understand the metabolic demands of donor RGCs at all stages of repopulation in pathologic environments will, therefore, provide critical insights for improving cell survival after transplantation.

While several neuroprotective strategies have been explored for promoting host RGC survival in optic neuropathy models, few have been studied in the context of RGC transplantation [330]. Neuroprotection is likely essential for repopulation approaches, as the neurodegenerative environment into which new RGCs are to be introduced is likely to challenge graft survival [331]. While slow-release neurotrophic factors can significantly increase the number of grafted donor RGCs in vivo [236], most donor RGCs do not survive transplantation even with neurotrophic support. While developing neuroprotective therapies will be vital for supporting donor RGC survival, clinical translation of these therapies may also limit the need to replace or regenerate RGCs in patients.

There has been considerable interest in directly suppressing pro-apoptotic signaling for RGC neuroprotection, and many of the same interventions may help promote donor RGC survival following transplantation. For example, Caspase 2 siRNA is currently in clinical trials to treat several optic neuropathies [332, 333]. Other avenues for engineering or blocking pro-apoptotic pathways (e.g., with PARP and RIPK inhibitors [334, 335]) could also be relevant. Targeting the genes and pathways central to RGC apoptosis may also help improve donor RGC survival [336]. Importantly, this could be achieved via pharmacological treatment or genetically engineering the donor RGCs.

BCL2-Associated X protein (BAX) is a pro-apoptotic protein critical in cell apoptosis [337]. Despite a loss of axons following an insult, RGCs that lack BAX exhibit enhanced survival in animal models [338]. Knocking out BAX in RGCs disrupts the usual apoptotic pathway, thereby preventing cell death. This approach, however, does not address the underlying disease pathologies and may not address all sources of cellular stress. Specifically, in the case of BAX knockout, RGCs become quiescent, requiring additional strategies to promote endogenous axonal regeneration [338]. Dual leucine zipper kinase (DLK) and leucine zipper kinase (LKZ) are other important mediators of RGC death [336]. Inhibition of these kinases has improved RGC survival in various injury models, including ocular hypertensive glaucoma and traumatic optic nerve injury. Pharmacologic inhibitors of DLK and LZK have the potential to slow or even halt the progression of RGC death [339]. However, while known trophic and pro-regenerative pathways may promote RGC survival and neurite outgrowth in the short term, permanent suppression of such pathways could be counterproductive, given their transient role in neuronal development. For example, while DLK is a negative regulator of RGC survival, it is a positive regulator of neurite outgrowth [340]. Therefore, a combination of therapies will likely be required to promote transplanted RGC survival and integration into the existing retinal neurocircuitry.

In addition to reestablishing lost circuits within the retina and the brain, donor RGCs might benefit the host retina by providing a neuroprotective effect to surviving endogenous neurons. For example, a recent study showed that one week after stem cell-derived RGCs were transplanted, significantly more host RGCs survived an optic nerve crush injury [341]. One possible explanation for how transplanted cells can improve host neuron survival is by transferring their extracellular vesicles that contain diverse, multifactorial cargo to the host neurons [342]. Studying these and other mechanisms by which donor RGCs confer protection to host neurons is an important area of focus, as it could signal a secondary benefit of RGC transplantation.

Taken together, the successful transplantation of RGCs into the retina requires careful consideration of several factors. Genetic enhancement of donor RGCs, optimizing of RGC metabolism early after transplantation, identifying neuroprotective therapeutic targets, and investigating the beneficial effects of donor cells on the host retina are all crucial in optimizing the success of RGC transplantation approaches.

### RGC maturation

The optimal stage of maturity for transplanted RGCs remains an important unanswered question. Fully matured donor RGCs may have a reduced ability to migrate and integrate into the retina, as seems to be the case for photoreceptors [343]. Most RGCs differentiated in vitro are relatively immature and typically do not undergo subtype specification [344]. In most RGC differentiation protocols, the maturation state of cells in culture is highly heterogeneous. Though this may be advantageous from a plasticity perspective, clearly defining and controlling the stage of maturity before transplantation will be necessary to rigorously assess this variable in transplantation outcomes. For instance, RGCs derived from day 21 mouse retinal organoids survive better than those from day 16 [54]. Thus, balancing plasticity and maturation by identifying the most suitable developmental stage for donor cells to achieve optimal outcomes is a key goal.

Interactions between donor cells and host organs are essential in guiding their differentiation and maturation [54], and such interactions may only occur following transplantation. For instance, astrocyte-RGC interactions regulate RGC maturation during development [227]. Investigating whether these interactions are also relevant to RGC transplants will be important. The retinal microenvironment may also promote the differentiation and maturation of transplanted retinal organoids [345]. While molecularly immature (RBPMS negative) RGCs survive following transplantation, there is a propensity for donor RGCs integrated into the GCL to preferentially express the mature RGC cell marker, RBPMS [236]. These findings indicate either that molecular cues in or near the GCL drive RGC maturation in vivo or that RBPMS-expressing RGCs are more likely to integrate [236]. However, the specific molecular signals that promote RGC maturation in the GCL remain unclear and may involve interactions between RGCs and neighboring cells or extracellular matrix proteins within the GCL. Further research is needed to identify these cues and determine their role in RGC maturation.

Lastly, in the early stage of retinal development, electrophysiologic activity in retinal neurons and within their postsynaptic targets, achieved through retinal waves, plays a crucial role in cell differentiation, maturation, and circuit development [346, 347]. While it is unclear the extent to which coordinated retinal activity may promote neuronal maturation or circuit development in adult or diseased retinas, methods to promote electrical activity (e.g., using optogenetic strategies or application of exogenous electrical fields) in donor RGCs may be used to investigate this potential.

### Host microenvironment preparation before transplant

In the later stages of optic neuropathies, the retina often exhibits neuroinflammation, peripheral immune cell infiltration, and host glial/immune cell reactivity. In glaucoma, these responses are commonly triggered by chronically sustained high IOP and/or the subsequent death of RGCs, which produces a neurotoxic microenvironment that may impair the acceptance of donor cells by the host retina. Consequently, IOP control will be a prerequisite for any RGC repopulation strategy for glaucoma. Even when IOP returns to homeostatic levels, neuroinflammation and CNS glial reactivity persist [348], making it potentially difficult for transplanted donor cells to survive and integrate into the host retina. To improve the success of RGC transplantation, the host microenvironment may need to be “reset,” and host immune cells, particularly phagocytic cells [349, 350], may need to be depleted or suppressed, at least temporarily. However, it has become clear that not all phagocytosing cells are harmful. Microglia and astrocytes, for example, play essential roles in developmental processes like vascularization, RGC development, and fine-tuning of neuronal circuit connectivity [351–353], as well as maintaining retinal homeostasis, including immune responses, metabolism, neuronal activities, and phagocytosis [354, 355]. Nevertheless, methods to drive a protective phenotype specifically and reliably in both microglia and astrocytes remain in development.

Studies in mice raised without microflora in a germ-free environment do not exhibit significant RGC death following elevated IOP [356]. This finding indicates that the peripheral immune system and the microbiome may play an important role in glaucoma progression and may have a detrimental effect on donor RGC survival [353]. Moreover, the role of the microbiome in the immune and nervous systems in zebrafish has recently been described to affect regeneration [357]. Therefore, it may be informative to perform RGC transplantation in animals raised in a germ-free environment and assess donor RGC survival.

Lastly, preparing a supportive and nourishing host retinal microenvironment may be necessary to support donor RGC transplantation. A combination of factors may be required to establish this environment, including growth factors, anti-inflammatory agents, antioxidants, and other molecules that promote cell survival and integration. Developing a better understanding of the mechanisms involved in glaucoma and the role of the immune system and glial cells in disease progression will be critical for advancing the field of RGC transplantation and ultimately improving the treatment of glaucoma.

### Host microenvironment regulation after transplant

Transplant studies have primarily focused on allogeneic or xenogeneic donor cells, which pose an exceptionally high risk of rejection. In some ways, this represents more of an experimental hurdle than a translational obstacle since allogeneic or autologous transplants into humans are likely subject to greater acceptance than xenografts. Nonetheless, immunosuppressive regimens used for preclinical animal studies are highly variable, and optimal immunosuppression approaches for RGC transplantation remain unclear. Transplantation into immunodeficient animals (Nod/SCID mice or athymic nude rats) may enable researchers to circumvent these experimental hurdles at early stages, but eventual studies in large animals will require optimization of immunosuppressive approaches. Moreover, while immunosuppression in patients receiving autologous transplantation may not be necessary to prevent outright graft rejection, in late-stage glaucoma, immune cells are already highly active in the retina [358], which will necessitate immunotherapy.

Beyond the adaptive immune system, previous studies suggest that donor cell integration in the retina is enhanced when reactive glia responses in the retina are genetically or pharmacologically suppressed [241, 359], indicating a critical role for reactive astrocytes in neural graft integration [360]. Despite this, there remains an unmet need to develop a reliable protocol for regulating astrocyte and microglia reactions after transplantation to achieve longer-term survival and robust integration. Glial-related disease development can vary between sexes in some disease models. For example, retrospective studies suggest that girls with NF1-associated OPGs restricted to the optic nerves are more likely to lose vision and require treatment than their male counterparts [190, 361]. Similarly, in a genetically engineered mouse model of NF1-associated OPG, a sex-specific effect operates at the level of non-neoplastic glia, where the elaboration of neurotoxic molecules in response to estrogen underlies the observed increase in RGC loss, nerve fiber layer thinning, and visual acuity reduction in female mice [190, 193]. Such variation in glial reactivity highlights the importance of individual and disease-specific immunoregulation in post-transplant management and suggests that clinical translation must carefully consider these factors.

One approach to mitigating innate immune responses to transplanted cells involves the masking of externalized phosphatidylcholine on the plasma membrane, which serves as an “eat-me” signal on donor cells, using Annexin V. This approach reduces the recruitment of microglia to the delivery site [362]. However, this pretreatment has limitations, as it does not provide continuous protection to the donor cells. Therefore, a more comprehensive regimen that addresses post-transplant immune regulation may be necessary. This immunosuppressive regimen could involve a combination of strategies, such as blocking multiple signals or using a mixture of immunosuppressive drugs.

Lastly, by exploring single-cell transcriptome data of the developing human retina, various receptor-ligand candidates have been identified to control donor RGCs in vivo [236]. Establishing an exogenous chemokine gradient across the retina improves the structural integration of donor RGCs through guided migration [236]. Consequently, using transcriptomic data from the adult, diseased, and developing human retina could be a powerful approach for identifying targets to engineer the retinal microenvironment and individual RGCs and control various cellular processes (e.g., synapse formation, phagocytosis, axon growth, etc.).

### Future directions for RGC survival, maturation and host interactions

Fortunately, great efforts have been spent identifying the molecular mechanisms underlying RGC death and dysfunction in the context of pathological states, which have yielded numerous neuroprotective approaches to enhancing the survival of endogenous RGCs. The RGC transplantation field is poised to benefit from this rigorous prior research by testing, alone and in combination, many of these pharmacologic, genetic, and microenvironmental interventions, which will hopefully enhance donor RGC survival to a rate needed to achieve functional benefits in optic neuropathy.

Sophisticated 3D retinal tissue culture models have been increasingly important in understanding RGC development and disease pathology. However, further development of these tissue-engineered models to enable longer-term neuronal viability and function and to better mimic the in vivo environment is still necessary. These models might then be used as stand-ins to investigate RGC transplantation without the confounding effects of other systems, such as the immune system. Furthermore, by studying the interactions between RGCs and neighboring cells in these well-defined systems, researchers may be able to identify the specific molecular mechanisms involved in RGC survival and maturation, ultimately improving transplantation outcomes. Separately, developing systems that model the complex interplay between the immune system and RGC degeneration, and developing immune-based therapies to prevent donor RGCs from being collateral damage in a hostile disease environment, are of great interest.

Lastly, recent advances in single-cell sequencing technology have provided new tools for investigating the molecular cues involved in RGC survival and maturation. By analyzing the gene, protein, epigenetic, and metabolic expression profiles of individual RGCs at different stages of development and engraftment following transplantation, investigators may be able to identify the specific molecular pathways involved in promoting the function of successful RGC transplants (Table 3).

**Table 3:** Future directions for RGC survival, maturation and host interactions (SDG3)

Research Area	Future Goals
Neuroprotective approaches	Explore the efficacy of neuroprotective approaches, including those initially developed to prevent endogenous RGC death, in the context of RGC transplantation
Epigenetics	Identify specific epigenetic mechanisms that regulate RGC development and survival and develop epigenetic therapies that can be applied to augment donor RGC transplantation
Donor cell maturation	Determine the most appropriate developmental stage and timing for donor RGC harvesting to achieve the best possible transplantation outcomes
Tissue-engineered retina models	Develop more advanced tissue-engineered retina models to provide longer-term neuronal health and better mimic the in vivo environment. These models could then be used to investigate RGC transplantation without the confounding effects of peripheral immunity
Role of the immune system	Better understand the complex interplay between the neuroinflammation and RGC degeneration and develop immune-based therapies to prevent donor RGCs from being collateral damage in a hostile disease environment
Role of CNS resident glial cells	Achieve comprehensive understanding of interactions between astrocytes and Müller glia with immune cells (resident microglia and peripheral macrophages) in maintaining retinal health, preserving RGC viability during disease/trauma, and promoting regeneration and transplant integration
Imaging techniques	Develop new tools and methods for high-resolution imaging and quantifying donor RGC survival and axonal regeneration in vivo to allow for time-course studies
Single-cell sequencing technology	Use the advancements in single-cell sequencing technology to investigate the molecular cues involved in RGC survival, maturation, and functional engraftment. Analyze the gene, protein, and metabolic expression profiles of individual RGCs at different stages of development and engraftment after transplantation

## SDG #4. Inner retinal wiring

**Fig. 3:**
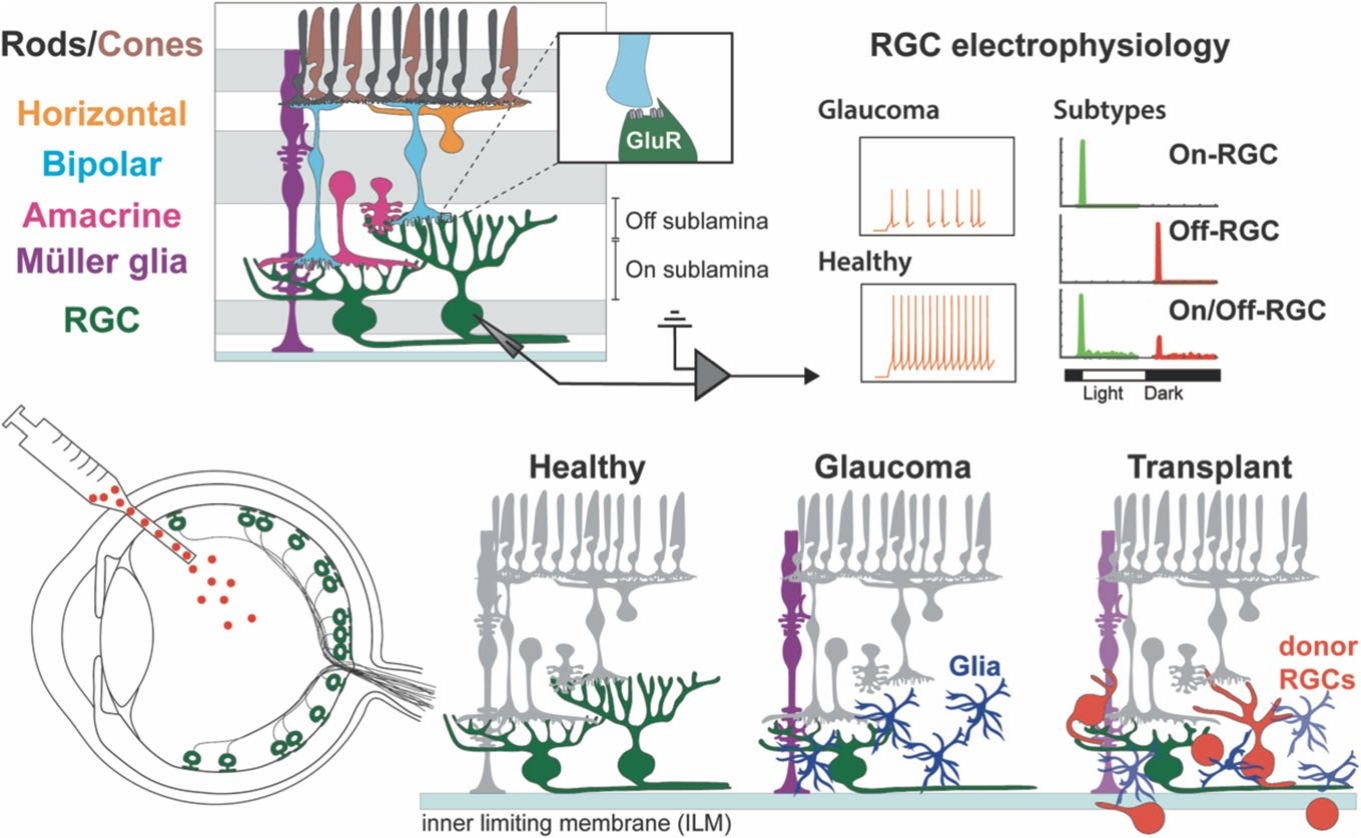
RGC neurocircuitries in healthy, diseased, and transplanted retinas. Bipolar and amacrine cells establish direct contact with RGCs to relay visual information. Different RGC subtypes extend their dendrites into ON and OFF sublamina in the inner plexiform layer and exhibit different electrophysiological responses. Glaucoma causes dendrite retraction and eventual death of RGCs and the activation of astrocytes, microglia, and Müller glia, while photoreceptor, bipolar, amacrine, and horizontal cells are relatively unaffected. RGC transplantation must replace lost RGCs, return the diseased retina to a homeostatic state, and establish neurocircuitry between host and donor cells. While donor RGCs have been shown to survive in the retina, few are currently able to migrate into the ganglion cell layer, with the inner limiting membrane (ILM) serving as a major barrier for intravitreal (IVT) delivery, and even fewer form de novo neurocircuits in the retina

A sound understanding of inner retinal wiring and the ability to manipulate it are central to the anatomical and, perhaps more importantly, the functional success of cellular approaches for retinal regeneration. While our knowledge of this complex process has significantly advanced in recent years (Fig. 3), there are still unmet research needs in this area, summarized as the following: (i) the need to define “success” and intervention endpoints, (ii) how best to maintain dendritic integrity, (iii) the importance of glial and innate immune responses on the inner retinal circuitry, and (iv) when it may be best to intervene during the disease process.

### Studying donor RGC polarization, neurite outgrowth and characteristic electrophysiological properties

Research on stem cell-derived RGCs must ensure that the cells can polarize with dendritic and axonal compartments localizing to the correct retinal layers and that cells can form functional synapses while exhibiting characteristic electrophysiological properties. However, the ability to study these processes in vitro is limited, and models must be suitable for high-throughput experimentation.

RGCs in retinal organoids elaborate neurites but do not recapitulate normal retinal circuitries, have no efferent connectivity, and die over time, making RGC polarization, neurite outgrowth, and synaptogenesis challenging to study in these systems [363]. Moreover, organoids lack a fovea and do not faithfully mimic each retinal layer. Nevertheless, these neurons can form de novo synapses after being dissociated [364], providing evidence for synaptic plasticity in stem cell-derived RGCs, which is essential for restoring visual circuits. In addition, in vitro assembloid models have been established to mimic retinofugal projections from RGCs to postsynaptic targets in the brain [19]. Demonstrating the electrophysical properties of RGCs in vitro is essential, as a lack of success in vitro may indicate comparable failure in an in vivo environment, with the caveat that degenerating cultured retinas may be less capable of synaptogenesis. In vitro systems lack functional retinal neurocircuitry, and it is unclear how cell culture conditions impact the cells’ electrophysiological response. For example, an array of culture conditions have various pros and cons for electrophysiology experiments in 2D and 3D neuron cultures [365].

### Intrinsic and extrinsic factors for successful RGC integration

Recapitulating the RGC connectivity to retinal circuits that occurs developmentally is likely critical to achieving functional therapeutic RGC transplantation. Donor RGCs must extend their dendrites into the IPL with appropriate glutamate receptor expression and specificity for the inner (ON) or outer (OFF) IPL sublamina. However, it remains to be seen whether donor RGCs will do this spontaneously based on environmental cues or will require some additional molecular factors, either inducible cell-intrinsic or extrinsic cues, to be delivered to the IPL.

Several aspects of inner retinal development are pertinent to understanding the signaling factors that may facilitate donor RGC engraftment within the IPL. For instance, semaphorin-plexin interactions contribute to the patterning and stratification of RGC dendrites in different sublayers of the IPL [366]. Insulin and mammalian target of rapamycin (mTOR) signaling pathways can also promote RGC dendrite regeneration and synapse reassembly in the IPL following axonal injury [367].

In addition to molecular cues, retinal cells, including starburst amacrine cells and bipolar cells, themselves play significant roles in pre-patterning the IPL. Starburst amacrine cells help establish the basic structure of the IPL during development [368]. They are among the first cells to stratify within the IPL, effectively acting as a scaffold for the growth and development of other cells, including RGCs. Starburst amacrine cells also play a crucial role in direction selectivity, a fundamental aspect of visual processing that allows RGCs to respond preferentially to motion in specific directions. Interestingly, in addition to the amacrine cells in the IPL, displaced RGCs can also be found within the IPL, but in orders of magnitude less than in the GCL [294, 369, 370]. Little is currently known about displaced RGCs, but given that they are evolutionally conserved, they must serve some crucial roles in the retina [371]. Bipolar cells help to convey information from the photoreceptors (rods and cones) to RGCs. In the context of IPL patterning, bipolar cells also contribute to the formation of synaptic connections within distinct IPL sublamina [372, 373]. The stratification of bipolar cell axon terminals within the IPL is thought to influence RGC dendritic development and their ultimate stratification within the IPL. It is fortuitous that afferent inner retinal neurons spontaneously prepattern the IPL during development and that these cell populations are relatively unaffected by optic neuropathies since this suggests that, even in advanced optic neuropathy, there should be an IPL scaffold into which donor RGCs may be able to integrate. Understanding the molecular cues that may be common across all RGC subtypes during development to promote dendritic extension would be very useful.

Cell-intrinsic factors related to RGC survival and axon regeneration have been studied in mouse models following injury. Molecular targets, such as mTOR, phosphatase and tensin homolog (PTEN), BDNF, nerve growth factor, ciliary neurotrophic factor (CNTF), insulin, and Kruppel-like factors (KLF), have been identified as critical for RGC survival and regeneration [374–377]. However, the relevant molecules at each step of retinal circuit restoration remain unclear, and further research is needed to understand the functional role of trophic factors and guidance molecules in IPL regeneration. Therefore, a comprehensive, systematic characterization of developing, degenerating, and transplanted RGCs at multiple levels, including transcriptomic (scRNAseq and spatial transcriptomics), structural (STORM and nanoscopic imaging to visualize the dendritic arbors and synapse arrangements), and functional techniques (electrophysiology) will be required. Using human tissue in addition to animal studies will increase the utility of these investigations.

Extrinsic factors in the ocular microenvironment also influence RGC integration into retinal circuitry. For example, previous work has shown improved donor RGC integration in retinal damage models compared with healthy wild-type retinas [54], consistent with photoreceptor transplantation studies [378]. However, it remains to be determined if donor RGCs can better integrate into these retinas because there is more available physical space or other unknown mechanisms.

As previously mentioned, the ILM is a known physical barrier to transplanted RGCs [242, 243], but its role in donor RGC integration is still being determined. ILM recognition appears essential to proper RGC lamination and polarity during development [379]. Molecular factors, such as integrins expressed by RGCs and laminins associated with the ILM, can affect RGC integration by providing molecular cues to guide neurite outgrowth [236]. Further investigation is needed to determine how the ILM, other retinal cells (e.g., amacrine cells, bipolar cells, and MG), and the IPL extracellular matrix govern donor RGC integration at the molecular level.

### Maintaining dendritic integrity

The importance of dendritic integrity in optic neuropathy disease models has been established through the observation that loss of complexity in RGC dendritic trees occurs before axonal loss [147, 150, 154]. This phenomenon is particularly evident in OFF-transient RGCs, which exhibit a rapid decline in both structural and functional organization upon IOP elevation [147, 154]. Indeed, some RGC subtypes undergo significant dendritic rearrangements as early as seven days after induction of elevated IOP [150, 162]. Interestingly, early dendritic remodeling may be linked to axonal regeneration [380]. Furthermore, there is evidence of circuit plasticity ocurring after IOP elevation, with the rewiring of developmental presynaptic bipolar and amacrine cell partners with a resilient RGC type in the inner retina [381, 382].

Given that dendritic resprouting and synaptogenesis can be promoted in injured RGCs, it is plausible that these processes could be induced in newly repopulated RGCs. In fact, changes in spontaneous activity and light-evoked responses in endogenous injured RGCs are noted before any detectable dendritic loss, pointing towards a potential relationship between dendritic remodeling and functional changes in the cells. However, outstanding questions remain regarding the extent and reversibility of dendritic tree remodeling in the context of injury. For instance, while OFF-sustained RGCs show perturbed light-evoked responses following injury, their dendritic structure remains intact [154]. ON-transient and ON-sustained RGCs also demonstrate normal functional receptive field sizes following injury, but their spontaneous and light-evoked firing rates are reduced [154]. How these different responses relate to the extent and reversibility of dendritic remodeling or the propensity of these RGC subtypes to generate new dendrites after introduction into the diseased retina remains to be determined. Further research will be needed to explore these pathways and the potential for reversing the damage caused by IOP elevation or other insults.

To address these questions, investigations of dendritic and synaptic integrity in various models of optic neuropathy will be necessary. Techniques such as biolistic labeling and rigorous approaches for determining the co-localization of pre- and post-synaptic markers may be useful [381]. Such methods should rely not only on the fluorescent overlap but also on techniques with greater specificity, such as fluorescent protein reconstitution across synaptic partners [383]. Furthermore, much remains to be understood regarding the molecular mechanisms underlying dendritic remodeling, including the role of intracellular signaling pathways and gene expression changes. In particular, much of what is known about dendritic remodeling may be heavily biased towards only a few RGC subtypes because of the availability and use of specific transgenic mice for these studies, such as the Thy1-YFP line (B6.Cg.Tg(Thy1-YFP)HJrs/J), which primarily labels alpha-RGCs [142]. Moreover, RGCs distributed spatially across the retina vary with respect to arborization, irrespective of the RGC subtype [384]. Therefore, it may be necessary to drive dendritic remodeling in repopulated RGCs according to both their specific subtype and spatial distribution across the retina.

### Glial factors and innate immunity

Optic neuropathies and RGC death can trigger changes in retinal cells beyond RGCs [292]. MG and innate immune responses are likely crucial factors influencing donor RGC integration. MG are critical in retinal homeostasis and regeneration [385]. However, the re-entry of reactive MG to the cell cycle leads to proliferation and the formation of a glial scar [27, 385]. These scars can act as reservoirs for accumulating extracellular matrix proteins, including chondroitin sulfate proteoglycans [386], which can hinder neurite extension by transplanted cells. Treatment with chondroitinase ABC digests chondroitin sulfate proteoglycans and enhances donor cell migration, neurite outgrowth, and synaptogenesis in the retina [387–390]. Future efforts may explore using chondroitinase ABC or other methods of modulating the extracellular matrix to improve donor RGC integration into the retina.

Innate immune responses, including microglial reactivity and infiltration of peripheral immune cells, can also influence the integration of transplanted cells [391]. While microglia can play a beneficial role in clearing debris, promoting tissue repair, and pruning and revising dendritic arbors and synaptic connections in the retina during development and disease, their chronic activation can lead to neuroinflammation and exacerbate retinal damage either directly or through promoting additional reactivity response by astrocytes [392, 393].

Modifying the transplanted cells or host environment to avoid or modulate MG, astrocyte, microglial, or other immune responses may be necessary to enhance dendritic integration within the IPL. As discussed, masking the “eat me” signal through preconditioning donor RGCs with annexin V can improve the survival of donor RGCs after xenotransplantation by preventing microglia from phagocytosing donor cells [362] and may also protect immature neurites that would be subject to pruning. Alternatively, changing the environment in which RGCs are transplanted may prevent or modulate these responses. For example, PLX-mediated microglia ablation might improve donor RGC engraftment. However, the potential unforeseen consequences of such modifications must be considered. Microglia ablation could elicit monocyte infiltration to fill the void, but these cells may not function the same way [394]. Glia are essential in regulating the environment around neurons and contribute to synaptic plasticity [354, 395, 396]. Further, depletion has also been related to neurodegenerative changes [397]. Thus, understanding the interactions between glia and retinal neurons could inform methods of enhancing donor RGC integration within the inner retina.

### Future directions for inner retinal wiring

Our ability to promote and control RGC integration into the inner retina will be enhanced by a better understanding of the mechanisms by which the inner retina processes visual information. Recent advances in imaging technologies, such as two-photon and light-sheet microscopy, have allowed researchers to visualize the electrophysiological activity of large populations of retinal neurons in real time [398]. These techniques can be used to study the dynamics of retinal circuits and how they respond to changes in visual stimuli. Moreover, it enables the classification of different retinal cell types and allows for high-resolution measurements of calcium entry at synaptic release sites across multiple bipolar cells simultaneously [398]. Applying these functional imaging techniques to RGC transplantation will likely yield important information about their spontaneous functional engraftment capability and provide an essential tool for assessing methods to augment their integration into retinal circuits.

Moreover, optogenetic advancements have uncovered new avenues for studying inner retinal wiring. Optogenetics can selectively activate or inhibit specific cell types in the retina, enabling researchers to probe the function of individual neurons and their interactions with other cells. For example, activating all amacrine cells through optogenetic stimulation promotes the recovery of both ON and OFF responses in the retina [399–401]. This method also facilitates studying diverse forms of retinal processing, including sustained and transient responses. Consequently, utilizing optogenetics to modulate specific inner retinal circuits may be useful for studying and/or enhancing donor RGC connectivity within recipient retinas.

Finally, consideration of the goalposts needed to successfully demonstrate the inner retinal wiring of transplanted donor RGCs is warranted. At the minimum, donor RGCs should extend dendrites into the IPL and express functional glutamate receptors. At the other end of the spectrum, we desire synaptic connectivity and function of transplanted RGCs that is indistinguishable from wild-type healthy retina. Goalposts to reach in between these two ends of the spectrum include donor RGCs that exhibit dendritic targeting to the correct sublamina, donor RGCs that show ON vs. OFF responses and generate sustained vs. transient responses, and donor RGCs that have proper connectivity with bipolar cells and amacrine cells. Future research should be directed with these goalposts in mind and with the understanding that translation from mouse to primate circuitry is imperative to make successful RGC transplantation a reality (Table 4).

**Table 4:** Future directions for inner retinal wiring (SDG4)

Research Area	Future Goals
Role of non-neuronal cells	Investigate how macroglia (Müller glia and astrocytes) and microglia modulate neural activity and contribute to synaptic plasticity in healthy retinas, disease states, and following RGC transplantation
Pathways underlying circuit development and integration	Identify the cell-intrinsic and extrinsic cues that underlie IPL patterning and circuit development and leverage this information to develop interventions that promote donor RGC integration into these circuits
Visual information processing	Use advanced imaging technologies and optical electrophysiology (e.g., two-photon and light-sheet microscopy) to study the dynamics of retinal circuits and how they respond to changes in visual stimuli. Leverage this information to better understand the mechanisms by which the inner retina processes visual information and how donor RGCs may be contributing to visual processing
Optogenetics	Employ optogenetics to selectively activate or inhibit specific cell types in the retina, enabling the study of individual neurons and their interactions with other cells

## SDG #5. Eye-to-brain connectivity

**Fig. 4:**
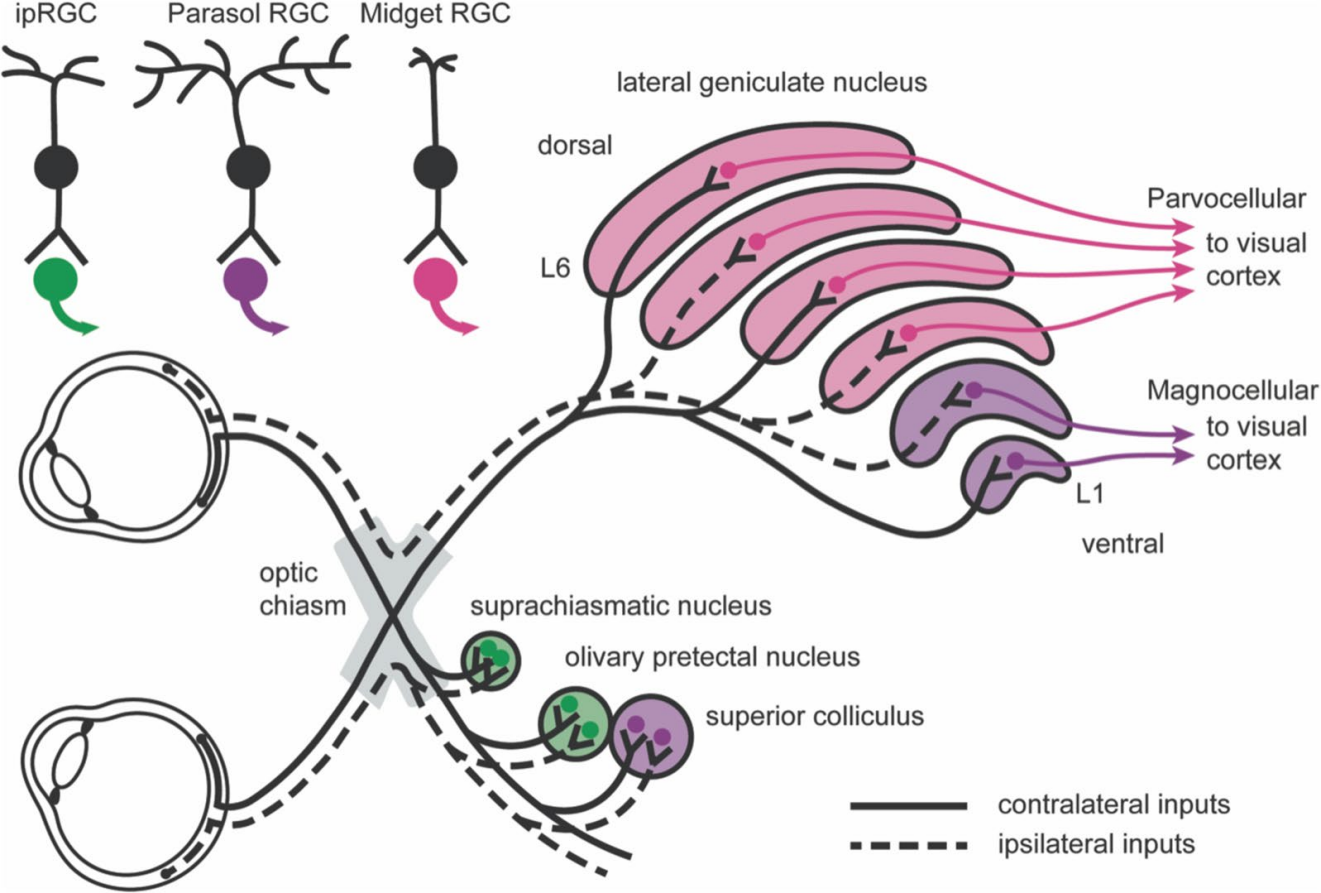
Retinal ganglion cell (RGC) pathways in the human brain. Visual information travels from each retina through the optic nerve and converges at the optic chiasm. Uncrossed ipsilateral inputs connect to L2, L3, and L5 in the lateral geniculate nucleus, whereas crossed contralateral inputs connect to L1, L4, and L6 in the lateral geniculate nucleus. Both ipsilateral and contralateral inputs connect to the suprachiasmatic nucleus, olivary pretectal nucleus, and superior colliculus. Intrinsically photosensitive RGCs (ipRGCs), among others, connect to the neurons in the suprachiasmatic nucleus and olivary pretectal nucleus (green) to regulate circadian rhythms and the pupillary light reflex, respectively. Parasol RGCs, among others, connect to the neurons in the superior colliculus (purple) to orient head and eye movements and to L1 and L2 in the lateral geniculate nucleus as a part of the magnocellular pathway (purple). Midget RGCs, among others, connect to the neurons in L3, L4, L5, and L6 of the lateral geniculate nucleus as a part of the parvocellular pathway (pink). The magnocellular and parvocellular pathways connect to the visual cortex to primarily process motion and high-contrast information, respectively

Axon (re)generation is a complex process that involves overcoming mechanical and inflammatory obstacles, identifying and responding to specific signals in the adult environment, and navigation by diverse RGC subtypes. Axon regeneration is not simply a recapitulation of development, and adult retinas may require unique cues for long-distance reinnervation of central visual targets. Additionally, the role of glia in promoting or inhibiting axon regeneration is complex and varies depending on the type of glial cell, region, and stage of axon regeneration. While glia have traditionally been thought to inhibit axon regeneration, recent studies have demonstrated that they can also be beneficial. Therefore, a comprehensive understanding of glial cell diversity throughout the visual pathway and their functions at different stages of axon regeneration is necessary to establish effective glial modulation strategies for promoting efferent connectivity to the brain (Fig. 4). This section explores the challenges and potential solutions to promoting axon regeneration over long distances, regulating the immune system and glial cell response, and targeting appropriate regions in the adult brain after potential atrophy. Developing strategies to restore vision in glaucoma and other optic neuropathies requires a comprehensive understanding of RGC diversity, development, the adult healthy and diseased microenvironment, and regeneration.

### Axon regeneration over long distances

Axon regeneration remains a significant challenge for optic neuropathy therapy development. The feasibility of RGC axon regeneration over long distances was first shown by anatomical studies demonstrating that RGCs in adult rats can regenerate axons through grafted segments of peripheral nerve tissue [402]. When such grafts are inserted directly between the retina and the brain, RGCs regrow their axons into the graft and the superior colliculus [402–404].

More recently, seminal work over the past 15 years has identified several pathways that can be effectively targeted to promote long-distance axon regeneration of injured, endogenous RGCs in rodents and represents another key advance that makes therapeutic RGC repopulation feasible. These pathways have been reviewed elsewhere and include signaling through thrombospondin-1, Lin28/IGF-1, PTEN/mTOR, suppressor of cytokine signaling 3 (SOCS3)/JAK/STAT3, KLFs, oncomodulin, transcription elongation factors, collapsin-response-mediator proteins, dynein light chains, mitochondrial leucyl-tRNA synthetase, and others [76, 377, 405–411]. It is hoped that by targeting similar pathways in transplanted RGCs, long-distance axon growth into the brain can be achieved.

Assuming RGCs can engraft into the retina, their axons will encounter a critical anatomical structure before entering the optic nerve: the lamina cribrosa. Once developmentally formed, this porous, multi-layered collagenous structure may pose a mechanical obstruction that regenerated axons must overcome to exit the eye. Indeed, significant biomechanical changes to the optic nerve head occur in advanced glaucoma. Furthermore, the lamina cribrosa is a site of neuroinflammation in glaucoma and other optic neuropathies, which can stress the axons of donor cells. Given that most work on RGC axonal regeneration has involved lesioning the optic nerve distal to the lamina cribrosa (within the orbit), there is little data that provides insight into how much of a barrier this tissue represents for axonal growth or how to circumvent it. Studies examining how intraretinal RGC axons within the optic nerve fiber layer might traverse the lamina cribrosa and optic nerve head should be a high priority.

Axon regeneration is not simply a recapitulation of developmental axonal genesis. Indeed, the receptor-ligand pairs driving zebrafish axonal regeneration and mammalian peripheral nervous system regeneration are not identical to those driving axonal development [412]. For instance, *Sprr1a* is an essential peripheral nervous system axon regeneration gene. Suppressing *Sprr1a* inhibits axon regeneration of preconditioned sensory neurons [413]. However, Sprr1a is only upregulated during regeneration and not expressed during developmental axon growth. Understanding why factors like Sprr1a are important for regenerative, but not developmental, axon growth may yield insights into the best pathways to target for promoting axon extension from donor RGCs.

Various molecular targets for RGC axon regeneration have been identified, including KLFs, thrombospondin, Oncomodulin, and others [414]. KLF4 and KLF9, part of the KLF family of transcription factors, inhibit RGC axon regeneration, and their deletion has resulted in notable axon growth in experimental models [415, 416]. Thrombospondin-1 and -2, matricellular proteins important for synapse formation and neuronal plasticity, are potential therapeutic targets for RGC axon regeneration [417, 418]. Oncomodulin binds to RGCs in a cyclic adenosine monophosphate-dependent manner and stimulates optic nerve regeneration [419]. Importantly, these combinatorial approaches promote axon regeneration more than isolated manipulations. *Pten* deletion, *Socs3* deletion, and *Cntf* overexpression have synergistic effects on RGC survival and axon regeneration [374]. To support long-distance axonal regeneration, global changes in metabolism should be a point of focus since lipids are needed to build the considerable volume of plasma membrane needed to traverse the optic nerve [420]. Indeed, *Socs3* knockout and *Pten* knockout-mediated axon regeneration depend on metabolic changes in RGCs to support lipid synthesis [421].

Axon guidance cues are expressed in both spatial and temporal gradients during different stages of development. For example, in the developing *Drosophila* CNS, to decussate, growing axons must exchange cell surface receptor FasII (Fasciclin II) for FasI. Once decussation is complete, neurons must switch back to expressing only FasII [422]. Successful recapitulation of these signaling mechanisms in the adult animal using current molecular techniques has been limited. Although some guidance cues from development persist in adults, it remains unclear whether they function similarly post-development. While the signals that direct RGC axon outgrowth during development have been well studied [423, 424], the expression patterns of these molecules in the post-developmental visual pathway are less well characterized. Fundamental to our ability to leverage intrinsic guidance cues to promote donor RGC axon regeneration will be the development of comprehensive atlases of guidance cue expression throughout the visual pathway as a function of age and disease state. Rather than relying on the maintenance of expression of these cues in the adult, another approach to promoting long-distance axon regeneration is to engineer RGCs to respond to the remaining signals by introducing the appropriate guidance receptors.

Co-culture systems of RGCs with their pre- and postsynaptic targets are one approach to understanding how donor RGCs will interact with the host microenvironment. For example, donor RGCs can be co-cultured with dissociated primary retinal cells to determine whether they will recognize and respond to chemotropic cues in the retina [425]. In this setting, donor RGC neurite complexity and axon length have increased significantly when co-cultured with central versus peripheral retinal cells. This suggests they may respond to a centripetal gradient of intra-retinal guidance cues for orientation towards the optic nerve head. Similarly, by explanting retinorecipient targets from the brain and co-culturing those tissues in vitro, donor RGC axon guidance towards relevant visual targets in the brain can be assessed [19].

In other than the most advanced cases of glaucoma, surviving endogenous RGCs might serve as guidance tracks for new RGCs once transplanted within the retina [426]. Indeed, interactions between donor and host RGC axons within the retinal nerve fiber layer might help guide donor axons to the optic nerve head. Axon guidance during development and in vitro is driven by surface topography or signaling through cell surface receptors and ligands [427, 428]. In particular, molecules such as Sema6D, neuronal cell adhesion molecule (Nr-CAM), and Plexin-A1 that are expressed on midline radial glia and chiasmal neurons play a role in this process [429]. Sema6D typically acts as a repellant for RGC axons, but when combined with Nr-CAM and Plexin-A1, it promotes growth instead. Interestingly, the radial glial marker, slit guidance ligand 1, which is also usually considered to act as an inhibitory guidance cue during development, has been identified in the adult optic chiasm after an optic nerve crush injury, while other markers (radial glial cell marker 2, brain lipid binding protein) remain absent [430]. Lastly, recent research has shown that bone morphogenetic protein 4 signaling interferes with optic chiasm formation and RGC pathfinding [431]. Considering the varying expression of these guidance cues during development, adulthood, and following injury, it remains unclear whether similar axon-specific interactions exist in the retina. Variations in these cues may contribute to the misguidance of regenerating RGC axons through the optic chiasm, or regenerating axons may lack the receptors for those cues.

The optic chiasm is a cross-shaped junction in the brain where the optic nerves meet and a proportion of RGC axons decussate. This structure is crucial for binocular vision by enabling the integration of visual information pertaining to overlapping regions of the visual field from both eyes. RGC axons either cross to the opposite side (contralateral) or remain on the same side (ipsilateral) at the chiasm, depending on specific guidance cues. In many species, the pattern of decussation in the chiasm is influenced by a range of molecular signals. For instance, guidance molecules such as Netrin-1 and Slit proteins, and their respective receptors, play a role in determining which axons cross and which do not [432]. In mice, most RGC axons project contralaterally, with only a small proportion, approximately 3–5%, projecting ipsilaterally [433]. Conversely, in primates, including humans, the proportion of ipsilaterally projecting RGC axons is higher, with estimates suggesting that approximately 45–55% of RGC axons project contralaterally, while the rest remain ipsilateral [433]. While mouse models offer numerous advantages for research, these differences in RGC projections between species necessitate caution when selecting animal models for translational applications. Further emphasis is needed to understand the full spectrum of factors influencing RGC axon guidance in the context of retinal transplants across species.

Electrophysiologic activity is another cell-intrinsic factor that mediates axon regeneration. Enhanced neural activity by visual stimulation or chemogenetics increases axon regeneration of injured RGCs [434]. Moreover, increasing neural activity, in combination with the elevation of mTOR, further promotes RGC axon regeneration over the long distances necessary to re-innervate the brain. As such, enhancing the electrophysiologic activity of donor RGCs may augment optic nerve regeneration, though the optimal induction methods and activity patterns remain to be determined.

The diversity of RGC subtypes must also be considered. Different subtypes require different guidance cues for directing axon regeneration, and it is still unclear to what extent such cues may be available endogenously or need to be provided experimentally [435]. Also, some RGC subtypes survive injury better than others or are more likely to regenerate axons following axotomy [6, 149]. Moreover, different RGC subtypes respond differently to gene knockouts. For example, while *Pten* inhibition causes mostly alpha-RGCs to regenerate axon short-distances [374], it is mostly M1 ipRGCs that regenerate axons long-distance [76]. A unique feature of ipRGCs is the expression of photosensitive melanopsin, encoded by the *Opn4* (*melanopsin*) gene, which enables these RGCs to fire action potentials in response to light. Similar to previous work showing improved axon regeneration following electrical stimulation [1], the ability for ipRGCs to be directly activated by light may contribute to their enhanced survivability and axon regeneration capacity [434]. Furthermore, overexpression of *Opn4* itself also promotes axon regeneration [436]. Therefore, combinatorial strategies involving co-targeting different pathways while also stimulating electrical activity will be needed to promote long-distance axon regeneration across multiple RGC subtypes.

Nonetheless, regeneration or replacement of only a specific subset of these RGC subtypes might be sufficient to restore rudimentary forms of vision and drastically improve a patient’s quality of life. Vision restoration extends beyond just providing functional vision; it also contributes to regulating the circadian rhythm, which relies on light cues. ipRGCs play a crucial role here, directly responding to light and regulating our internal "clock." Patients with Non-24-Hour Sleep–Wake Disorder, a condition common in people who are blind, suffer from disturbed sleep patterns due to a lack of light perception. Restoring even basic light perception could re-synchronize their sleep–wake cycle and improve their life quality significantly. Current treatments for Non-24-Hour Sleep–Wake Disorder are pharmacological, costly, and have limited effectiveness. A targeted therapy that regenerates specific RGC subtypes could offer a more cost-effective, long-lasting solution. Moreover, ipRGCs project to various vision-processing regions in the brain and regulate other non-image-forming functions, such as the light-evoked constriction of the pupil, and are even involved in contrast sensitivity and visual perception [437]. Hence, treatments aimed at ipRGCs could be promising. However, for research purposes, the aspiration is set above this goal. Short-term goals resulting in clinically significant patient outcomes should be prioritized while striving towards long-term goals of functional vision restoration.

### Role of glia in axon regeneration

Glia, in particular reactive astrocytes and microglia, or damaged and dying oligodendrocytes, have traditionally been thought to inhibit axon regeneration due to failure to clear myelin byproducts, including Nogo, MAG, and OMgp, that are released during nerve damage. However, recent studies have revealed that glial cells can also play a beneficial role in axon regeneration. For instance, microglial depletion disrupts the growth of long projecting axons beyond the lesion after spinal cord injury in neonatal mice, suggesting they play an active role in regeneration [438]. Similarly, microglia depletion exacerbates tissue damage and worsens functional recovery after contusion spinal cord injury in adult mice [439]. These findings suggest that the role of glia, at least microglia, in axon regeneration is complex, and it is oversimplistic to classify their behavior as binary: beneficial or inhibitory.

The complexity of glial functions arises from the diverse types of glial cells [440], each of which plays a different role in axon regeneration. Microglia primarily serve immune-related functions and clearance of debris. Astrocytes provide structural and metabolic support and regulate the microenvironment of neurons. MG are the most abundant glia in the retina and have a radial morphology extending from the ILM to the photoreceptor outer segments. Their basal lamina forms the first barrier that must be traversed by RGC transplanted intravitreally. MG also engulf and contact the blood vessels responsible for regulating the extracellular environment and supporting neuronal survival. The interactions and coordination between these different glial cell types are essential for the proper functioning and homeostasis of the retina [354]. For example, MG secret factors are necessary for axon regeneration and RGC survival [441].

Importantly, there is extreme heterogeneity between glial cells of the same type throughout the CNS. For instance, astrocytes in the retina have unique phenotypes (e.g., transcriptomic and proteomic profiles) compared to those in the optic nerve, and those in the optic nerve head have different functions from those in the myelinated part of the optic nerve or the brain and spinal cord. In the retina alone, there are three at least morphological subclasses of astrocytes: bipolar astrocytes that run along nerve fiber bundles, perivascular astrocytes, and stellate astrocytes that occupy the spaces between blood vessels and nerve fibers [440]. These astrocytes all support retinal neurons and regulate pH and ion levels, but it remains unclear if they have subtype-specific functions that are not easily transferred to astrocytes in other regions (an important factor for consideration in cell transplant therapies). They interact with other glial cells and neurons, influencing synaptic function and participating in vascular development and neurovascular coupling [442]. In the optic nerve, oligodendrocytes wrap axons in myelin, enabling rapid transmission of visual information, maintaining extracellular ion concentrations, and contributing to metabolic substrate delivery. To varying extents across species, astrocytes contribute to the integrity of the lamina cribrosa (or, in rodents, the glial lamina), supporting optic nerve fibers as they exit the eye.

The stage of axon regeneration is a critical factor that influences the overall effect of glial modulation [443]. In acute phases following an injury, microglia, and to a lesser extent astrocytes, clear the myelin debris from degenerating oligodendrocytes and support regeneration. In the context of inflammation and disease, an intriguing division of labor occurs between microglia and astrocytes – with microglia (largely) increasing phagocytic capacity, while some sub-states of reactive astrocytes shutting down phagocytosis almost completely [264]. Modulating glial cells to improve their ability to clear debris may be necessary in cases where excess myelin debris is particularly inhibitory. There is a window of opportunity during which glial cells must be modulated or activated to optimize their clearance functions. If myelin debris persists for an extended period, it can impede regeneration and lead to detrimental effects [444]. Moreover, these clearance and regeneration roles seem to be specialized, with perivascular microglia controlling the entry of materials into the retina from the vasculature and parenchymal microglia being highly motile cells that survey the microenvironment, clear debris, and mediate synapse remodeling [445]. Therefore, promoting or suppressing glial reactivity should be timed appropriately to ensure optimal results depending on the stage of degeneration and regeneration. A comprehensive understanding of glial diversity at different stages of optic neuropathy is necessary to establish effective glial modulation strategies for promoting axon regeneration while disrupting inhibitory cues. For example, glial cells can be reprogrammed to overcome glial cell inhibition of regeneration and promote structural and functional regeneration after CNS injury by increasing glycolysis [446], while post-injury-born oligodendrocytes incorporate into the glia scar and hinder experimental axon regeneration by presenting myelin-associated inhibitors to the growing axons in an attempt to myelinate them (before they reached respective post-synaptic targets) [447].

It is worth considering that most current knowledge about glial cell contributions to retina/visual system degeneration and regeneration comes from injury models [447, 448], and it is crucial to investigate how this knowledge translates to neurodegenerative diseases that affect human patients. Injury models do not necessarily mimic the chronic nature of neurodegenerative diseases, and the function of glial cells is directly altered in neurodegenerative diseases [449, 450]. Interestingly, unilateral optic nerve damage results in a contralateral glial response [451, 452]. Better understanding the factors that drive contralateral gliosis and development methods to modulate reactive gliosis in each eye (with comparison to bilaterally naïve controls) will be relevant to developing RGC repopulation and protection strategies.

### Targeting appropriate visual areas in the brain

Targeting appropriate visual systems in the brain is crucial for successful optic nerve regeneration and vision restoration in optic neuropathies. Co-culture experiments involving RGCs, retinal organoids, or retinal explants with organoids of thalamic tissue (i.e., assembloids) may inform methods for guiding RGC axons to the correct locations in the brain [19]. Leveraging the extensive information gained from studies promoting the regeneration of endogenous RGC axons following injury will also aid the development of strategies to guide donor RGC axons to subcortical visual centers. However, once RGC axons reach their central targets, rebuilding topography will likely be necessary for image-preserving vision. Studies in lower vertebrates, such as zebrafish, may help elucidate how spatial and temporal information is encoded across visual space [453] and can be used to identify mechansims essential for RGC target innervation [454].

Regulating neural activity with exogenous electrical field simulation may help to direct axon regeneration [455–457]. Controlling neural activity may also hold potential in training rudimentary visual functions. One approach for increasing the receptivity of the postsynaptic targets in the brain to innervation is modifying it using chemogenetic tools [458]. For example, by enhancing neural activity in postsynaptic neurons in the optic pathway (the pretectal nucleus), endogenous RGC axons can regenerate and reconnect to their brain targets in a distal injury model [458]. In addition, augmentation of axon conduction velocity can help overcome prolonged latency of remyelination after axon reinnervation [459, 460]. This work highlights the potential of targeted brain stimulation to regenerate endogenous RGC connections. However, no studies have examined the retinotopic projections of regenerating *donor* RGC axons within central targets. Careful examination of retinotopic patterning of regenerated RGC axons within the lateral geniculate nucleus, determining whether there is plastic evolution in retinotopic mapping over time, and developing methods to control this process are key areas for future research.

Several experiments have identified atrophy in the visual processing centers of the brain following monocular optic nerve crush across multiple species, and anterograde transsynaptic degeneration appears to be a feature of many human optic neuropathies [461]. Within 90 days of RGC death and an accompanying loss of retinal outputs, the superior colliculus, dorsal lateral geniculate nucleus, and visual cortex experience shrinkage, molecular changes, reduced neural activity, and cell loss [461–467]. Moreover, dark-rearing zebrafish results in reduced brain size [468], indicating the potential importance of visual stimulation for developing and maintaining retinorecipient neurons in the brain. Early intervention after an injury can prevent RGC death, promote optic nerve regeneration, and partially restore vision [75, 434, 469]. Determining the extent to which postsynaptic targets will be receptive to newly established innervation in advanced disease is an important task for the field. Moreover, defining the sequence and timeline of transsynaptic degeneration in various optic neuropathies will help inform the optimal timing for therapeutic innervation. Other subcortical targets, such as the superior colliculus, are also necessary for vision, though the relative contribution of these areas to vision may vary across species. Determining which brain targets are most essential for processing visual information will help guide strategies to target donor RGC axons to the most critical locations.

Functional tests are the most rigorous methods to evaluate the success of optic nerve regeneration and include testing of visual acuity, contrast sensitivity, and electrophysiology function within the brain (e.g., through visually evoked potentials). Unfortunately, results in early transplantation experiments may not yield enough visual pathway regeneration to discern appreciable improvements in behavior. Thus, histology will likely remain the gold standard for assessing optic nerve regeneration in the near term. Future efforts to establish more sensitive approaches to assess donor RGC axon regeneration within the brain in vivo*,* such as with implantable bioelectronics [470, 471], would be valuable.

### Models to study optic nerve regeneration

In vitro models used to study optic nerve regeneration, including retinal explants, purified primary RGCs, patient-derived induced pluripotent stem cell-derived RGCs, and retinal organoids, offer several advantages, such as ease of manipulation of experimental conditions, reproducibility between experiments, and the ability to use human retinal cells. However, these in vitro models also have several limitations. For example, retinal organoids differ in structure and cell composition from the human eye and lack peripheral circulation and immune surveillance, limiting their usefulness in studying visual circuit assembly and disassembly. Moreover, the limited lifespan of RGCs within organoids is a significant drawback to their long-term study in vitro.

Co-culturing retinal organoids with brain tissue may provide postsynaptic contacts for RGCs and overcome some of these limitations, including the limited lifespan and lack of visual circuits in other in vitro systems. Assembloids, created by fusing retinal and thalamic organoids, exhibit decreased RGC apoptosis compared to retinal organoids grown in isolation [19]. In addition to improved survival, some RGC axons extend to and enwrap their postsynaptic targets while others grow into co-cultured cerebral organoids [19]. Co-cultures of the visual centers of the postnatal or embryonic brain with stem cell-derived retinal organoids may represent a powerful approach to studying axonal growth and guidance, particularly if the optic chiasm can be adequately modeled in this system.

To aid in studying axon outgrowth and regeneration, microfluidic chips or nanofibers can direct axons in monolayer and 3D organoids/assembloids [19]. Providing soluble factors in specific compartments of these microfluidic devices is useful as a screening approach to identify cues that drive axonal guidance. Conversely, these tools could identify negative influencers in unhealthy environments that inhibit outgrowth.

Studying regeneration through in vitro experiments will greatly enhance our knowledge, but it is important to also determine how to regenerate retinal connections in vivo. In vivo*,* assays usually evaluate regeneration after an optic nerve crush or induction of high ocular pressure to mimic glaucoma. Important questions include which animal model to use and how to assess regeneration in a consistent matter. Given the wide availability of transgenic animals, rodents are commonly used as an animal model to study optic nerve regeneration. However, rodents are nocturnal animals relying less on vision for day-to-day activities than humans. The absence of a macula and lamina cribrosa also limits their utility as a model system. Pigs are becoming increasingly popular as a model for studying optic nerve regeneration due to the increasing availability of transgenic lines that mimic various optic neuropathies [472, 473]. Feline models have also been used to study optic nerve degeneration, neuroprotection, and RGC transplantation [246, 474].

While other animal models, such as reptiles, amphibians, and fish, can regenerate RGC axons spontaneously, they may still provide valuable insights into this field [475–477]. For example, optic nerve crush in tadpoles and zebrafish [478–480] has been used to study RGC axon regeneration, in this case, from endogenous RGCs. Mammals, including rodents, however, do not regenerate CNS projection neurons spontaneously. Therefore, mice are often used as a model system to study the failure of optic nerve axon regeneration, particularly using optic nerve transection or crush models.

The optic nerve crush injury model is the most widely used in vivo model system. However, there is considerable variability in how optic nerve crush is performed, including the duration of the crush, the type of forceps used, and the extent of injury to the nerve [481]. Partial crushes can lead to extensive variability and reduced reliability compared to total crushes. When studying optic nerve regeneration, a total crush is preferred to prevent surviving RGC axons from being misclassified as regenerating fibers [482]. Several methods, including functional testing with visually evoked potentials, can be used to verify the completeness of the crush [483]. Cholera toxin subunit B is often used to label axons in optic nerve crush, but its effectiveness can be variable depending on the extent of axon damage [484, 485]. Lastly, while optic nerve crush is the standard model used in optic nerve regeneration research, an important limitation of optic nerve crush is its acute nature, making it less suitable for studying chronic and progressive optic neuropathy, such as glaucoma. However, this characteristic also advantageous for studying the mechanisms of optic nerve regeneration without the confounding factors present in more complex optic neuropathies [486].

Ocular hypertension models of glaucoma are also widely used and include the injection of microbeads or silicone oil into the anterior chamber, hypertonic saline injection into the episcleral veins, perilimbal constriction sutures, and genetic models of ocular hypertension. A major challenge with studying axon regeneration in ocular hypertensive glaucoma stems from their chronic progressive nature and difficulties distinguishing regenerated axons from undamaged, surviving axons. Maintaining a state of elevated IOP for prolonged periods of time leading to very severe optic neuropathy may increase the consistency of experiments utilizing glaucoma models to study optic nerve regeneration. Nevertheless, the choice of a model for studying optic nerve regeneration or glaucoma should be based on specific research questions, and it may be beneficial to study multiple models to obtain a more comprehensive understanding of the processes involved. Indeed, while still in its infancy, whole eye transplantation in rats [487, 488] could represent another model to study optic nerve regeneration outside a disease state.

As discussed, many other animal models have been developed to study other types of optic nerve disease that may help inform RGC axonal regeneration. For example, intraperitoneal injection of myelin-associated glycoprotein or aquaporin-4 protein injection into the subarachnoid space under the optic nerve sheath is employed to study demyelinating optic neuropathies [489]. Frequency double-YAG laser has been used to selectively activate intravascular Rose Bengal dye in the vessels that supply the optic nerve head to induce thrombosis of small capillaries while sparing larger vessels such as the central retinal artery as a model system of ischemic optic neuropathy [490]. Studying RGC axonal regeneration or donor RGC axonal guidance in these models may provide relevant insights into RGC responses to varying optic nerve insults.

### Critical period for the regeneration of the visual system

The timeline for therapeutic regeneration is crucial for successful vision restoration in optic neuropathies. In animal models, such as the mouse optic nerve crush injury model, it has been found that almost 90% of RGCs die within the first three weeks after injury [140]. However, injury models do not fully mimic neurodegenerative diseases like glaucoma, and there are inter-species differences between mice and humans. Irrespective of species, long-term denervation, because of RGC death, can cause shrinkage and molecular changes in target regions in the brain [461]. While efforts have been made to prevent superior colliculus and dorsal lateral geniculate nucleus degeneration using neuroprotection, these tissues continue to atrophy [462], seemingly due to a lack of retinal inputs. Prompt initiation of axonal regeneration following peripheral nervous system injuries is associated with reduced degeneration at postsynaptic targets and better functional outcomes [491]. Altogether, these observations suggest that developing early interventions to maintain the postsynaptic targets in the brain after RGC loss may be valuable. Moreover, the initial clinical translation of optic nerve degeneration approaches in humans may be most successful if it targets patients with relatively acute and recent vision loss. While this may not be possible in most cases of patients with glaucoma, future efforts may need to focus on developing strategies to regenerate retinorecipient tissue in the brain to maximize the potential for vision restoration.

### Future directions for eye-to-brain connectivity

A combinatorial approach to advancing methods of promoting eye-to-brain connectivity must be considered and should combine work investigating signaling pathways necessary for neuronal survival, axon regeneration, and chemotropic guidance of axons. Given the long distance of RGC axonal projections and the complexity of postsynaptic wiring in humans, experimental models that resolve regenerating axons at the single axon fiber level will be needed. This approach may also enable researchers to investigate the role of neural activity and panels of guidance cues in axon regeneration since RGC subtypes are likely to differ in their responsivity to specific signals. A relatively unexplored area of investigation is the development of strategies that aid donor RGCs in connecting with postsynaptic target neurons in the brain. Investigators should also explore mechanisms underlying the retinotopic mapping of synapses and consider ways to manipulate this process so that incoming signals to the lateral geniculate nucleus will be interpretable.

Future studies should undertake multimodal investigations of glial function during development, degeneration, and axon regeneration within the visual pathway. Single-cell sequencing, optogenetics, chemogenetics, and transgenic models might be used to identify specific roles of glial subpopulations in promoting or inhibiting axon regeneration (Table 5).

**Table 5:** Future directions for eye-to-brain connectivity (SDG5)

Research Area	Future Goals
Combinatorial approaches to multimodal reinnervation of the brain	Implement a combinatorial approach to understand different signaling pathways necessary for neuronal survival, axon regeneration, and guidance to direct eye-brain connectivity
Distal injury models and in vitro models	Employ these models to simplify experimentation, study regeneration at the single axon level, and investigate the role of neural activity in axon regeneration. Different RGC subtypes may require different guidance cues
Role of glial cells	Use techniques such as single-cell sequencing, optogenetics, chemogenetics, and transgenic mouse lines to identify specific roles of glial subpopulations in promoting or inhibiting axon regeneration. This may include physiologically ‘normal’ glial subtypes, or one of many reactive glial sub-states
Overcoming mechanical blockages	Explore the development of strategies that aid donor RGCs in connecting with downstream neurons in the brain, specifically overcoming the mechanical blockage of the lamina cribrosa
Adult retinal and brain microenvironment	Investigate the spatial–temporal expression/induction of guidance signals in the adult environment and engineer RGCs to respond to specific cues present in the adult retina to promote axon regeneration
Neural Activity	Investigate the role of neural activity in axon regeneration among RGC subtypes to develop combinatorial strategies for promoting regeneration broadly
Retinotopic mapping	Evaluate whether regenerating RGC axons synapsing at subcortical visual centers establish a retinotopic map and develop methods for modulating this process
Brain regeneration	Develop strategies to regenerate retinorecipient tissue in the brain in optic neuropathies, which may overcome issues with anterograde transsynaptic degeneration in longstanding optic neuropathy

## Conclusions and future directions

RGC death is a major cause of irreversible vision loss, and regenerative approaches for restoring vision lost to optic neuropathies are crucial. Since ophthalmology is at the forefront of regenerative cell therapy, the RReSTORe Consortium was organized to address the challenges associated with RGC repopulation. Through multiple in-depth virtual and face-to-face discussions, members of the RReSTORe consortium have built consensus and identified the most pressing challenges, questions, and suggested approaches that must be addressed to bring RGC repopulation closer to clinical translation. As is often the case for fields on the cusp of transformative progress, discussions within the RReSTORe Consortium have yielded more questions than answers. Through collaborative experimental efforts, the scientists who are a part of this consortium will help to advance the field and bring hope to patients suffering from severe optic neuropathy. While significant obstacles remain, recent scientific advances described here suggest that functional RGC repopulation in humans suffering from optic neuropathy may be feasible, and they provide a roadmap for continued scientific progress.

While consensus building and groupthink can unify a scientific community in its pursuits, we acknowledge that they do not always propel groundbreaking discoveries. Indeed, the visual science field, despite concerted efforts, has yet to develop a definitive cure for optic neurpathic vision loss or even a neuroprotective treatment that substantially slows disease progression. We recognize this criticism and emphasize that the perspectives presented in this manuscript are not exhaustive and should not be viewed as the only path forward. We hope this roadmap can serve as a foundation and a springboard for diverse perspectives and unconventional ideas from the broader research community, including those outside of the consortium, to propel the field forward. Investigators who may wish to join the consortium can find further information at http://rrestore.info, or by contacting the corresponding author.

## Data Availability

Not applicable.

## Appendix

### RReSTORe Consortium Author Block

Abdelrahman Y. Fouda^1^, Aboozar Monavarfeshani^2^, Adriana Di Polo^3^, Ahmara G. Ross^4^, Ajay Ashok^5,6^, Ala Moshiri^7^, Alain Chedotal^8^, Alex L. Kolodkin^9^, Amberlynn A. Reed^10^, Amjad Askary^11^, An-Jey A. Su^12^, Anna La Torre^7^, Archana Jalligampala^13^, Ariadna Silva-Lepe^14^, Arupratan Das^15^, Barbara Wirostko^16^, Benjamin J. Frankfort^17^, Benjamin Sivyer^18,19^, Bhagwat Alapure^20^, Brad Fortune^21^, Brent Young^22^, Brian C. Samuels^23^, Brian Clark^24^, Bryan William Jones^16^, Carol A. Mason^25^, Casey Keuthan^26^, Chase Hellmer^13^, Claire Mitchell^4^, Claire Ufongene^27^, Dan Goldman^28^, David Feldheim^29^, David H. Gutmann^30^, David J. Calkins^31^, David Krizaj^16^, David M. Gamm^32^, Derek Welsbie^33^, Diana C. Lozano^18^, Diane E. Bovenkamp^34^, Donald J. Zack^26^, Dong Feng Chen^5,6^, Elena Vecino^35^, Ephraim F. Trakhtenberg^36^, Erika A. Aguzzi^37^, Feng Tian^6^, Fengquan Zhou^38^, Gillian J. McLellan^32^, Harry A. Quigley^26^, Hashem Abu Serhan^39^, James R. Tribble^40^, Jason Meyer^15^, Jeff Gross^41^, Jeff S. Mumm^26^, Jeffrey L. Goldberg^22^, Jeremy M. Sivak^42,43^, Jingliang Simon Zhang^26^, Jiun L. Do^33^, Jonathan Crowston^44^, Jonathan R. Soucy^5,6^, Julie Chen^5,6^, Julie Cho^22^, Juliette McGregor^45^, Juliette Wohlschlegel^46^, Kalyan C. Vinnakota^47^, Kang-Chieh Huang^17^, Karen Peynshaert^48^, Katherine E. Uyhazi^4^, Keith Martin^49,50,51^, Ken Muller^52^, Kevin K. Park^52^, Kimberly K. Gokoffski^53^, Kin-Sang Cho^5,6^, Kun-Che Chang^54^, Larry Benowitz^2,6,54^, Leonard A. Levin^55^, Levi Todd^46^, Lies De Groef^56^, Lieve Moons^56^, Luis Alarcon-Martinez^51,49^, Mandeep S. Singh^26^, Manuel Vidal-Sanz^57^, Mariana S. Silveira^58^, Marina Pavlou^46^, Matthew B. Veldman^59^, Matthew Van Hook^60^, Meher A. Saleem^52^, Melanie Samuel^17^, Mengming Hu^59^, Micalla Peng^53^, Michael James Gilhooley^37^, Michael Young^5,6^, Michel Cayouette^61^, Mohammad H. Geranmayeh^62^, Mollie Woodworth^22^, Monica Vetter^16^, Nicholas R. Marsh-Armstrong^7^, Pete A. Williams^63^, Petr Baranov^5,6^, Pratheepa Kumari Rasiah^64^, Preeti Subramanian^34^, Qi N. Cui^4^, Rebecca M. Sappington^65^, Reem Amine^66^, Richard Eva^50^, Robert J. Johnston Jr.^26^, Roman J. Giger^28^, Ross Ethier^67,68^, Sadaf Abed^29^, Sehrish Nizar Ali Momin^69^, Seth Blackshaw^26^, Shane A. Liddelow^70^, Stella Mary^26^, Stephen Atolagbe^71^, Supraja Varadarajan^22^, Tareq I. Nabhan^72^, Tasneem Khatib^50^, Tasneem Putliwala Sharma^15^, Thomas A. Reh^46^, Thomas Brunner^73^, Thomas V. Johnson^26^, Tom Greenwell^10^, Tonia S. Rex^31^, Trent Watkins^74^, Tudor C. Badea^75^, V Vrathasha^4^, Venkata Ramana Murthy Chavali^4^, Viviane M. Oliveira-Valença^58^, Wai Lydia Tai^5,6^, William Guido^13^, Wyndham M. Batchelor^10^, Xian-Jie Yang^76^, Xue-Wei Wang^9^, Yong Park^17^, Yuan Pan^77^, Yvonne Ou^74^, Ziming Luo^22^

1. University of Arkansas for Medical Sciences
2. Boston Children’s Hospital
3. University of Montreal
4. University of Pennsylvania
5. Schepens Eye Research Institute
6. Harvard Medical School
7. University of California, Davis
8. Institut de la Vision, Sorbonne Université, INSERM, CNRS
9. Johns Hopkins University School of Medicine
10. National Eye Institute
11. University of California, Los Angeles
12. University of Colorado
13. University of Louisville
14. Instituto Mexicano de Oftalmología
15. Indiana University
16. University of Utah
17. Baylor College of Medicine
18. Casey Eye Institute
19. Oregon Health & Science University
20. Louisiana State University Health Sciences Center
21. Devers Eye Institute
22. Stanford University
23. University of Alabama, Birmingham
24. Washington University School of Medicine
25. Columbia University
26. Johns Hopkins University
27. Icahn School of Medicine at Mount Sinai
28. University of Michigan
29. University of California, Santa Cruz
30. Washington University
31. Vanderbilt University Medical Center
32. University of Wisconsin, Madison
33. University of California, San Diego
34. BrightFocus Foundation
35. University of the Basque Country
36. University of Connecticut School of Medicine
37. University College London
38. Zhejiang University School of Medicine
39. Hamad Medical Corporations
40. Karolinska Institutet
41. University of Texas at Austin
42. University Health Network
43. University of Toronto School of Medicine
44. University of Sydney
45. University of Rochester
46. University of Washington
47. Gilbert Family Foundation
48. Ghent University
49. University of Melbourne
50. University of Cambridge
51. Centre for Eye Research Australia
52. University of Miami
53. University of Southern California
54. University of Pittsburgh
55. McGill University
56. Katholieke Universiteit Leuven
57. University of Murcia
58. Federal University of Rio de Janeiro
59. Medical College of Wisconsin
60. University of Nebraska Medical Center
61. Montreal Clinical Research Institute (IRCM)
62. Tabriz University of Medical Sciences
63. St. Erik Eye Hospital & Karolinska Institutet
64. Vanderbilt University
65. Wake Forest School of Medicine
66. Cleveland Clinic
67. Georgia Institute of Technology
68. Emory University
69. Aga Khan University Hospital
70. NYU Grossman School of Medicine
71. Sumy State University
72. University of Missouri
73. Glaucoma Research Foundation
74. University of California, San Francisco
75. Transylvania University of Brasov, Romania
76. Univeristy of California, Los Angeles
77. UT MD Anderson Cancer Center

## Abbreviations

AO: Adaptive optics
BAX: BCL2-Associated X protein
BDNF: Brain-derived neurotrophic factor
CNS: Central nervous system
CNTF: Ciliary neurotrophic factor
DLK: Dual leucine zipper kinase
ERG: Electroretinography
GCL: Ganglion cell layer
HLA: Human leukocyte antigen
iPSCs: Induced pluripotent stem cells
ILM: Inner limiting membrane
IPL: Inner plexiform layer
IL-1α: Interleukin 1 alpha
IOP: Intraocular pressure
IVT: Intravitreal
ipRGC: Intrinsically photosensitive RGC
KLF: Kruppel-Like Factor
LKZ: Leucine zipper kinase
mTOR: Mammalian target of rapamycin
MG: Müller glia
NF1: Neurofibromatosis type 1
Nr-CAM: Neuronal cell adhesion molecule
OPG: Optic pathway glioma
OCT: Optical coherence tomography
PTEN: Phosphatase and tensin homolog
RGC: Retinal ganglion cell
RPC: Retinal progenitor cells
RReSTORe: RGC Repopulation, Stem cell Transplantation, and Optic nerve Regeneration
SLO: Scanning laser ophthalmoscopy
C1q: Subcomponent q
SR: Subretinal
SDG: Subtopic discussion group
SOCS3: Suppressor of cytokine signaling 3
TNF: Tumor necrosis factor

## References

[1] Goldberg JL, Espinosa JS, Xu Y, Davidson N, Kovacs GTA, Barres BA. Retinal ganglion cells do not extend axons by default promotion by neurotrophic signaling and electrical activity. Neuron. 2002;33:689–702. 11879647 10.1016/s0896-6273(02)00602-5

[2] Carelli V, Morgia CL, Ross-Cisneros FN, Sadun AA. Optic neuropathies: the tip of the neurodegeneration iceberg. Hum Mol Genet. 2017;26:R139–50. 28977448 10.1093/hmg/ddx273 PMC5886475

[3] Gokoffski KK, Peng M, Alas B, Lam P. Neuro-protection and neuro-regeneration of the optic nerve: recent advances and future directions. Curr Opin Neurol. 2020;33:93–105. 31809331 10.1097/WCO.0000000000000777 PMC8153234

[4] Sharma R, Bose D, Maminishkis A, Bharti K. Retinal pigment epithelium replacement therapy for age-related macular degeneration: are we there yet? Annu Rev Pharmacol. 2020;60:553–72. 10.1146/annurev-pharmtox-010919-023245 PMC8783375 31914900

[5] Patterson SS, Bembry BN, Mazzaferri MA, Neitz M, Rieke F, Soetedjo R, et al. Conserved circuits for direction selectivity in the primate retina. Curr Biol. 2022;32:2529-2538.e4. 35588744 10.1016/j.cub.2022.04.056 PMC9205626

[6] Tapia ML, Nascimento-dos-Santos G, Park KK. Subtype-specific survival and regeneration of retinal ganglion cells in response to injury. Front Cell Dev Biol. 2022;10:956279. 36035999 10.3389/fcell.2022.956279 PMC9411869

[7] Rheaume BA, Jereen A, Bolisetty M, Sajid MS, Yang Y, Renna K, et al. Single cell transcriptome profiling of retinal ganglion cells identifies cellular subtypes. Nat Commun. 2018;9:2759. 30018341 10.1038/s41467-018-05134-3 PMC6050223

[8] Kim US, Mahroo OA, Mollon JD, Yu-Wai-Man P. Retinal ganglion cells—diversity of cell types and clinical relevance. Front Neurol. 2021;12:661938. 34093409 10.3389/fneur.2021.661938 PMC8175861

[9] Aguayo AJ, Rasminsky M, Bray GM, Carbonetto S, McKerracher L, Villegas-Pérez MP, et al. Degenerative and regenerative responses of injured neurons in the central nervous system of adult mammals. Philos Trans Royal Soc Lond Ser B Biological Sci. 1991;331:337–43. 10.1098/rstb.1991.0025 1677478

[10] Venugopalan P, Wang Y, Nguyen T, Huang A, Muller KJ, Goldberg JL. Transplanted neurons integrate into adult retinas and respond to light. Nat Commun. 2016;7:10472. 26843334 10.1038/ncomms10472 PMC4742891

[11] Bei F, Lee HHC, Liu X, Gunner G, Jin H, Ma L, et al. Restoration of Visual Function by Enhancing Conduction in Regenerated Axons. Cell. 2016;164:219–32. 26771493 10.1016/j.cell.2015.11.036 PMC4863988

[12] Johnson TV, Baranov P, Polo AD, Fortune B, Gokoffski KK, Goldberg JL, et al. The Retinal Ganglion Cell Repopulation, Stem Cell Transplantation, & Optic Nerve Regeneration (RReSTORe) Consortium. Ophthalmol Sci. 2023. 10.1016/j.xops.2023.100390. 10.1016/j.xops.2023.100390 PMC10630665 38025164

[13] Fligor CM, Huang K-C, Lavekar SS, VanderWall KB, Meyer JS. Differentiation of retinal organoids from human pluripotent stem cells. Methods Cell Biol. 2020;159:279–302. 32586447 10.1016/bs.mcb.2020.02.005

[14] Wahlin KJ, Cheng J, Jurlina SL, Jones MK, Dash NR, Ogata A, et al. CRISPR Generated SIX6 and POU4F2 Reporters Allow Identification of Brain and Optic Transcriptional Differences in Human PSC-Derived Organoids. Frontiers Cell Dev Biology. 2021;9:764725. 10.3389/fcell.2021.764725 PMC8635054 34869356

[15] Sluch VM, Chamling X, Liu MM, Berlinicke CA, Cheng J, Mitchell KL, et al. Enhanced stem cell differentiation and immunopurification of genome engineered human retinal ganglion cells. Stem Cell Transl Med. 2017;6:1972–86. 10.1002/sctm.17-0059 PMC6430043 29024560

[16] Grigoryan EN. Potential endogenous cell sources for retinal regeneration in vertebrates and humans: progenitor traits and specialization. Biomed. 2020;8:208. 10.3390/biomedicines8070208 PMC7400588 32664635

[17] Yu H, Vu THK, Cho K-S, Guo C, Chen DF. Mobilizing endogenous stem cells for retinal repair. Transl Res. 2014;163:387–98. 24333552 10.1016/j.trsl.2013.11.011 PMC3976683

[18] Capowski EE, Samimi K, Mayerl SJ, Phillips MJ, Pinilla I, Howden SE, et al. Reproducibility and staging of 3D human retinal organoids across multiple pluripotent stem cell lines. Development. 2018;146:dev171686. 10.1242/dev.171686 PMC6340149 30567931

[19] Fligor CM, Lavekar SS, Harkin J, Shields PK, VanderWall KB, Huang K-C, et al. Extension of retinofugal projections in an assembled model of human pluripotent stem cell-derived organoids. Stem Cell Rep. 2021;16:2228–41. 10.1016/j.stemcr.2021.05.009 PMC8452489 34115986

[20] Wahle P, Brancati G, Harmel C, He Z, Gut G, Castillo JS del, et al. Multimodal spatiotemporal phenotyping of human retinal organoid development. Nat Biotechnol. 2023;1–11. 10.1038/s41587-023-01747-2. 10.1038/s41587-023-01747-2 PMC10713453 37156914

[21] Todd L, Hooper MJ, Haugan AK, Finkbeiner C, Jorstad N, Radulovich N, et al. Efficient stimulation of retinal regeneration from Müller glia in adult mice using combinations of proneural bHLH transcription factors. Cell Rep. 2021;37:109857. 34686336 10.1016/j.celrep.2021.109857 PMC8691131

[22] Todd L, Jenkins W, Finkbeiner C, Hooper MJ, Donaldson PC, Pavlou M, et al. Reprogramming Müller glia to regenerate ganglion-like cells in adult mouse retina with developmental transcription factors. Sci Adv. 2022;8:eabq7219. 36417510 10.1126/sciadv.abq7219 PMC9683702

[23] Lenkowski JR, Raymond PA. Müller glia: stem cells for generation and regeneration of retinal neurons in teleost fish. Prog Retin Eye Res. 2014;40:94–123. 24412518 10.1016/j.preteyeres.2013.12.007 PMC3999222

[24] Karl MO, Reh TA. Regenerative medicine for retinal diseases: activating endogenous repair mechanisms. Trends Mol Med. 2010;16:193–202. 20303826 10.1016/j.molmed.2010.02.003 PMC2854262

[25] Bernardos RL, Barthel LK, Meyers JR, Raymond PA. Late-Stage Neuronal Progenitors in the Retina Are Radial Muller Glia That Function as Retinal Stem Cells. J Neurosci. 2007;27:7028–40. 17596452 10.1523/JNEUROSCI.1624-07.2007 PMC6672216

[26] Lahne M, Brecker M, Jones SE, Hyde DR. The regenerating adult zebrafish retina recapitulates developmental fate specification programs. Front Cell Dev Biol. 2021;8:617923. 33598455 10.3389/fcell.2020.617923 PMC7882614

[27] Bringmann A, Iandiev I, Pannicke T, Wurm A, Hollborn M, Wiedemann P, et al. Cellular signaling and factors involved in Müller cell gliosis: Neuroprotective and detrimental effects. Prog Retin Eye Res. 2009;28:423–51. 19660572 10.1016/j.preteyeres.2009.07.001

[28] Zou S, Tian C, Ge S, Hu B. Neurogenesis of retinal ganglion cells is not essential to visual functional recovery after optic nerve injury in adult zebrafish. PLoS One. 2013;8:e57280. 23437359 10.1371/journal.pone.0057280 PMC3577741

[29] Fausett BV, Gumerson JD, Goldman D. The proneural basic helix-loop-helix gene ascl1a is required for retina regeneration. J Neurosci. 2008;28:1109–17. 18234889 10.1523/JNEUROSCI.4853-07.2008 PMC2800945

[30] Jorstad NL, Wilken MS, Grimes WN, Wohl SG, VandenBosch LS, Yoshimatsu T, et al. Stimulation of functional neuronal regeneration from Müller glia in adult mice. Nature. 2017;548:103–7. 28746305 10.1038/nature23283 PMC5991837

[31] Hoang T, Wang J, Boyd P, Wang F, Santiago C, Jiang L, et al. Gene regulatory networks controlling vertebrate retinal regeneration. Science. 2020;370:eabb8598. 10.1126/science.abb8598 PMC7899183 33004674

[32] Johnson TV, Calkins DJ, Fortune B, Goldberg JL, Torre AL, Lamba DA, et al. The importance of unambiguous cell origin determination in neuronal repopulation studies. Iscience. 2023;26:106361. 10.1016/j.isci.2023.106361 PMC10060674 37009209

[33] Blackshaw S, Sanes JR. Turning lead into gold: reprogramming retinal cells to cure blindness. J Clin Invest. 2021;131:e146134. 33529169 10.1172/JCI146134 PMC7843217

[34] Xie Y, Zhou J, Wang LL, Zhang CL, Chen B. New AAV tools fail to detect Neurod1-mediated neuronal conversion of Müller glia and astrocytes in vivo. eBioMedicine. 2023;90:104531. 36947961 10.1016/j.ebiom.2023.104531 PMC10033723

[35] Le N, Appel H, Pannullo N, Hoang T, Blackshaw S. Ectopic insert-dependent neuronal expression of GFAP promoter-driven AAV constructs in adult mouse retina. Frontiers Cell Dev Biology. 2022;10:914386. 10.3389/fcell.2022.914386 PMC9527291 36200040

[36] Xie Y, Chen B. Critical examination of müller glia-derived in vivo neurogenesis in the mouse retina. Front Cell Dev Biol. 2022;10:830382. 35433694 10.3389/fcell.2022.830382 PMC9008276

[37] Nguyen-Ba-Charvet KT, Rebsam A. Neurogenesis and specification of retinal ganglion cells. Int J Mol Sci. 2020;21:451. 31936811 10.3390/ijms21020451 PMC7014133

[38] Bell CM, Zack DJ, Berlinicke CA. Human organoids for the study of retinal development and disease. Annu Rev Vis Sc. 2020;6:91–114. 32936736 10.1146/annurev-vision-121219-081855

[39] Eldred KC, Reh TA. Human retinal model systems: Strengths, weaknesses, and future directions. Dev Biol. 2021;480:114–22. 34529997 10.1016/j.ydbio.2021.09.001

[40] Wang J, He Q, Zhang K, Sun H, Zhang G, Liang H, et al. Quick commitment and efficient reprogramming route of direct induction of retinal ganglion cell-like neurons. Stem Cell Rep. 2020;15:1095–110. 10.1016/j.stemcr.2020.09.008 PMC7663790 33096050

[41] Oliveira-Valença VM, Bosco A, Vetter ML, Silveira MS. On the generation and regeneration of retinal ganglion cells. Frontiers Cell Dev Biology. 2020;8:581136. 10.3389/fcell.2020.581136 PMC7527462 33043015

[42] Xiao D, Deng Q, Guo Y, Huang X, Zou M, Zhong J, et al. Generation of self-organized sensory ganglion organoids and retinal ganglion cells from fibroblasts. Sci Adv. 2020;6:eaaz5858. 32523990 10.1126/sciadv.aaz5858 PMC7259937

[43] Singhal S, Bhatia B, Jayaram H, Becker S, Jones MF, Cottrill PB, et al. Human müller glia with stem cell characteristics differentiate into Retinal Ganglion Cell (RGC) precursors in vitro and partially restore RGC function in vivo following transplantation. Stem Cells Transl Med. 2012;1:188–99. 23197778 10.5966/sctm.2011-0005 PMC3659849

[44] Gill KP, Hewitt AW, Davidson KC, Pébay A, Wong RCB. Methods of retinal ganglion cell differentiation from pluripotent stem cells. Transl Vis Sci Technol. 2014;3:7. 25774327 10.1167/tvst.3.3.7 PMC4356355

[45] Kayama M, Kurokawa MS, Ueda Y, Ueno H, Kumagai Y, Chiba S, et al. Transfection with pax6 gene of mouse embryonic stem cells and subsequent cell cloning induced retinal neuron progenitors, including retinal ganglion cell-like cells, in vitro. Ophthalmic Res. 2010;43:79–91. 19829014 10.1159/000247592

[46] Parameswaran S, Balasubramanian S, Babai N, Qiu F, Eudy JD, Thoreson WB, et al. Induced pluripotent stem cells generate both retinal ganglion cells and photoreceptors: therapeutic implications in degenerative changes in glaucoma and age-related macular degeneration. Stem Cells. 2010;28:695–703. 20166150 10.1002/stem.320

[47] Suzuki N, Shimizu J, Takai K, Arimitsu N, Ueda Y, Takada E, et al. Establishment of retinal progenitor cell clones by transfection with Pax6 gene of mouse induced pluripotent stem (iPS) cells. Neurosci Lett. 2012;509:116–20. 22230895 10.1016/j.neulet.2011.12.055

[48] Aoki H, Hara A, Niwa M, Motohashi T, Suzuki T, Kunisada T. Transplantation of cells from eye-like structures differentiated from embryonic stem cells in vitro and in vivo regeneration of retinal ganglion-like cells. Graefe’s Arch Clin Exp Ophthalmol. 2008;246:255–65. 18004585 10.1007/s00417-007-0710-6

[49] Meyer JS, Howden SE, Wallace KA, Verhoeven AD, Wright LS, Capowski EE, et al. Optic vesicle-like structures derived from human pluripotent stem cells facilitate a customized approach to retinal disease treatment. Stem Cells. 2011;29:1206–18. 21678528 10.1002/stem.674 PMC3412675

[50] Riazifar H, Jia Y, Chen J, Lynch G, Huang T. Chemically induced specification of retinal ganglion cells from human embryonic and induced pluripotent stem cells. Stem Cells Transl Med. 2014;3:424–32. 24493857 10.5966/sctm.2013-0147 PMC3973714

[51] Sridhar A, Steward MM, Meyer JS. Nonxenogeneic growth and retinal differentiation of human induced pluripotent stem cells. STEM CELLS Transl Med. 2013;2:255–64. 23512959 10.5966/sctm.2012-0101 PMC3659835

[52] Osakada F, Jin Z-B, Hirami Y, Ikeda H, Danjyo T, Watanabe K, et al. In vitro differentiation of retinal cells from human pluripotent stem cells by small-molecule induction. J Cell Sci. 2009;122:3169–79. 19671662 10.1242/jcs.050393

[53] Hirami Y, Osakada F, Takahashi K, Okita K, Yamanaka S, Ikeda H, et al. Generation of retinal cells from mouse and human induced pluripotent stem cells. Neurosci Lett. 2009;458:126–31. 19379795 10.1016/j.neulet.2009.04.035

[54] Oswald J, Kegeles E, Minelli T, Volchkov P, Baranov P. Transplantation of miPSC/mESC-derived retinal ganglion cells into healthy and glaucomatous retinas. Mol Ther - Methods Clin Dev. 2021;21:180–98. 33816648 10.1016/j.omtm.2021.03.004 PMC7994731

[55] Langer KB, Ohlemacher SK, Phillips MJ, Fligor CM, Jiang P, Gamm DM, et al. Retinal Ganglion Cell Diversity and Subtype Specification from Human Pluripotent Stem Cells. Stem Cell Rep. 2018;10:1282–93. 10.1016/j.stemcr.2018.02.010 PMC5998302 29576537

[56] Amin D, Kuwajima T. Differential retinal ganglion cell vulnerability, a critical clue for the identification of neuroprotective genes in glaucoma. Front Ophthalmol. 2022;2:905352. 10.3389/fopht.2022.905352 PMC11182220 38983528

[57] Achberger K, Probst C, Haderspeck J, Bolz S, Rogal J, Chuchuy J, et al. Merging organoid and organ-on-a-chip technology to generate complex multi-layer tissue models in a human Retina-on-a-Chip platform. eLife. 2019;8:e46188. 10.7554/eLife.46188 PMC6777939 31451149

[58] Achberger K, Cipriano M, Düchs MJ, Schön C, Michelfelder S, Stierstorfer B, et al. Human stem cell-based retina on chip as new translational model for validation of AAV retinal gene therapy vectors. Stem Cell Rep. 2021;16:2242–56. 10.1016/j.stemcr.2021.08.008 PMC8452599 34525384

[59] Sridhar A, Hoshino A, Finkbeiner CR, Chitsazan A, Dai L, Haugan AK, et al. Single-cell transcriptomic comparison of human fetal retina, hPSC-derived retinal organoids, and long-term retinal cultures. Cell Rep. 2020;30:1644–1659.e4. 32023475 10.1016/j.celrep.2020.01.007 PMC7901645

[60] Zhong X, Gutierrez C, Xue T, Hampton C, Vergara MN, Cao L-H, et al. Generation of three-dimensional retinal tissue with functional photoreceptors from human iPSCs. Nat Commun. 2014;5:4047. 24915161 10.1038/ncomms5047 PMC4370190

[61] Brodie-Kommit J, Clark BS, Shi Q, Shiau F, Kim DW, Langel J, et al. Atoh7-independent specification of retinal ganglion cell identity. Sci Adv. 2021;7:eabe4983. 33712461 10.1126/sciadv.abe4983 PMC7954457

[62] Wu F, Bard JE, Kann J, Yergeau D, Sapkota D, Ge Y, et al. Single cell transcriptomics reveals lineage trajectory of retinal ganglion cells in wild-type and Atoh7-null retinas. Nat Commun. 2021;12:1465. 33674582 10.1038/s41467-021-21704-4 PMC7935890

[63] Gan L, Wang SW, Huang Z, Klein WH. POU domain factor Brn-3b is essential for retinal ganglion cell differentiation and survival but not for initial cell fate specification. Dev Biol. 1999;210:469–80. 10357904 10.1006/dbio.1999.9280

[64] Elshatory Y, Deng M, Xie X, Gan L. Expression of the LIM-homeodomain protein Isl1 in the developing and mature mouse retina. J Comp Neurol. 2007;503:182–97. 17480014 10.1002/cne.21390 PMC2950632

[65] Fernández-Nogales M, López-Cascales MT, Murcia-Belmonte V, Escalante A, Fernández-Albert J, Muñoz-Viana R, et al. Multiomic analysis of neurons with divergent projection patterns identifies novel regulators of axon pathfinding. Adv Sci. 2022;9:2200615. 10.1002/advs.202200615 PMC9561852 35988153

[66] Wu F, Kaczynski TJ, Sethuramanujam S, Li R, Jain V, Slaughter M, et al. Two transcription factors, Pou4f2 and Isl1, are sufficient to specify the retinal ganglion cell fate. Proc Natl Acad Sci. 2015;112:E1559–68. 25775587 10.1073/pnas.1421535112 PMC4386335

[67] Zhang X-M, Yang X-J. Regulation of retinal ganglion cell production by Sonic hedgehog. Development. 2001;128:943–57. 11222148 10.1242/dev.128.6.943 PMC7048390

[68] Osborne A, Khatib TZ, Songra L, Barber AC, Hall K, Kong GYX, et al. Neuroprotection of retinal ganglion cells by a novel gene therapy construct that achieves sustained enhancement of brain-derived neurotrophic factor/tropomyosin-related kinase receptor-B signaling. Cell Death Dis. 2018;9:1007. 30258047 10.1038/s41419-018-1041-8 PMC6158290

[69] Lambuk L, Lazaldin MAM, Ahmad S, Iezhitsa I, Agarwal R, Uskoković V, et al. Brain-derived neurotrophic factor-mediated neuroprotection in glaucoma: a review of current state of the art. Front Pharmacol. 2022;13:875662. 35668928 10.3389/fphar.2022.875662 PMC9163364

[70] Vecino E, Heller JP, Veiga-Crespo P, Martin KR, Fawcett JW. Influence of extracellular matrix components on the expression of integrins and regeneration of adult retinal ganglion cells. PLoS One. 2015;10:e0125250. 26018803 10.1371/journal.pone.0125250 PMC4446304

[71] Bao Z-Z. Intraretinal projection of retinal ganglion cell axons as a model system for studying axon navigation. Brain Res. 2008;1192:165–77. 17320832 10.1016/j.brainres.2007.01.116 PMC2267003

[72] Randlett O, Poggi L, Zolessi FR, Harris WA. The oriented emergence of axons from retinal ganglion cells is directed by laminin contact in vivo. Neuron. 2011;70:266–80. 21521613 10.1016/j.neuron.2011.03.013 PMC3087191

[73] Cheng J, Li S, Berlinicke C, Zhang P, Chang X, Welsbie DS, et al. Histone deacetylase and myosin inhibitors promote neurite outgrowth in injured retinal ganglion cells derived from human pluripotent stem cells. Investig Ophthalmol Vis Sci. 2022;63:2445-F0389-2445-F0389.

[74] Schwechter BR, Millet LE, Levin LA. Histone deacetylase inhibition-mediated differentiation of RGC-5 cells and interaction with survival. Investig Opthalmol Vis Sci. 2007;48:2845. 10.1167/iovs.06-1364 PMC2206540 17525221

[75] Lu Y, Brommer B, Tian X, Krishnan A, Meer M, Wang C, et al. Reprogramming to recover youthful epigenetic information and restore vision. Nature. 2020;588:124–9. 33268865 10.1038/s41586-020-2975-4 PMC7752134

[76] Rheaume BA, Xing J, Lukomska A, Theune WC, Damania A, Sjogren G, et al. PTEN inhibition dedifferentiates long-distance axon-regenerating intrinsically photosensitive retinal ganglion cells and upregulates mitochondria-associated DYNLT1A and LARS2. Development. 2023;150:dev201644. 37039265 10.1242/dev.201644 PMC10163351

[77] Mertens J, Paquola ACM, Ku M, Hatch E, Böhnke L, Ladjevardi S, et al. Directly reprogrammed human neurons retain aging-associated transcriptomic signatures and reveal age-related nucleocytoplasmic defects. Cell Stem Cell. 2015;17:705–18. 26456686 10.1016/j.stem.2015.09.001 PMC5929130

[78] Sanes JR, Masland RH. The types of retinal ganglion cells: current status and implications for neuronal classification. Annu Rev Neurosci. 2015;38:1–26. 25897874 10.1146/annurev-neuro-071714-034120

[79] Goetz J, Jessen ZF, Jacobi A, Mani A, Cooler S, Greer D, et al. Unified classification of mouse retinal ganglion cells using function, morphology, and gene expression. Cell Rep. 2022;40:111040. 35830791 10.1016/j.celrep.2022.111040 PMC9364428

[80] Kölsch Y, Hahn J, Sappington A, Stemmer M, Fernandes AM, Helmbrecht TO, et al. Molecular classification of zebrafish retinal ganglion cells links genes to cell types to behavior. Neuron. 2021;109:645–662.e9. 33357413 10.1016/j.neuron.2020.12.003 PMC7897282

[81] Tran NM, Shekhar K, Whitney IE, Jacobi A, Benhar I, Hong G, et al. Single-cell profiles of retinal ganglion cells differing in resilience to injury reveal neuroprotective genes. Neuron. 2019;104:1039–1055.e12. 31784286 10.1016/j.neuron.2019.11.006 PMC6923571

[82] Yan W, Peng Y-R, van Zyl T, Regev A, Shekhar K, Juric D, et al. Cell atlas of the human fovea and peripheral retina. Sci Rep-uk. 2020;10:9802. 10.1038/s41598-020-66092-9 PMC7299956 32555229

[83] Ramos OM, Barker D, Ferrier DEK. Ghost loci imply hox and parahox existence in the last common ancestor of animals. Curr Biol. 2012;22:1951–6. 23022064 10.1016/j.cub.2012.08.023

[84] Jeon CJ, Strettoi E, Masland RH. The major cell populations of the mouse retina. J Neurosci. 1998;18:8936–46. 9786999 10.1523/JNEUROSCI.18-21-08936.1998 PMC6793518

[85] Masland RH. The neuronal organization of the retina. Neuron. 2012;76:266–80. 23083731 10.1016/j.neuron.2012.10.002 PMC3714606

[86] Lipovsek M, Bardy C, Cadwell CR, Hadley K, Kobak D, Tripathy SJ. Patch-seq: past, present, and future. J Neurosci. 2021;41:937–46. 33431632 10.1523/JNEUROSCI.1653-20.2020 PMC7880286

[87] Williams CG, Lee HJ, Asatsuma T, Vento-Tormo R, Haque A. An introduction to spatial transcriptomics for biomedical research. Genome Med. 2022;14:68. 35761361 10.1186/s13073-022-01075-1 PMC9238181

[88] Zhang M, Eichhorn SW, Zingg B, Yao Z, Cotter K, Zeng H, et al. Spatially resolved cell atlas of the mouse primary motor cortex by MERFISH. Nature. 2021;598:137–43. 34616063 10.1038/s41586-021-03705-x PMC8494645

[89] Choi J, Li J, Ferdous S, Liang Q, Moffitt JR, Chen R. Spatial organization of the mouse retina at single cell resolution. Biorxiv. 2022;2022.12.04.518972.

[90] Havens SJ, Ghate DA, Gulati V. Neuroimmune Pharmacology. 2016. p. 533–52.

[91] Harada T, Harada C, Parada LF. Molecular regulation of visual system development: more than meets the eye. Genes Dev. 2007;21:367–78. 17322396 10.1101/gad.1504307

[92] Quigley HA, Dunkelberger GR, Green WR. Chronic human glaucoma causing selectively greater loss of large optic nerve fibers. Ophthalmology. 1988;95:357–63. 3174003 10.1016/s0161-6420(88)33176-3

[93] Quigley HA, Dunkelberger GR, Green WR. Retinal ganglion cell atrophy correlated with automated perimetry in human eyes with glaucoma. Am J Ophthalmol. 1989;107:453–64. 2712129 10.1016/0002-9394(89)90488-1

[94] Howe JW, Mitchell KW. Electrophysiologically determined contrast sensitivity in patients with ocular hypertension and chronic glaucoma. Doc Ophthalmol. 1992;80:31–41. 1505337 10.1007/BF00161229

[95] Chaturvedi N, Hedley-Whyte ET, Dreyer EB. Lateral geniculate nucleus in glaucoma. Am J Ophthalmol. 1993;116:182–8. 8352303 10.1016/s0002-9394(14)71283-8

[96] Anderson RS, O’Brien C. Psychophysical evidence for a selective loss of m ganglion cells in glaucoma. Vision Res. 1997;37:1079–83. 9196726 10.1016/s0042-6989(96)00260-x

[97] Klistorner AI, Graham SL. Early magnocellular loss in glaucoma demonstrated using the pseudorandomly stimulated flash visual evoked potential. J Glaucoma. 1999;8:140–8. 10209732

[98] Kerrigan-Baumrind LA, Quigley HA, Pease ME, Kerrigan DF, Mitchell RS. Number of ganglion cells in glaucoma eyes compared with threshold visual field tests in the same persons. Invest Ophth Vis Sci. 2000;41:741–8. 10711689

[99] Sun H, Swanson WH, Arvidson B, Dul MW. Assessment of contrast gain signature in inferred magnocellular and parvocellular pathways in patients with glaucoma. Vision Res. 2008;48:2633–41. 18501947 10.1016/j.visres.2008.04.008 PMC2825154

[100] Zhang P, Wen W, Sun X, He S. Selective reduction of fMRI responses to transient achromatic stimuli in the magnocellular layers of the LGN and the superficial layer of the SC of early glaucoma patients. Hum Brain Mapp. 2016;37:558–69. 26526339 10.1002/hbm.23049 PMC6867378

[101] Casson EJ, Johnson CA, Shapiro LR. Longitudinal comparison of temporal-modulation perimetry with white-on-white and blue-on-yellow perimetry in ocular hypertension and early glaucoma. J Opt Soc Am. 1993;10:1792. 10.1364/josaa.10.001792 8350162

[102] Sample PA, Bosworth CF, Weinreb RN. Short-wavelength automated perimetry and motion automated perimetry in patients with glaucoma. Arch Ophthalmol-chic. 1997;115:1129–33. 10.1001/archopht.1997.01100160299006 9298053

[103] Sample PA, Bosworth CF, Blumenthal EZ, Girkin C, Weinreb RN. Visual function-specific perimetry for indirect comparison of different ganglion cell populations in glaucoma. Invest Ophth Vis Sci. 2000;41:1783–90. 10845599

[104] Landers JA, Goldberg I, Graham SL. Detection of early visual field loss in glaucoma using frequency-doubling perimetry and short-wavelength automated perimetry. Arch Ophthalmol-chic. 2003;121:1705–10. 10.1001/archopht.121.12.1705 14662589

[105] McKendrick AM, Badcock DR, Morgan WH. Psychophysical measurement of neural adaptation abnormalities in magnocellular and parvocellular pathways in glaucoma. Investig Opthalmol Vis Sci. 2004;45:1846. 10.1167/iovs.03-1225 15161849

[106] McKendrick AM, Sampson GP, Walland MJ, Badcock DR. Contrast sensitivity changes due to glaucoma and normal aging: low-spatial-frequency losses in both magnocellular and parvocellular pathways. Investig Opthalmol Vis Sci. 2007;48:2115. 10.1167/iovs.06-1208 17460269

[107] Battista J, Badcock DR, McKendrick AM. Spatial summation properties for magnocellular and parvocellular pathways in glaucoma. Investig Opthalmol Vis Sci. 2009;50:1221. 10.1167/iovs.08-2517 18936145

[108] Mikelberg FS, Yidegiligne HM, Schulzer M. Optic nerve axon count and won diameter in patients with ocular hypertension and normal visual fields. Ophthalmology. 1995;102:342–8. 7862423 10.1016/s0161-6420(95)31019-6

[109] Quigley HA, Sanchez RM, Dunkelberger GR, L’Hernault NL, Baginski TA. Chronic glaucoma selectively damages large optic nerve fibers. Invest Ophth Vis Sci. 1987;28:913–20. 3583630

[110] Glovinsky Y, Quigley HA, Dunkelberger GR. Retinal ganglion cell loss is size dependent in experimental glaucoma. Invest Ophth Vis Sci. 1991;32:484–91. 2001923

[111] Dandona L, Hendrickson A, Quigley HA. Selective effects of experimental glaucoma on axonal transport by retinal ganglion cells to the dorsal lateral geniculate nucleus. Invest Ophth Vis Sci. 1991;32:1593–9. 1707861

[112] Glovinsky Y, Quigley HA, Pease ME. Foveal ganglion cell loss is size dependent in experimental glaucoma. Invest Ophth Vis Sci. 1993;34:395–400. 8440594

[113] Vickers JC, Schumer RA, Podos SM, Wang RF, Riederer BM, Morrison JH. Differential vulnerability of neurochemically identified subpopulations of retinal neurons in a monkey model of glaucoma. Brain Res. 1995;680:23–35. 7663981 10.1016/0006-8993(95)00211-8

[114] Weber AJ, Kaufman PL, Hubbard WC. Morphology of single ganglion cells in the glaucomatous primate retina. Invest Ophth Vis Sci. 1998;39:2304–20. 9804139

[115] Weber AJ, Chen H, Hubbard WC, Kaufman PL. Experimental glaucoma and cell size, density, and number in the primate lateral geniculate nucleus. Invest Ophth Vis Sci. 2000;41:1370–9. 10798652

[116] Ito Y, Shimazawa M, Chen Y-N, Tsuruma K, Yamashima T, Araie M, et al. Morphological changes in the visual pathway induced by experimental glaucoma in Japanese monkeys. Exp Eye Res. 2009;89:246–55. 19341728 10.1016/j.exer.2009.03.013

[117] Ruiz-Ederra J, García M, Hernández M, Urcola H, Hernández-Barbáchano E, Araiz J, et al. The pig eye as a novel model of glaucoma. Exp Eye Res. 2005;81:561–9. 15949799 10.1016/j.exer.2005.03.014

[118] Kalloniatis M, Harwertah RS, Smith EL, DeSantis L. Colour vision anomalies following experimental glaucoma in monkeys. Ophthal Physl Opt. 1993;13:56–67. 10.1111/j.1475-1313.1993.tb00427.x 8510949

[119] Smith E, Chino Y, Harwerth R, Ridder W, Crawford MJ, DeSantis L. Retinal inputs to the monkey’s lateral geniculate nucleus in experimental glaucoma. Clin Vis Sci. 1993;8:113–39.

[120] Vickers J, Hof P, Schumer R, Wang R, Podos S, Morrison J. Magnocellular and parvocellular visual pathways are both affected in a macaque monkey model of glaucoma. Aust Nz J Ophthalmol. 1997;25:239–43. 10.1111/j.1442-9071.1997.tb01400.x 9296301

[121] Yücel YH, Zhang Q, Gupta N, Kaufman PL, Weinreb RN. Loss of neurons in magnocellular and parvocellular layers of the lateral geniculate nucleus in glaucoma. Arch Ophthalmol-chic. 2000;118:378–84. 10.1001/archopht.118.3.378 10721961

[122] Morgan JE, Uchida H, Caprioli J. Retinal ganglion cell death in experimental glaucoma. Brit J Ophthalmol. 2000;84:303. 10684843 10.1136/bjo.84.3.303 PMC1723413

[123] Crawford ML, Harwerth RS, Smith EL, Shen F, Carter-Dawson L. Glaucoma in primates: cytochrome oxidase reactivity in parvo- and magnocellular pathways. Invest Ophth Vis Sci. 2000;41:1791–802. 10845600

[124] Yücel YH, Zhang Q, Weinreb RN, Kaufman PL, Gupta N. Effects of retinal ganglion cell loss on magno-, parvo-, koniocellular pathways in the lateral geniculate nucleus and visual cortex in glaucoma. Prog Retin Eye Res. 2003;22:465–81. 12742392 10.1016/s1350-9462(03)00026-0

[125] Shou T, Liu J, Wang W, Zhou Y, Zhao K. Differential dendritic shrinkage of α and β retinal ganglion cells in cats with chronic glaucoma. Investig Opthalmol Vis Sci. 2003;44:3005. 10.1167/iovs.02-0620 12824245

[126] Shou TD, Zhou YF. Y cells in the cat retina are more tolerant than X cells to brief elevation of IOP. Invest Ophth Vis Sci. 1989;30:2093–8. 2793352

[127] Zhou Y, Wang W, Ren B, Shou T. Receptive field properties of cat retinal ganglion cells during short-term IOP elevation. Invest Ophth Vis Sci. 1994;35:2758–64. 8188469

[128] Watanabe M, Fukuda Y. Survival and axonal regeneration of retinal ganglion cells in adult cats. Prog Retin Eye Res. 2002;21:529–53. 12433376 10.1016/s1350-9462(02)00037-x

[129] Watanabe M, Sawai H, Fukuda Y. Number and dendritic morphology of retinal ganglion cells that survived after axotomy in adult cats. J Neurobiol. 1995;27:189–203. 7658200 10.1002/neu.480270206

[130] Vorwerk CK, Kreutz MR, Böckers TM, Brosz M, Dreyer EB, Sabel BA. Susceptibility of retinal ganglion cells to excitotoxicity depends on soma size and retinal eccentricity. Curr Eye Res. 1999;19:59–65. 10415458 10.1076/ceyr.19.1.59.5336

[131] Li RS, Chen B-Y, Tay DK, Chan HHL, Pu M-L, So K-F. Melanopsin-expressing retinal ganglion cells are more injury-resistant in a chronic ocular hypertension model. Investig Opthalmol Vis Sci. 2006;47:2951. 10.1167/iovs.05-1295 16799038

[132] Li SY, Yau SY, Chen BY, Tay DK, Lee VWH, Pu ML, et al. Enhanced survival of melanopsin-expressing retinal ganglion cells after injury is associated with the PI3 K/Akt pathway. Cell Mol Neurobiol. 2008;28:1095–107. 18512147 10.1007/s10571-008-9286-x PMC11514987

[133] de Zavalía N, Plano SA, Fernandez DC, Lanzani MF, Salido E, Belforte N, et al. Effect of experimental glaucoma on the non-image forming visual system. J Neurochem. 2011;117:904–14. 21446997 10.1111/j.1471-4159.2011.07260.x

[134] Müller LP de S, Sargoy A, Rodriguez AR, Brecha NC. Melanopsin Ganglion Cells Are the Most Resistant Retinal Ganglion Cell Type to Axonal Injury in the Rat Retina. PLos One. 2014;9:e93274. 10.1371/journal.pone.0093274 PMC3966869 24671191

[135] Nadal-Nicolás FM, Sobrado-Calvo P, Jiménez-López M, Vidal-Sanz M, Agudo-Barriuso M. Long-term effect of optic nerve axotomy on the retinal ganglion cell layer. Investig Opthalmol Vis Sci. 2015;56:6095. 10.1167/iovs.15-17195 26393669

[136] Rovere G, Nadal-Nicolás FM, Wang J, Bernal-Garro JM, García-Carrillo N, Villegas-Pérez MP, et al. Melanopsin-containing or non-melanopsin–containing retinal ganglion cells response to acute ocular hypertension with or without brain-derived neurotrophic factor neuroprotection. Investig Opthalmol Vis Sci. 2016;57:6652. 10.1167/iovs.16-20146 27930778

[137] Vidal-Villegas B, Pierdomenico JD, de Imperial-Ollero JAM, Ortín-Martínez A, Nadal-Nicolás FM, Bernal-Garro JM, et al. Melanopsin+RGCs Are fully Resistant to NMDA-Induced Excitotoxicity. Int J Mol Sci. 2019;20:3012. 31226772 10.3390/ijms20123012 PMC6627747

[138] VanderWall KB, Lu B, Alfaro JS, Allsop AR, Carr AS, Wang S, et al. Differential susceptibility of retinal ganglion cell subtypes in acute and chronic models of injury and disease. Sci Rep-uk. 2020;10:17359. 10.1038/s41598-020-71460-6 PMC7566630 33060618

[139] Robinson GA, Madison RD. Axotomized mouse retinal ganglion cells containing melanopsin show enhanced survival, but not enhanced axon regrowth into a peripheral nerve graft. Vision Res. 2004;44:2667–74. 15358062 10.1016/j.visres.2004.06.010

[140] Yang N, Young BK, Wang P, Tian N. The susceptibility of retinal ganglion cells to optic nerve injury is type specific. Cells. 2020;9:677. 32164319 10.3390/cells9030677 PMC7140711

[141] Daniel S, Meyer KJ, Clark AF, Anderson MG, McDowell CM. Effect of ocular hypertension on the pattern of retinal ganglion cell subtype loss in a mouse model of early-onset glaucoma. Exp Eye Res. 2019;185:107703. 31211954 10.1016/j.exer.2019.107703 PMC7430001

[142] Christensen I, Lu B, Yang N, Huang K, Wang P, Tian N. The susceptibility of retinal ganglion cells to glutamatergic excitotoxicity is type-specific. Front Neurosci-switz. 2019;13:219. 10.3389/fnins.2019.00219 PMC6429039 30930737

[143] Wang S, Gu D, Zhang P, Chen J, Li Y, Xiao H, et al. Melanopsin-expressing retinal ganglion cells are relatively resistant to excitotoxicity induced by N-methyl-d-aspartate. Neurosci Lett. 2018;662:368–73. 29102785 10.1016/j.neulet.2017.10.055

[144] Daniel S, Clark A, McDowell C. Subtype-specific response of retinal ganglion cells to optic nerve crush. Cell Death Discov. 2018;4:67. 30062056 10.1038/s41420-018-0069-y PMC6054657

[145] Sabharwal J, Seilheimer RL, Tao X, Cowan CS, Frankfort BJ, Wu SM. Elevated IOP alters the space–time profiles in the center and surround of both ON and OFF RGCs in mouse. Proc National Acad Sci. 2017;114:8859–64. 10.1073/pnas.1706994114 PMC5565456 28760976

[146] Puyang Z, Gong HQ, He SG, Troy JB, Liu X, Liang PJ. Different functional susceptibilities of mouse retinal ganglion cell subtypes to optic nerve crush injury. Exp Eye Res. 2017;162:97–103. 28629926 10.1016/j.exer.2017.06.014

[147] Ou Y, Jo RE, Ullian EM, Wong ROL, Santina LD. Selective vulnerability of specific retinal ganglion cell types and synapses after transient ocular hypertension. J Neurosci. 2016;36:9240–52. 27581463 10.1523/JNEUROSCI.0940-16.2016 PMC5005727

[148] Chen H, Zhao Y, Liu M, Feng L, Puyang Z, Yi J, et al. Progressive degeneration of retinal and superior collicular functions in mice with sustained ocular hypertension. Investig Opthalmol Vis Sci. 2015;56:1971. 10.1167/iovs.14-15691 PMC4365983 25722210

[149] Duan X, Qiao M, Bei F, Kim I-J, He Z, Sanes JR. Subtype-specific regeneration of retinal ganglion cells following axotomy: effects of osteopontin and mTOR signaling. Neuron. 2015;85:1244–56. 25754821 10.1016/j.neuron.2015.02.017 PMC4391013

[150] El-Danaf RN, Huberman AD. Characteristic patterns of dendritic remodeling in early-stage glaucoma: evidence from genetically identified retinal ganglion cell types. J Neurosci. 2015;35:2329–43. 25673829 10.1523/JNEUROSCI.1419-14.2015 PMC6605614

[151] Perganta G, Barnard AR, Katti C, Vachtsevanos A, Douglas RH, MacLaren RE, et al. Non-image-forming light driven functions are preserved in a mouse model of autosomal dominant optic atrophy. PLoS One. 2013;8:e56350. 23409176 10.1371/journal.pone.0056350 PMC3569441

[152] Zhang Q, Vuong H, Huang X, Wang Y, Brecha NC, Pu M, et al. Melanopsin-expressing retinal ganglion cell loss and behavioral analysis in the Thy1-CFP-DBA/2J mouse model of glaucoma. Sci China Life Sci. 2013;56:720–30. 23729182 10.1007/s11427-013-4493-1 PMC3804076

[153] Feng L, Zhao Y, Yoshida M, Chen H, Yang JF, Kim TS, et al. Sustained ocular hypertension induces dendritic degeneration of mouse retinal ganglion cells that depends on cell type and location. Investig Opthalmol Vis Sci. 2013;54:1106. 10.1167/iovs.12-10791 PMC3567754 23322576

[154] Santina LD, Inman DM, Lupien CB, Horner PJ, Wong ROL. Differential progression of structural and functional alterations in distinct retinal ganglion cell types in a mouse model of glaucoma. J Neurosci. 2013;33:17444–57. 24174678 10.1523/JNEUROSCI.5461-12.2013 PMC3812509

[155] DeParis S, Caprara C, Grimm C. Intrinsically photosensitive retinal ganglion cells are resistant to N-methyl-D-aspartic acid excitotoxicity. Mol Vis. 2012;18:2814–27. 23233784 PMC3519378

[156] Leung CK, Weinreb RN, Li ZW, Liu S, Lindsey JD, Choi N, et al. Long-term in vivo imaging and measurement of dendritic shrinkage of retinal ganglion cells. Investig Opthalmol Vis Sci. 2011;52:1539. 10.1167/iovs.10-6012 21245394

[157] Jakobs TC, Libby RT, Ben Y, John SWM, Masland RH. Retinal ganglion cell degeneration is topological but not cell type specific in DBA/2J mice. J Cell Biology. 2005;171:313–25. 10.1083/jcb.200506099 PMC2171185 16247030

[158] Risner ML, Pasini S, Cooper ML, Lambert WS, Calkins DJ. Axogenic mechanism enhances retinal ganglion cell excitability during early progression in glaucoma. Proc National Acad Sci. 2018;115:E2393–402. 10.1073/pnas.1714888115 PMC5877940 29463759

[159] Urcola JH, Hernández M, Vecino E. Three experimental glaucoma models in rats: Comparison of the effects of intraocular pressure elevation on retinal ganglion cell size and death. Exp Eye Res. 2006;83:429–37. 16682027 10.1016/j.exer.2006.01.025

[160] Vecino E, Urcola H, Bayon A, Sharma SC. Glaucoma, Methods and Protocols. Methods Mol Biol. 2017;1695:41–8. 10.1007/978-1-4939-7407-8_4 29190016

[161] Laquis S, Chaudhary P, Sharma SC. The patterns of retinal ganglion cell death in hypertensive eyes. Brain Res. 1998;784:100–4. 9518569 10.1016/s0006-8993(97)01189-x

[162] Ahmed FAKM, Chaudhary P, Sharma SC. Effects of increased intraocular pressure on rat retinal ganglion cells. Int J Dev Neurosci. 2001;19:209–18. 11255034 10.1016/s0736-5748(00)00073-3

[163] Santina LD, Ou Y. Who’s lost first? Susceptibility of retinal ganglion cell types in experimental glaucoma. Exp Eye Res. 2017;158:43–50. 27319294 10.1016/j.exer.2016.06.006 PMC5161723

[164] Wareham LK, Risner ML, Calkins DJ. Protect, repair, and regenerate: towards restoring vision in glaucoma. Curr Ophthalmol Reports. 2020;8:301–10. 10.1007/s40135-020-00259-5 PMC7686214 33269115

[165] Pollard DA. Quantitative Trait Loci (QTL), methods and protocols. Methods Mol Biol. 2012;871:31–9. 22565831 10.1007/978-1-61779-785-9_3

[166] Rao A, Padhy D, Pal A, Roy AK. Visual function tests for glaucoma practice - What is relevant? Indian J Ophthalmol. 2022;70:749–58. 35225508 10.4103/ijo.IJO_1390_21 PMC9114550

[167] Teotia P, Niu M, Ahmad I. Mapping developmental trajectories and subtype diversity of normal and glaucomatous human retinal ganglion cells by single-cell transcriptome analysis. Stem Cells. 2020;38:1279–91. 32557945 10.1002/stem.3238 PMC7586941

[168] Qi X-R, Verwer RWH, Bao A-M, Balesar RA, Luchetti S, Zhou J-N, et al. Human brain slice culture: a useful tool to study brain disorders and potential therapeutic compounds. Neurosci Bull. 2019;35:244–52. 30604279 10.1007/s12264-018-0328-1 PMC6426918

[169] McKinnon SJ, Schlamp CL, Nickells RW. Mouse models of retinal ganglion cell death and glaucoma. Exp Eye Res. 2009;88:816–24. 19105954 10.1016/j.exer.2008.12.002 PMC3056071

[170] Johnson TV, Tomarev SI. Rodent models of glaucoma. Brain Res Bull. 2010;81:349–58. 19379796 10.1016/j.brainresbull.2009.04.004 PMC2830899

[171] Kimura A, Noro T, Harada T. Role of animal models in glaucoma research. Neural Regen Res. 2020;15:1257–8. 31960810 10.4103/1673-5374.272578 PMC7047796

[172] Niwa M, Aoki H, Hirata A, Tomita H, Green PG, Hara A. Retinal cell degeneration in animal models. Int J Mol Sci. 2016;17:110. 26784179 10.3390/ijms17010110 PMC4730351

[173] Wu G, Harp CP, Shindler K. Optic neuritis: a model for the immuno-pathogenesis of central nervous system inflammatory demyelinating diseases. Curr Immunol Rev. 2015;11:85–92. 29399010 10.2174/1573395511666150707181644 PMC5791743

[174] Tao W, Dvoriantchikova G, Tse BC, Pappas S, Chou TH, Tapia M, et al. A novel mouse model of traumatic optic neuropathy using external ultrasound energy to achieve focal. Indirect Optic Nerve Injury Sci Rep-uk. 2017;7:11779. 10.1038/s41598-017-12225-6 PMC5603527 28924145

[175] Bernstein SL, Miller NR. Ischemic optic neuropathies and their models: disease comparisons, model strengths and weaknesses. Jpn J Ophthalmol. 2015;59:135–47. 25690987 10.1007/s10384-015-0373-5 PMC4556370

[176] Manogaran P, Samardzija M, Schad AN, Wicki CA, Walker-Egger C, Rudin M, et al. Correction to: retinal pathology in experimental optic neuritis is characterized by retrograde degeneration and gliosis. Acta Neuropathologica Commun. 2019;7:157. 10.1186/s40478-019-0825-0 PMC6798424 31627732

[177] Orgül S, Cioffi GA, Wilson DJ, Bacon DR, Buskirk EMV. An endothelin-1 induced model of optic nerve ischemia in the rabbit. Invest Ophth Vis Sci. 1996;37:1860–9. 8759355

[178] Hartsock MJ, Cho H, Wu L, Chen W-J, Gong J, Duh EJ. A Mouse Model of Retinal Ischemia-Reperfusion Injury Through Elevation of Intraocular Pressure. J Vis Exp. 2016;14:e54065. 10.3791/54065 PMC5091361 27501124

[179] Borrás T. Gene therapy strategies in glaucoma and application for steroid-induced hypertension. Saudi J Ophthalmol. 2011;25:353–62. 23960949 10.1016/j.sjopt.2011.07.005 PMC3729681

[180] Chandra A, Smith J, Wang BZ, Kurniawan E, Kam J, Sandhu SS, et al. Post-Vitrectomy Endophthalmitis in Victoria. Australia Asia-pacific J Ophthalmol. 2017;6:80–93. 10.22608/APO.2016126 28161912

[181] Pang I-H, Clark AF. Inducible rodent models of glaucoma. Prog Retin Eye Res. 2020;75:100799. 31557521 10.1016/j.preteyeres.2019.100799 PMC7085984

[182] Zhang J, Li L, Huang H, Fang F, Webber HC, Zhuang P, et al. Silicone oil-induced ocular hypertension and glaucomatous neurodegeneration in mouse. Elife. 2019;8:e45881. 31090540 10.7554/eLife.45881 PMC6533060

[183] Shareef SR, Garcia-Valenzuela E, Salierno A, Walsh J, Sharma SC. Chronic ocular hypertension following episcleral venous occlusion in rats. Exp Eye Res. 1995;61:379–82. 7556500 10.1016/s0014-4835(05)80131-9

[184] Jayaram H. Intraocular pressure reduction in glaucoma: Does every mmHg count? Taiwan J Ophthalmol. 2020;10:255–8. 33437597 10.4103/tjo.tjo_63_20 PMC7787090

[185] Killer H, Pircher A. Normal tension glaucoma: review of current understanding and mechanisms of the pathogenesis. Eye. 2018;32:924–30. 29456252 10.1038/s41433-018-0042-2 PMC5944657

[186] Swarup G, Sayyad Z. Altered Functions and Interactions of Glaucoma-Associated Mutants of Optineurin. Front Immunol. 2018;9:1287. 29951055 10.3389/fimmu.2018.01287 PMC6008547

[187] Ghinia MG, Novelli E, Sajgo S, Badea TC, Strettoi E. Brn3a and Brn3b knockout mice display unvaried retinal fine structure despite major morphological and numerical alterations of ganglion cells. J Comp Neurol. 2019;527:187–211. 27391320 10.1002/cne.24072 PMC5219957

[188] Charalambakis NE, Govindaiah G, Campbell PW, Guido W. Developmental remodeling of thalamic interneurons requires retinal signaling. J Neurosci. 2019;39:3856–66. 30842249 10.1523/JNEUROSCI.2224-18.2019 PMC6520504

[189] Toonen JA, Ma Y, Gutmann DH. Defining the temporal course of murine neurofibromatosis-1 optic gliomagenesis reveals a therapeutic window to attenuate retinal dysfunction. Neuro-Oncol. 2016;19:808–19. 10.1093/neuonc/now267 PMC5464459 28039362

[190] Diggs-Andrews KA, Brown JA, Gianino SM, Rubin JB, Wozniak DF, Gutmann DH. Sex Is a major determinant of neuronal dysfunction in neurofibromatosis type 1. Ann Neurol. 2014;75:309–16. 24375753 10.1002/ana.24093 PMC4172335

[191] de Blank PMK, Fisher MJ, Liu GT, Gutmann DH, Listernick R, Ferner RE, et al. Optic pathway gliomas in neurofibromatosis type 1. J Neuro-Ophthalmol. 2017;37:S23–32. 10.1097/WNO.0000000000000550 PMC7410089 28806346

[192] Brown JA, Gianino SM, Gutmann DH. Defective cAMP generation underlies the sensitivity of CNS neurons to neurofibromatosis-1 heterozygosity. J Neurosci. 2010;30:5579–89. 20410111 10.1523/JNEUROSCI.3994-09.2010 PMC2864934

[193] Toonen JA, Solga AC, Ma Y, Gutmann DH. Estrogen activation of microglia underlies the sexually dimorphic differences in Nf1 optic glioma–induced retinal pathology. J Exp Med. 2017;214:17–25. 27923908 10.1084/jem.20160447 PMC5206494

[194] Freret ME, Gutmann DH. Insights into optic pathway glioma vision loss from mouse models of neurofibromatosis type 1. J Neurosci Res. 2019;97:45–56. 29704429 10.1002/jnr.24250 PMC6766750

[195] Richardson R, Tracey-White D, Webster A, Moosajee M. The zebrafish eye—a paradigm for investigating human ocular genetics. Eye. 2017;31:68–86. 27612182 10.1038/eye.2016.198 PMC5233929

[196] Hong Y, Luo Y. Zebrafish model in ophthalmology to study disease mechanism and drug discovery. Pharmaceuticals. 2021;14:716. 34451814 10.3390/ph14080716 PMC8400593

[197] Link BA, Gray MP, Smith RS, John SWM. Intraocular pressure in zebrafish: comparison of inbred strains and identification of a reduced melanin mutant with raised IOP. Investig Opthalmology Vis Sci. 2004;45:4415. 10.1167/iovs.04-0557 15557450

[198] Veth KN, Willer JR, Collery RF, Gray MP, Willer GB, Wagner DS, et al. Mutations in zebrafish lrp2 result in adult-onset ocular pathogenesis that models myopia and other risk factors for glaucoma. PLoS Genet. 2011;7:e1001310. 21379331 10.1371/journal.pgen.1001310 PMC3040661

[199] Bouhenni RA, Dunmire J, Sewell A, Edward DP. Animal models of glaucoma. J Biomed Biotechnol. 2012;2012:692609. 22665989 10.1155/2012/692609 PMC3364028

[200] Volland S, Esteve-Rudd J, Hoo J, Yee C, Williams DS. A Comparison of some organizational characteristics of the mouse central retina and the human macula. PLoS One. 2015;10:e0125631. 25923208 10.1371/journal.pone.0125631 PMC4414478

[201] Quigley HA, Green WR. The histology of human glaucoma cupping and optic nerve damage: clinicopathologic correlation in 21 eyes. Ophthalmology. 1979;86:1803–27. 553256 10.1016/s0161-6420(79)35338-6

[202] Sajdak BS, Salmon AE, Cava JA, Allen KP, Freling S, Ramamirtham R, et al. Noninvasive imaging of the tree shrew eye: wavefront analysis and retinal imaging with correlative histology. Exp Eye Res. 2019;185:107683. 31158381 10.1016/j.exer.2019.05.023 PMC6698412

[203] Samuels BC, Siegwart JT, Zhan W, Hethcox L, Chimento M, Whitley R, et al. A Novel Tree Shrew (Tupaia belangeri) Model of Glaucoma. Invest Ophth Vis Sci. 2018;59:3136–43. 10.1167/iovs.18-24261 PMC6018453 30025140

[204] McLellan GJ, Miller PE. Feline glaucoma—a comprehensive review. Vet Ophthalmol. 2011;14:15–29. 21923820 10.1111/j.1463-5224.2011.00912.x PMC3348181

[205] Park SA, Sledge D, Monahan C, Bartoe JT, Komáromy AM. Primary angle-closure glaucoma with goniodysgenesis in a Beagle dog. Bmc Vet Res. 2019;15:75. 30832652 10.1186/s12917-019-1812-1 PMC6399873

[206] Kuehn MH, Lipsett KA, Menotti-Raymond M, Whitmore SS, Scheetz TE, David VA, et al. A mutation in LTBP2 causes congenital glaucoma in domestic cats (Felis catus). PLoS One. 2016;11:e0154412. 27149523 10.1371/journal.pone.0154412 PMC4858209

[207] Adelman SA, Oikawa K, Senthilkumar G, Trane RM, Teixeira LBC, McLellan GJ. Mapping retinal ganglion cell somas in a large-eyed glaucoma model. Mol Vis. 2020;27:608–21. PMC8645189 34924741

[208] Oikawa K, Teixeira LBC, Keikhosravi A, Eliceiri KW, McLellan GJ. Microstructure and resident cell-types of the feline optic nerve head resemble that of humans. Exp Eye Res. 2021;202:108315. 33091431 10.1016/j.exer.2020.108315 PMC7855208

[209] Middleton S. Porcine ophthalmology. Vet Clin North Am Food Animal Pract. 2010;26:557–72. 10.1016/j.cvfa.2010.09.002 21056801

[210] Garcá M, Ruiz-Ederra J, Hernández-Barbáchano H, Vecino E. Topography of pig retinal ganglion cells. J Comp Neurol. 2005;486:361–72. 15846788 10.1002/cne.20516

[211] Ruiz-Ederra J, García M, Hicks D, Vecino E. Comparative study of the three neurofilament subunits within pig and human retinal ganglion cells. Mol Vis. 2004;10:83–92. 14961007

[212] Veiga-Crespo P, del Río P, Blindert M, Ueffing M, Hauck SM, Vecino E. Phenotypic map of porcine retinal ganglion cells. Mol Vis. 2012;19:904–16. PMC3654859 23687427

[213] Galdos M, Bayón A, Rodriguez FD, Micó C, Sharma SC, Vecino E. Morphology of retinal vessels in the optic disk in a Göttingen minipig experimental glaucoma model. Vet Ophthalmol. 2012;15:36–46. 22050782 10.1111/j.1463-5224.2011.00937.x

[214] Suarez T, Vecino E. Expression of endothelial leukocyte adhesion molecule 1 in the aqueous outflow pathway of porcine eyes with induced glaucoma. Mol Vis. 2006;12:1467–72. 17167401

[215] SC VES. Elevated Intraocular Pressure induces Ultrastructural Changes in the Trabecular Meshwork. J Cytol Histol. 2015;S3:007.

[216] Cassina M, Frizziero L, Opocher E, Parrozzani R, Sorrentino U, Viscardi E, et al. Optic pathway glioma in type 1 neurofibromatosis: review of its pathogenesis, diagnostic assessment, and treatment recommendations. Cancers. 2019;11:1790. 31739524 10.3390/cancers11111790 PMC6896195

[217] Solga AC, Toonen JA, Pan Y, Cimino PJ, Ma Y, Castillon GA, et al. The cell of origin dictates the temporal course of neurofibromatosis-1 (Nf1) low-grade glioma formation. Oncotarget. 2017;8:47206–15. 28525381 10.18632/oncotarget.17589 PMC5564557

[218] Toonen JA, Anastasaki C, Smithson LJ, Gianino SM, Li K, Kesterson RA, et al. NF1 germline mutation differentially dictates optic glioma formation and growth in neurofibromatosis-1. Hum Mol Genet. 2016;25:1703–13. 26908603 10.1093/hmg/ddw039 PMC4986327

[219] Kaul A, Toonen JA, Gianino SM, Gutmann DH. The impact of coexisting genetic mutations on murine optic glioma biology. Neuro-Oncol. 2015;17:670–7. 25246427 10.1093/neuonc/nou287 PMC4482850

[220] Isakson SH, Rizzardi AE, Coutts AW, Carlson DF, Kirstein MN, Fisher J, et al. Genetically engineered minipigs model the major clinical features of human neurofibromatosis type 1. Commun Biol. 2018;1:158. 30302402 10.1038/s42003-018-0163-y PMC6168575

[221] Mitra S, Devi S, Lee M-S, Jui J, Sahu A, Goldman D. Vegf signaling between Müller glia and vascular endothelial cells is regulated by immune cells and stimulates retina regeneration. Proc Natl Acad Sci. 2022;119:e2211690119. 36469778 10.1073/pnas.2211690119 PMC9897474

[222] Zhang KY, Aguzzi EA, Johnson TV. Retinal ganglion cell transplantation: approaches for overcoming challenges to functional integration. Cells. 2021;10:1426. 34200991 10.3390/cells10061426 PMC8228580

[223] Wu S, Chang K-C, Nahmou M, Goldberg JL. Induced pluripotent stem cells promote retinal ganglion cell survival after transplant. Invest Ophth Vis Sci. 2018;59:1571–6. 10.1167/iovs.17-23648 PMC5863687 29625481

[224] Abud MB, Baranov P, Patel S, Hicks CA, Isaac DLC, Louzada RN, et al. In vivo study to assess dosage of allogeneic pig retinal progenitor cells: Long-term survival, engraftment, differentiation and safety. J Cell Mol Medicine. 2022;26:3254–68. 10.1111/jcmm.17332 PMC9170813 35481949

[225] Mead B, Scheven BA. Mesenchymal stem cell therapy for retinal ganglion cell neuroprotection and axon regeneration. Neural Regen Res. 2015;10:371–3. 25878580 10.4103/1673-5374.153681 PMC4396094

[226] Chichagova V, Georgiou M, Carter M, Dorgau B, Hilgen G, Collin J, et al. Incorporating microglia-like cells in human induced pluripotent stem cell-derived retinal organoids. J Cell Mol Med. 2023;27:435–45. 36644817 10.1111/jcmm.17670 PMC9889627

[227] VanderWall KB, Vij R, Ohlemacher SK, Sridhar A, Fligor CM, Feder EM, et al. Astrocytes regulate the development and maturation of retinal ganglion cells derived from human pluripotent stem cells. Stem Cell Rep. 2019;12:201–12. 10.1016/j.stemcr.2018.12.010 PMC6373493 30639213

[228] Xu H, Chen M, Forrester JV. Para-inflammation in the aging retina. Prog Retin Eye Res. 2009;28:348–68. 19560552 10.1016/j.preteyeres.2009.06.001

[229] Baudouin C, Kolko M, Melik-Parsadaniantz S, Messmer EM. Inflammation in Glaucoma: from the back to the front of the eye, and beyond. Prog Retin Eye Res. 2021;83:100916. 33075485 10.1016/j.preteyeres.2020.100916

[230] Andries L, Kancheva D, Masin L, Scheyltjens I, Hove HV, Vlaminck KD, et al. Immune stimulation recruits a subset of pro-regenerative macrophages to the retina that promotes axonal regrowth of injured neurons. Acta Neuropathol Commun. 2023;11:85. 37226256 10.1186/s40478-023-01580-3 PMC10210300

[231] Pierdomenico JD, Henderson DCM, Giammaria S, Smith VL, Jamet AJ, Smith CA, et al. Age and intraocular pressure in murine experimental glaucoma. Prog Retin Eye Res. 2022;88:101021. 34801667 10.1016/j.preteyeres.2021.101021

[232] Hoffelen SJV, Young MJ, Shatos MA, Sakaguchi DS. Incorporation of murine brain progenitor cells into the developing mammalian retina. Investig Opthalmol Vis Sci. 2003;44:426. 10.1167/iovs.02-0269 12506105

[233] Sakaguchi DS, van Hoffelen SJ, Theusch E, Parker E, Orasky J, Harper MM, et al. Transplantation of neural progenitor cells into the developing retina of the Brazilian opossum: an in vivo system for studying stem/progenitor cell plasticity. Dev Neurosci-basel. 2005;26:336–45. 10.1159/000082275 15855762

[234] Yao J, Feathers KL, Khanna H, Thompson D, Tsilfidis C, Hauswirth WW, et al. XIAP therapy increases survival of transplanted rod precursors in a degenerating host retina. Investig Opthalmol Vis Sci. 2011;52:1567. 10.1167/iovs.10-5998 PMC3101692 20926819

[235] Eberle D, Santos-Ferreira T, Grahl S, Ader M. Subretinal Transplantation of MACS Purified Photoreceptor Precursor Cells into the Adult Mouse Retina. J Vis Exp. 2014;e50932. 10.3791/50932 PMC4130385 24638161

[236] Soucy JR, Todd L, Kriukov E, Phay M, Reh TA, Baranov P. Introduced chemokine gradients guide transplanted and regenerated retinal neurons toward their natural position in the retina. Biorxiv. 2022;2022.09.29.510158.

[237] Xu Q, Boylan NJ, Suk JS, Wang Y-Y, Nance EA, Yang J-C, et al. Nanoparticle diffusion in, and microrheology of, the bovine vitreous ex vivo. J Control Release. 2013;167:76–84. 23369761 10.1016/j.jconrel.2013.01.018 PMC3693951

[238] Xian B, Huang B. The immune response of stem cells in subretinal transplantation. Stem Cell Res Ther. 2015;6:161. 26364954 10.1186/s13287-015-0167-1 PMC4568575

[239] Forrester JV, Xu H. Good news–bad news: the Yin and Yang of immune privilege in the eye. Front Immunol. 2012;3:338. 23230433 10.3389/fimmu.2012.00338 PMC3515883

[240] Peynshaert K, Devoldere J, Minnaert AK, Smedt SCD, Remaut K. Morphology and Composition of the Inner Limiting Membrane: Species-Specific Variations and Relevance toward Drug Delivery Research. Curr Eye Res. 2019;44:465–75. 30638413 10.1080/02713683.2019.1565890

[241] Johnson TV, Bull ND, Martin KR. Identification of barriers to retinal engraftment of transplanted stem cells. Investig Opthalmol Vis Sci. 2010;51:960. 10.1167/iovs.09-3884 PMC2868445 19850833

[242] Zhang KY, Tuffy C, Mertz JL, Quillen S, Wechsler L, Quigley HA, et al. Role of the internal limiting membrane in structural engraftment and topographic spacing of transplanted human stem cell-derived retinal ganglion cells. Stem Cell Rep. 2021;16:149–67. 10.1016/j.stemcr.2020.12.001 PMC7897583 33382979

[243] Aguzzi EA, Zhang KY, Nagalingam A, Quillen S, Hariharakumar S, Chetla N, et al. Internal limiting membrane disruption facilitates engraftment of transplanted human stem cell derived retinal ganglion cells. Biorxiv. 2022;2022.12.13.519327.

[244] Teo KYC, Lee SY, Barathi AV, Tun SBB, Tan L, Constable IJ. Surgical removal of internal limiting membrane and layering of AAV vector on the retina under air enhances gene transfection in a nonhuman primate. Investig Opthalmol Vis Sci. 2018;59:3574. 10.1167/iovs.18-24333 30025098

[245] Peynshaert K, Vanluchene H, Clerck KD, Minnaert A-K, Verhoeven M, Gouspillou N, et al. ICG-mediated photodisruption of the inner limiting membrane enhances retinal drug delivery. J Control Release. 2022;349:315–26. 35803327 10.1016/j.jconrel.2022.07.002

[246] Becker S, Eastlake K, Jayaram H, Jones MF, Brown RA, McLellan GJ, et al. Allogeneic transplantation of müller-derived retinal ganglion cells improves retinal function in a feline model of ganglion cell depletion. Stem Cell Transl Med. 2016;5:192–205. 10.5966/sctm.2015-0125 PMC4729554 26718648

[247] Luo Z, Xian B, Li K, Li K, Yang R, Chen M, et al. Biodegradable scaffolds facilitate epiretinal transplantation of hiPSC-Derived retinal neurons in nonhuman primates. Acta Biomater. 2021;134:289–301. 34314890 10.1016/j.actbio.2021.07.040

[248] Dromel PC, Singh D, Andres E, Likes M, Kurisawa M, Alexander-Katz A, et al. A bioinspired gelatin-hyaluronic acid-based hybrid interpenetrating network for the enhancement of retinal ganglion cells replacement therapy. Npj Regen Medicine. 2021;6:85. 10.1038/s41536-021-00195-3 PMC8688498 34930951

[249] Dromel PC, Singh D, Alexander-Katz A, Kurisawa M, Spector M, Young M. Mechano-chemical effect of gelatin- and HA-based hydrogels on human retinal progenitor cells. Prog Coll Pol Sci S. 2023;9:58. 10.3390/gels9010058 PMC9858647 36661824

[250] Kador KE, Montero RB, Venugopalan P, Hertz J, Zindell AN, Valenzuela DA, et al. Tissue engineering the retinal ganglion cell nerve fiber layer. Biomaterials. 2013;34:4242–50. 23489919 10.1016/j.biomaterials.2013.02.027 PMC3608715

[251] Gamlin PD, Alexander JJ, Boye SL, Witherspoon CD, Boye SE. Adeno-associated virus vectors, design and delivery. Methods Mol Biol. 2019;1950:249–62. 30783978 10.1007/978-1-4939-9139-6_14 PMC6700748

[252] Malagobadan S, Nagoor NH. Reference Module in Biomedical Sciences. 2017.

[253] Golan N, Cafferty WB. Dissociation of intact adult mouse cortical projection neurons for single-cell RNA-seq. STAR Protoc. 2021;2:100941. 34877546 10.1016/j.xpro.2021.100941 PMC8633369

[254] Lin KT, Wang A, Nguyen AB, Iyer J, Tran SD. Recent advances in hydrogels: ophthalmic applications in cell delivery, vitreous substitutes, and ocular adhesives. Biomed. 2021;9:1203. 10.3390/biomedicines9091203 PMC8471559 34572389

[255] Wang J, Chu R, Ni N, Nan G. The effect of Matrigel as scaffold material for neural stem cell transplantation for treating spinal cord injury. Sci Rep. 2020;10:2576. 32054865 10.1038/s41598-020-59148-3 PMC7018993

[256] Carlson AL, Bennett NK, Francis NL, Halikere A, Clarke S, Moore JC, et al. Generation and transplantation of reprogrammed human neurons in the brain using 3D microtopographic scaffolds. Nat Commun. 2016;7:10862. 26983594 10.1038/ncomms10862 PMC4800432

[257] Moeinabadi-Bidgoli K, Babajani A, Yazdanpanah G, Farhadihosseinabadi B, Jamshidi E, Bahrami S, et al. Translational insights into stem cell preconditioning: from molecular mechanisms to preclinical applications. Biomed Pharmacother. 2021;142:112026. 34411911 10.1016/j.biopha.2021.112026

[258] Hasel P, Aisenberg WH, Bennett FC, Liddelow SA. Molecular and metabolic heterogeneity of astrocytes and microglia. Cell Metab. 2023;35:555–70. 36958329 10.1016/j.cmet.2023.03.006

[259] Hasel P, Rose IVL, Sadick JS, Kim RD, Liddelow SA. Neuroinflammatory astrocyte subtypes in the mouse brain. Nat Neurosci. 2021;24:1475–87. 34413515 10.1038/s41593-021-00905-6

[260] Hammond TR, Dufort C, Dissing-Olesen L, Giera S, Young A, Wysoker A, et al. Single-cell RNA sequencing of microglia throughout the mouse lifespan and in the injured brain reveals complex cell-state changes. Immunity. 2019;50:253–271.e6. 30471926 10.1016/j.immuni.2018.11.004 PMC6655561

[261] Paolicelli RC, Sierra A, Stevens B, Tremblay M-E, Aguzzi A, Ajami B, et al. Microglia states and nomenclature: a field at its crossroads. Neuron. 2022;110:3458–83. 36327895 10.1016/j.neuron.2022.10.020 PMC9999291

[262] Ceyzériat K, Abjean L, Sauvage MAC, Haim LB, Escartin C. The complex STATes of astrocyte reactivity: How are they controlled by the JAK–STAT3 pathway? Neurosci. 2016;330:205–18. 10.1016/j.neuroscience.2016.05.043 27241943

[263] Yan Z, Gibson SA, Buckley JA, Qin H, Benveniste EN. Role of the JAK/STAT signaling pathway in regulation of innate immunity in neuroinflammatory diseases. Clin Immunol. 2018;189:4–13. 27713030 10.1016/j.clim.2016.09.014 PMC5573639

[264] Liddelow SA, Guttenplan KA, Clarke LE, Bennett FC, Bohlen CJ, Schirmer L, et al. Neurotoxic reactive astrocytes are induced by activated microglia. Nature. 2017;541:481–7. 28099414 10.1038/nature21029 PMC5404890

[265] Liu L, Liu J, Bao J, Bai Q, Wang G. Interaction of microglia and astrocytes in the neurovascular unit. Front Immunol. 2020;11:1024. 32733433 10.3389/fimmu.2020.01024 PMC7362712

[266] Guttenplan KA, Stafford BK, El-Danaf RN, Adler DI, Münch AE, Weigel MK, et al. Neurotoxic reactive astrocytes drive neuronal death after retinal injury. Cell Rep. 2020;31:107776. 32579912 10.1016/j.celrep.2020.107776 PMC8091906

[267] Sterling JK, Adetunji MO, Guttha S, Bargoud AR, Uyhazi KE, Ross AG, et al. GLP-1 receptor agonist NLY01 reduces retinal inflammation and neuron death secondary to ocular hypertension. Cell Rep. 2020;33:108271. 33147455 10.1016/j.celrep.2020.108271 PMC7660987

[268] Lawrence ECN, Guo M, Schwartz TD, Wu J, Lu J, Nikonov S, et al. Topical and systemic GLP-1R agonist administration both rescue retinal ganglion cells in hypertensive glaucoma. Front Cell Neurosci. 2023;17:1156829. 37362000 10.3389/fncel.2023.1156829 PMC10288152

[269] Guttenplan KA, Weigel MK, Prakash P, Wijewardhane PR, Hasel P, Rufen-Blanchette U, et al. Neurotoxic reactive astrocytes induce cell death via saturated lipids. Nature. 2021;599:102–7. 34616039 10.1038/s41586-021-03960-y PMC12054010

[270] Anderson MA, Burda JE, Ren Y, Ao Y, O’Shea TM, Kawaguchi R, et al. Astrocyte scar formation aids central nervous system axon regeneration. Nature. 2016;532:195–200. 27027288 10.1038/nature17623 PMC5243141

[271] Gomes C, VanderWall KB, Pan Y, Lu X, Lavekar SS, Huang K-C, et al. Astrocytes modulate neurodegenerative phenotypes associated with glaucoma in OPTN(E50K) human stem cell-derived retinal ganglion cells. Stem Cell Rep. 2022;17:1636–49. 10.1016/j.stemcr.2022.05.006 PMC9287669 35714595

[272] Zhao X, Sun R, Luo X, Wang F, Sun X. The interaction between microglia and macroglia in glaucoma. Front Neurosci-switz. 2021;15:610788. 10.3389/fnins.2021.610788 PMC8193936 34121982

[273] Ransom BR, Orkand RK. Glial-neuronal interactions in non-synaptic areas of the brain: studies in the optic nerve. Trends Neurosci. 1996;19:352–8. 8843605 10.1016/0166-2236(96)10045-x

[274] Ridet JL, Privat A, Malhotra SK, Gage FH. Reactive astrocytes: cellular and molecular cues to biological function. Trends Neurosci. 1997;20:570–7. 9416670 10.1016/s0166-2236(97)01139-9

[275] Linnerbauer M, Rothhammer V. Protective functions of reactive astrocytes following central nervous system insult. Front Immunol. 2020;11:573256. 33117368 10.3389/fimmu.2020.573256 PMC7561408

[276] Tezel G. Molecular regulation of neuroinflammation in glaucoma: Current knowledge and the ongoing search for new treatment targets. Prog Retin Eye Res. 2022;87:100998. 34348167 10.1016/j.preteyeres.2021.100998 PMC8803988

[277] Cooper ML, Crish SD, Inman DM, Horner PJ, Calkins DJ. Early astrocyte redistribution in the optic nerve precedes axonopathy in the DBA/2J mouse model of glaucoma. Exp Eye Res. 2016;150:22–33. 26646560 10.1016/j.exer.2015.11.016 PMC4889569

[278] Cooper ML, Collyer JW, Calkins DJ. Astrocyte remodeling without gliosis precedes optic nerve axonopathy. Acta Neuropathologica Commun. 2018;6:38. 10.1186/s40478-018-0542-0 PMC5946396 29747701

[279] Paschalis EI, Lei F, Zhou C, Kapoulea V, Thanos A, Dana R, et al. The role of microglia and peripheral monocytes in retinal damage after corneal chemical injury. Am J Pathology. 2018;188:1580–96. 10.1016/j.ajpath.2018.03.005 PMC6136091 29630857

[280] García-Bermúdez MY, Freude KK, Mouhammad ZA, van Wijngaarden P, Martin KK, Kolko M. Glial cells in glaucoma: friends, foes, and potential therapeutic targets. Front Neurol. 2021;12:624983. 33796062 10.3389/fneur.2021.624983 PMC8007906

[281] Bessoles S, Grandclément C, Alari-Pahissa E, Gehrig J, Jeevan-Raj B, Held W. Adaptations of Natural Killer Cells to Self-MHC Class I. Front Immunol. 2014;5:349. 25101089 10.3389/fimmu.2014.00349 PMC4106420

[282] Taylor AL, Negus SL, Negus M, Bolton EM, Bradley JA, Pettigrew GJ. Pathways of helper CD4 T cell allorecognition in generating alloantibody and CD8 T cell alloimmunity. Transplantation. 2007;83:931–7. 17460565 10.1097/01.tp.0000257960.07783.e3

[283] Diehl R, Ferrara F, Müller C, Dreyer AY, McLeod DD, Fricke S, et al. Immunosuppression for in vivo research: state-of-the-art protocols and experimental approaches. Cell Mol Immunol. 2017;14:146–79. 27721455 10.1038/cmi.2016.39 PMC5301156

[284] Lilienfeld BG, Crew MD, Forte P, Baumann BC, Seebach JD. Transgenic expression of HLA-E single chain trimer protects porcine endothelial cells against human natural killer cell-mediated cytotoxicity. Xenotransplantation. 2007;14:126–34. 17381687 10.1111/j.1399-3089.2007.00378.x

[285] Morizane A, Doi D, Kikuchi T, Okita K, Hotta A, Kawasaki T, et al. Direct comparison of autologous and allogeneic transplantation of iPSC-derived neural cells in the brain of a nonhuman primate. Stem Cell Rep. 2013;1:283–92. 10.1016/j.stemcr.2013.08.007 PMC3849265 24319664

[286] de Rham C, Villard J. Potential and limitation of HLA-based banking of human pluripotent stem cells for cell therapy. J Immunol Res. 2014;2014:518135. 25126584 10.1155/2014/518135 PMC4121106

[287] Madrid M, Sumen C, Aivio S, Saklayen N. autologous induced pluripotent stem cell-based cell therapies: promise, progress, and challenges. Curr Protoc. 2021;1:e88. 33725407 10.1002/cpz1.88

[288] Yin Y, Cui Q, Gilbert H, Yang Y, Yang Z, Berlinicke C, et al. Oncomodulin links inflammation to optic nerve regeneration. Proc Natl Acad Sci. 2009;106:19587–92. 19875691 10.1073/pnas.0907085106 PMC2780793

[289] Chang Y-C, Walston ST, Chow RH, Weiland JD. GCaMP expression in retinal ganglion cells characterized using a low-cost fundus imaging system. J Neural Eng. 2017;14:056018. 28930702 10.1088/1741-2552/aa7ded PMC6651747

[290] Smith CA, Chauhan BC. In vivo imaging of adeno-associated viral vector labelled retinal ganglion cells. Sci Rep-uk. 2018;8:1490. 10.1038/s41598-018-19969-9 PMC5784170 29367685

[291] Hong G, Fu TM, Qiao M, Viveros RD, Yang X, Zhou T, et al. A method for single-neuron chronic recording from the retina in awake mice. Science. 2018;360:1447–51. 29954976 10.1126/science.aas9160 PMC6047945

[292] Smith CA, Vianna JR, Chauhan BC. Assessing retinal ganglion cell damage Eye. 2017;31:209–17. 28085141 10.1038/eye.2016.295 PMC5306472

[293] Smith BJ, Wang X, Chauhan BC, Côté PD, Tremblay F. Contribution of retinal ganglion cells to the mouse electroretinogram. Doc Ophthalmol. 2014;128:155–68. 24659322 10.1007/s10633-014-9433-2

[294] Liu Z, Kurokawa K, Zhang F, Lee JJ, Miller DT. Imaging and quantifying ganglion cells and other transparent neurons in the living human retina. Proc National Acad Sci. 2017;114:12803–8. 10.1073/pnas.1711734114 PMC5715765 29138314

[295] Rossi EA, Granger CE, Sharma R, Yang Q, Saito K, Schwarz C, et al. Imaging individual neurons in the retinal ganglion cell layer of the living eye. Proc National Acad Sci. 2017;114:586–91. 10.1073/pnas.1613445114 PMC5255596 28049835

[296] Miller DT, Kurokawa K. Cellular scale imaging of transparent retinal structures and processes using adaptive optics optical coherence tomography. Annu Rev Vis Sc. 2020;6:1–34. 32609578 10.1146/annurev-vision-030320-041255 PMC7864592

[297] Takagi S, Mandai M, Gocho K, Hirami Y, Yamamoto M, Fujihara M, et al. Evaluation of transplanted autologous induced pluripotent stem cell-derived retinal pigment epithelium in exudative age-related macular degeneration. Ophthalmol Retin. 2019;3:850–9. 10.1016/j.oret.2019.04.021 31248784

[298] Aboualizadeh E, Phillips MJ, McGregor JE, DiLoreto DA, Strazzeri JM, Dhakal KR, et al. Imaging transplanted photoreceptors in living nonhuman primates with single-cell resolution. Stem Cell Rep. 2020;15:482–97. 10.1016/j.stemcr.2020.06.019 PMC7419740 32707075

[299] Walters S, Feeks JA, Huynh KT, Hunter JJ. Adaptive optics two-photon excited fluorescence lifetime imaging ophthalmoscopy of photoreceptors and retinal pigment epithelium in the living non-human primate eye. Biomed Opt Express. 2021;13:389. 35154879 10.1364/BOE.444550 PMC8803039

[300] Geng Y, Schery LA, Sharma R, Dubra A, Ahmad K, Libby RT, et al. Optical properties of the mouse eye. Biomed Opt Express. 2011;2:717–38. 21483598 10.1364/BOE.2.000717 PMC3072116

[301] Lu GJ, Chou L, Malounda D, Patel AK, Welsbie DS, Chao DL, et al. Genetically encodable contrast agents for optical coherence tomography. ACS Nano. 2020;14:7823–31. 32023037 10.1021/acsnano.9b08432 PMC7685218

[302] Yang C. Molecular contrast optical coherence tomography: a review. Photochem Photobiol. 2004;81:215–37. 10.1562/2004-08-06-IR-266 PMC1283114 15588122

[303] Ehlers JP, Gupta PK, Farsiu S, Maldonado R, Kim T, Toth CA, et al. Evaluation of contrast agents for enhanced visualization in optical coherence tomography. Investig Opthalmol Vis Sci. 2010;51:6614. 10.1167/iovs.10-6195 21051711

[304] Yuan S, Roney CA, Wierwille J, Chen C-W, Xu B, Griffiths G, et al. Co-registered optical coherence tomography and fluorescence molecular imaging for simultaneous morphological and molecular imaging. Phys Med Biol. 2009;55:191–206. 10.1088/0031-9155/55/1/011 PMC2951762 20009192

[305] Li X, Zhang W, Li Y, Wu X, Wang M, Tan X, et al. In vivo cell tracking using multimodality imaging. Biophotonics Congress: Biomedical Optics 2022. Paper OS4D.5. 10.1364/OTS.2022.OS4D.5.

[306] Liba O, SoRelle ED, Sen D, de la Zerda A. Contrast-enhanced optical coherence tomography with picomolar sensitivity for functional in vivo imaging. Sci Rep-uk. 2016;6:23337. 10.1038/srep23337 PMC4796912 26987475

[307] Vrathasha V, Nikonov S, Bell BA, He J, Bungatavula Y, Uyhazi KE, et al. Transplanted human induced pluripotent stem cells- derived retinal ganglion cells embed within mouse retinas and are electrophysiologically functional. Iscience. 2022;25:105308. 36388952 10.1016/j.isci.2022.105308 PMC9646916

[308] Singh MS, Balmer J, Barnard AR, Aslam SA, Moralli D, Green CM, et al. Transplanted photoreceptor precursors transfer proteins to host photoreceptors by a mechanism of cytoplasmic fusion. Nat Commun. 2016;7:13537. 27901042 10.1038/ncomms13537 PMC5141374

[309] Kim I-J, Zhang Y, Yamagata M, Meister M, Sanes JR. Molecular identification of a retinal cell type that responds to upward motion. Nature. 2008;452:478–82. 18368118 10.1038/nature06739

[310] Field GD, Gauthier JL, Sher A, Greschner M, Machado T, Jepson LH, et al. Functional connectivity in the retina at the resolution of photoreceptors. Nature. 2010;467:673–7. 20930838 10.1038/nature09424 PMC2953734

[311] Baden T, Berens P, Franke K, Rosón MR, Bethge M, Euler T. The functional diversity of retinal ganglion cells in the mouse. Nature. 2016;529:345–50. 26735013 10.1038/nature16468 PMC4724341

[312] Porciatti V. Electrophysiological assessment of retinal ganglion cell function. Exp Eye Res. 2015;141:164–70. 25998495 10.1016/j.exer.2015.05.008 PMC4628896

[313] He XY, Zhao CJ, Xu H, Chen K, Bian BSJ, Gong Y, et al. Synaptic repair and vision restoration in advanced degenerating eyes by transplantation of retinal progenitor cells. Stem Cell Rep. 2021;16:1805–17. 10.1016/j.stemcr.2021.06.002 PMC8282465 34214489

[314] Pfäffle C, Spahr H, Kutzner L, Burhan S, Hilge F, Miura Y, et al. Simultaneous functional imaging of neuronal and photoreceptor layers in living human retina. Opt Lett. 2019;44:5671. 31774751 10.1364/OL.44.005671

[315] Hilgen G, Kartsaki E, Kartysh V, Cessac B, Sernagor E. A novel approach to the functional classification of retinal ganglion cells. Open Biol. 2022;12:210367. 35259949 10.1098/rsob.210367 PMC8905177

[316] Milosavljevic N, Storchi R, Eleftheriou CG, Colins A, Petersen RS, Lucas RJ. Photoreceptive retinal ganglion cells control the information rate of the optic nerve. Proc National Acad Sci. 2018;115:201810701. 10.1073/pnas.1810701115 PMC6294960 30487225

[317] Li Y, Xia X, Paulus YM. Advances in retinal optical imaging. Photonics. 2018;5:9. 31321222 10.3390/photonics5020009 PMC6639094

[318] Tribble JR, Hui F, Quintero H, Hajji SE, Bell K, Polo AD, et al. Neuroprotection in glaucoma: mechanisms beyond intraocular pressure lowering. Mol Asp Med. 2023;92:101193. 10.1016/j.mam.2023.101193 37331129

[319] Chao JR, Lamba DA, Klesert TR, Torre AL, Hoshino A, Taylor RJ, et al. Transplantation of human embryonic stem cell-derived retinal cells into the subretinal space of a non-human primate. Transl Vis Sci Technol. 2017;6:4. 28516002 10.1167/tvst.6.3.4 PMC5433804

[320] Yu-Wai-Man P, Griffiths PG, Chinnery PF. Mitochondrial optic neuropathies – disease mechanisms and therapeutic strategies. Prog Retin Eye Res. 2011;30:81–114. 21112411 10.1016/j.preteyeres.2010.11.002 PMC3081075

[321] Rajala RVS. Aerobic glycolysis in the retina: functional roles of pyruvate kinase isoforms. Front Cell Dev Biol. 2020;8:266. 32426353 10.3389/fcell.2020.00266 PMC7203425

[322] Williams PA, Harder JM, Foxworth NE, Cochran KE, Philip VM, Porciatti V, et al. Vitamin B3 modulates mitochondrial vulnerability and prevents glaucoma in aged mice. Science. 2017;355:756–60. 28209901 10.1126/science.aal0092 PMC5408298

[323] Tribble JR, Otmani A, Sun S, Ellis SA, Cimaglia G, Vohra R, et al. Nicotinamide provides neuroprotection in glaucoma by protecting against mitochondrial and metabolic dysfunction. Redox Biol. 2021;43:101988. 33932867 10.1016/j.redox.2021.101988 PMC8103000

[324] Hui F, Tang J, Williams PA, McGuinness MB, Hadoux X, Casson RJ, et al. Improvement in inner retinal function in glaucoma with nicotinamide (vitamin B3) supplementation: a crossover randomized clinical trial. Clin Exp Ophthalmol. 2020;48:903–14. 32721104 10.1111/ceo.13818

[325] Yu DY, Cringle SJ. Oxygen distribution and consumption within the retina in vascularised and avascular retinas and in animal models of retinal disease. Prog Retin Eye Res. 2001;20:175–208. 11173251 10.1016/s1350-9462(00)00027-6

[326] Harder JM, Guymer C, Wood JPM, Daskalaki E, Chidlow G, Zhang C, et al. Disturbed glucose and pyruvate metabolism in glaucoma with neuroprotection by pyruvate or rapamycin. Proc National Acad Sci. 2020;117:33619–27. 10.1073/pnas.2014213117 PMC7776900 33318177

[327] Moraes CGD, John SWM, Williams PA, Blumberg DM, Cioffi GA, Liebmann JM. Nicotinamide and pyruvate for neuroenhancement in open-angle glaucoma. JAMA Ophthalmol. 2022;140:11–8. 34792559 10.1001/jamaophthalmol.2021.4576 PMC8603231

[328] Philips T, Rothstein JD. Oligodendroglia: metabolic supporters of neurons. J Clin Investig. 2017;127:3271–80. 28862639 10.1172/JCI90610 PMC5669561

[329] Benedetto MM, Contin MA. Oxidative Stress in Retinal Degeneration Promoted by Constant LED Light. Front Cell Neurosci. 2019;13:139. 31105526 10.3389/fncel.2019.00139 PMC6499158

[330] Boia R, Ruzafa N, Aires ID, Pereiro X, Ambrósio AF, Vecino E, et al. Neuroprotective strategies for retinal ganglion cell degeneration: current status and challenges ahead. Int J Mol Sci. 2020;21:2262. 32218163 10.3390/ijms21072262 PMC7177277

[331] Sánchez-Migallón MC, Valiente-Soriano FJ, Nadal-Nicolás FM, Vidal-Sanz M, Agudo-Barriuso M. Apoptotic retinal ganglion cell death after optic nerve transection or crush in mice: delayed RGC loss with BDNF or a caspase 3 inhibitor. Investig Opthalmol Vis Sci. 2016;57:81. 10.1167/iovs.15-17841 26780312

[332] Ahmed Z, Kalinski H, Berry M, Almasieh M, Ashush H, Slager N, et al. Ocular neuroprotection by siRNA targeting caspase-2. Cell Death Dis. 2011;2:e173–e173. 21677688 10.1038/cddis.2011.54 PMC3168996

[333] Thomas CN, Bernardo-Colón A, Courtie E, Essex G, Rex TS, Blanch RJ, et al. Effects of intravitreal injection of siRNA against caspase-2 on retinal and optic nerve degeneration in air blast induced ocular trauma. Sci Rep-uk. 2021;11:16839. 10.1038/s41598-021-96107-y PMC8377143 34413361

[334] Fatokun AA, Dawson VL, Dawson TM. Players in parthanatos. Brit. J Pharmacol. 2014;171:2000–16. 10.1111/bph.12416 PMC3976618 24684389

[335] Chen L, Zhang X, Ou Y, Liu M, Yu D, Song Z, et al. Advances in RIPK1 kinase inhibitors. Front Pharmacol. 2022;13:976435. 36249746 10.3389/fphar.2022.976435 PMC9554302

[336] Welsbie DS, Yang Z, Ge Y, Mitchell KL, Zhou X, Martin SE, et al. Functional genomic screening identifies dual leucine zipper kinase as a key mediator of retinal ganglion cell death. Proc National Acad Sci. 2013;110:4045–50. 10.1073/pnas.1211284110 PMC3593842 23431148

[337] Lindsten T, Ross AJ, King A, Zong W-X, Rathmell JC, Shiels HA, et al. The combined functions of proapoptotic Bcl-2 family members bak and bax are essential for normal development of multiple tissues. Mol Cell. 2000;6:1389–99. 11163212 10.1016/s1097-2765(00)00136-2 PMC3057227

[338] Donahue RJ, Maes ME, Grosser JA, Nickells RW. BAX-depleted retinal ganglion cells survive and become quiescent following optic nerve damage. Mol Neurobiol. 2020;57:1070–84. 31673950 10.1007/s12035-019-01783-7 PMC7035206

[339] Welsbie DS, Ziogas NK, Xu L, Kim B-J, Ge Y, Patel AK, et al. Targeted disruption of dual leucine zipper kinase and leucine zipper kinase promotes neuronal survival in a model of diffuse traumatic brain injury. Mol Neurodegener. 2019;14:44. 31775817 10.1186/s13024-019-0345-1 PMC6882250

[340] Watkins TA, Wang B, Huntwork-Rodriguez S, Yang J, Jiang Z, Eastham-Anderson J, et al. DLK initiates a transcriptional program that couples apoptotic and regenerative responses to axonal injury. Proc National Acad Sci. 2013;110:4039–44. 10.1073/pnas.1211074110 PMC3593899 23431164

[341] Luo Z, Chang K-C, Wu S, Sun C, Xia X, Nahmou M, et al. Directly induced human retinal ganglion cells mimic fetal RGCs and are neuroprotective after transplantation in vivo. Stem Cell Rep. 2022;17:2690–703. 10.1016/j.stemcr.2022.10.011 PMC9768574 36368332

[342] Lucci C, Groef LD. On the other end of the line: extracellular vesicle-mediated communication in glaucoma. Front Neuroanat. 2023;17:1148956. 37113676 10.3389/fnana.2023.1148956 PMC10126352

[343] Bartsch U, Oriyakhel W, Kenna PF, Linke S, Richard G, Petrowitz B, et al. Retinal cells integrate into the outer nuclear layer and differentiate into mature photoreceptors after subretinal transplantation into adult mice. Exp Eye Res. 2008;86:691–700. 18329018 10.1016/j.exer.2008.01.018

[344] Daniszewski M, Senabouth A, Nguyen QH, Crombie DE, Lukowski SW, Kulkarni T, et al. Single cell RNA sequencing of stem cell-derived retinal ganglion cells. Sci Data. 2018;5:180013. 29437159 10.1038/sdata.2018.13 PMC5810423

[345] Liu YV, Santiago CP, Sogunro A, Konar GJ, Hu M, McNally MM, et al. Single-cell transcriptome analysis of xenotransplanted human retinal organoids defines two migratory cell populations of nonretinal origin. Stem Cell Rep. 2023;18:1138–54. 10.1016/j.stemcr.2023.04.004 PMC10202694 37163980

[346] Tyssowski KM, DeStefino NR, Cho JH, Dunn CJ, Poston RG, Carty CE, et al. Different neuronal activity patterns induce different gene expression programs. Neuron. 2018;98:530–546.e11. 29681534 10.1016/j.neuron.2018.04.001 PMC5934296

[347] Blankenship AG, Feller MB. Mechanisms underlying spontaneous patterned activity in developing neural circuits. Nat Rev Neurosci. 2010;11:18–29. 19953103 10.1038/nrn2759 PMC2902252

[348] Tehrani S, Davis L, Cepurna WO, Choe TE, Lozano DC, Monfared A, et al. Astrocyte structural and molecular response to elevated intraocular pressure occurs rapidly and precedes axonal tubulin rearrangement within the optic nerve head in a rat model. PLoS One. 2016;11:e0167364. 27893827 10.1371/journal.pone.0167364 PMC5125687

[349] Howell GR, Soto I, Zhu X, Ryan M, Macalinao DG, Sousa GL, et al. Radiation treatment inhibits monocyte entry into the optic nerve head and prevents neuronal damage in a mouse model of glaucoma. J Clin Invest. 2012;122:1246–61. 22426214 10.1172/JCI61135 PMC3314470

[350] Bosco A, Inman DM, Steele MR, Wu G, Soto I, Marsh-Armstrong N, et al. Reduced retina microglial activation and improved optic nerve integrity with minocycline treatment in the DBA/2J mouse model of glaucoma. Investig Opthalmol Vis Sci. 2008;49:1437. 10.1167/iovs.07-1337 18385061

[351] Chung W-S, Clarke LE, Wang GX, Stafford BK, Sher A, Chakraborty C, et al. Astrocytes mediate synapse elimination through MEGF10 and MERTK pathways. Nature. 2013;504:394–400. 24270812 10.1038/nature12776 PMC3969024

[352] Anderson SR, Zhang J, Steele MR, Romero CO, Kautzman AG, Schafer DP, et al. Complement targets newborn retinal ganglion cells for phagocytic elimination by microglia. J Neurosci. 2019;39:2025–40. 30647151 10.1523/JNEUROSCI.1854-18.2018 PMC6507095

[353] Paisley CE, Kay JN. Seeing stars: development and function of retinal astrocytes. Dev Biol. 2021;478:144–54. 34260962 10.1016/j.ydbio.2021.07.007 PMC8542354

[354] Vecino E, Rodriguez FD, Ruzafa N, Pereiro X, Sharma SC. Glia–neuron interactions in the mammalian retina. Prog Retin Eye Res. 2016;51:1–40. 26113209 10.1016/j.preteyeres.2015.06.003

[355] Nair CEM, Nickells RW. Chapter twenty neuroinflammation in glaucoma and optic nerve damage. Prog Mol Biol Transl. 2015;134:343–63. 10.1016/bs.pmbts.2015.06.010 26310164

[356] Chen H, Cho K-S, Vu THK, Shen C-H, Kaur M, Chen G, et al. Commensal microflora-induced T cell responses mediate progressive neurodegeneration in glaucoma. Nat Commun. 2018;9:3209. 30097565 10.1038/s41467-018-05681-9 PMC6086830

[357] Levraud J-P, Rawls JF, Clatworthy AE. Using zebrafish to understand reciprocal interactions between the nervous and immune systems and the microbial world. J Neuroinflammation. 2022;19:170. 35765004 10.1186/s12974-022-02506-x PMC9238045

[358] Jiang S, Kametani M, Chen DF. Adaptive immunity: new aspects of pathogenesis underlying neurodegeneration in glaucoma and optic neuropathy. Front Immunol. 2020;11:65. 32117239 10.3389/fimmu.2020.00065 PMC7031201

[359] West EL, Pearson RA, Tschernutter M, Sowden JC, MacLaren RE, Ali RR. Pharmacological disruption of the outer limiting membrane leads to increased retinal integration of transplanted photoreceptor precursors. Exp Eye Res. 2008;86:601–11. 18294631 10.1016/j.exer.2008.01.004 PMC2394572

[360] Kinouchi R, Takeda M, Yang L, Wilhelmsson U, Lundkvist A, Pekny M, et al. Robust neural integration from retinal transplants in mice deficient in GFAP and vimentin. Nat Neurosci. 2003;6:863–8. 12845328 10.1038/nn1088

[361] Fisher MJ, Loguidice M, Gutmann DH, Listernick R, Ferner RE, Ullrich NJ, et al. Gender as a disease modifier in neurofibromatosis type 1 optic pathway glioma. Ann Neurol. 2014;75:799–800. 24740685 10.1002/ana.24157

[362] Phay MH, Bauer SG, Baranov PY. Masking the “eat me” signal drastically increases the short-term survival of donor human retinal ganglion cells after xenotransplantation. Invest Ophthalmol Vis Sci. 2022;63:1117–1117.

[363] Demb JB, Singer JH. Functional circuitry of the retina. Ann Rev Vis Sci. 2015;1:263–89. 28532365 10.1146/annurev-vision-082114-035334 PMC5749398

[364] Ludwig AL, Mayerl SJ, Gao Y, Banghart M, Bacig C, Zepeda MAF, et al. Re-formation of synaptic connectivity in dissociated human stem cell-derived retinal organoid cultures. Proc National Acad Sci. 2023;120:e2213418120. 10.1073/pnas.2213418120 PMC9926218 36598946

[365] Bardy C, van den Hurk M, Eames T, Marchand C, Hernandez RV, Kellogg M, et al. Neuronal medium that supports basic synaptic functions and activity of human neurons in vitro. Proc National Acad Sci. 2015;112:E2725–34. 10.1073/pnas.1504393112 PMC4443325 25870293

[366] Matsuoka RL, Nguyen-Ba-Charvet KT, Parray A, Badea TC, Chédotal A, Kolodkin AL. Transmembrane semaphorin signalling controls laminar stratification in the mammalian retina. Nature. 2011;470:259–63. 21270798 10.1038/nature09675 PMC3063100

[367] Agostinone J, Alarcon-Martinez L, Gamlin C, Yu WQ, Wong ROL, Di_Polo A. Insulin signalling promotes dendrite and synapse regeneration and restores circuit function after axonal injury. Brain. 2018;141:1963–80. 10.1093/brain/awy142 PMC6022605 29931057

[368] Ray TA, Roy S, Kozlowski C, Wang J, Cafaro J, Hulbert SW, et al. Formation of retinal direction-selective circuitry initiated by starburst amacrine cell homotypic contact. Elife. 2018;7:e34241. 29611808 10.7554/eLife.34241 PMC5931800

[369] Vrabec F. “Displaced nerve cells” in the human retina. Graefe’s Arch Clin Exp Ophthalmol. 1986;224:143–6. 2419207 10.1007/BF02141487

[370] Curcio CA, Allen KA. Topography of ganglion cells in human retina. J Comp Neurol. 1990;300:5–25. 2229487 10.1002/cne.903000103

[371] Nadal-Nicolás FM, Salinas-Navarro M, Jiménez-López M, Sobrado-Calvo P, Villegas-Pérez MP, Vidal-Sanz M, et al. Displaced retinal ganglion cells in albino and pigmented rats. Front Neuroanat. 2014;8:99. 25339868 10.3389/fnana.2014.00099 PMC4186482

[372] Dumitrescu ON, Pucci FG, Wong KY, Berson DM. Ectopic retinal ON bipolar cell synapses in the OFF inner plexiform layer: Contacts with dopaminergic amacrine cells and melanopsin ganglion cells. J Comp Neurol. 2009;517:226–44. 19731338 10.1002/cne.22158 PMC3296562

[373] Chen M, Wang K, Lin B. Development and degeneration of cone bipolar cells are independent of cone photoreceptors in a mouse model of retinitis pigmentosa. PLoS One. 2012;7:e44036. 22952865 10.1371/journal.pone.0044036 PMC3432094

[374] Jacobi A, Tran NM, Yan W, Benhar I, Tian F, Schaffer R, et al. Overlapping transcriptional programs promote survival and axonal regeneration of injured retinal ganglion cells. Neuron. 2022;110:2625–2645.e7. 35767994 10.1016/j.neuron.2022.06.002 PMC9391321

[375] Li L, Fang F, Feng X, Zhuang P, Huang H, Liu P, et al. Single-cell transcriptome analysis of regenerating RGCs reveals potent glaucoma neural repair genes. Neuron. 2022;110:2646–2663.e6. 35952672 10.1016/j.neuron.2022.06.022 PMC9391304

[376] Tian F, Cheng Y, Zhou S, Wang Q, Monavarfeshani A, Gao K, et al. Core transcription programs controlling injury-induced neurodegeneration of retinal ganglion cells. Neuron. 2022;110:2607–2624.e8. 35767995 10.1016/j.neuron.2022.06.003 PMC9391318

[377] Varadarajan SG, Hunyara JL, Hamilton NR, Kolodkin AL, Huberman AD. Central nervous system regeneration. Cell. 2022;185:77–94. 34995518 10.1016/j.cell.2021.10.029 PMC10896592

[378] Pearson RA, Hippert C, Graca AB, Barber AC. Photoreceptor replacement therapy: challenges presented by the diseased recipient retinal environment. Visual Neurosci. 2014;31:333–44. 10.1017/S0952523814000200 24945529

[379] Riccomagno MM, Sun LO, Brady CM, Alexandropoulos K, Seo S, Kurokawa M, et al. Cas adaptor proteins organize the retinal ganglion cell layer downstream of integrin signaling. Neuron. 2014;81:779–86. 24559672 10.1016/j.neuron.2014.01.036 PMC3988023

[380] Moons L. Fueling axonal regeneration: dendritic energy to the rescue? Acta Ophthalmol. 2019;97. 10.1111/j.1755-3768.2019.8239.

[381] Santina LD, Yu AK, Harris SC, Soliño M, Ruiz TG, Most J, et al. Disassembly and rewiring of a mature converging excitatory circuit following injury. Cell Rep. 2021;36:109463. 34348156 10.1016/j.celrep.2021.109463 PMC8381591

[382] Hernandez M, Rodriguez FD, Sharma SC, Vecino E. Immunohistochemical changes in rat retinas at various time periods of elevated intraocular pressure. Mol Vis. 2008;15:2696–709. PMC2793899 20019879

[383] Lee H, Oh WC, Seong J, Kim J. Advanced fluorescence protein-based synapse-detectors. Front Synaptic Neurosci. 2016;8:16. 27445785 10.3389/fnsyn.2016.00016 PMC4927625

[384] Bleckert A, Schwartz GW, Turner MH, Rieke F, Wong ROL. Visual space is represented by nonmatching topographies of distinct mouse retinal ganglion cell types. Curr Biol. 2014;24:310–5. 24440397 10.1016/j.cub.2013.12.020 PMC3990865

[385] Bringmann A, Wiedemann P. Müller glial cells in retinal disease. Ophthalmologica. 2011;227:1–19. 21921569 10.1159/000328979

[386] Hippert C, Graca AB, Barber AC, West EL, Smith AJ, Ali RR, et al. Müller glia activation in response to inherited retinal degeneration is highly varied and disease-specific. PLoS One. 2015;10:e0120415. 25793273 10.1371/journal.pone.0120415 PMC4368159

[387] Bull ND, Limb GA, Martin KR. Human Müller Stem Cell (MIO-M1) transplantation in a rat model of glaucoma: survival, differentiation, and integration. Investig Opthalmol Vis Sci. 2008;49:3449. 10.1167/iovs.08-1770 18408183

[388] Singhal S, Lawrence JM, Bhatia B, Ellis JS, Kwan AS, MacNeil A, et al. Chondroitin sulfate proteoglycans and microglia prevent migration and integration of grafted müller stem cells into degenerating retina. Stem Cells. 2008;26:1074–82. 18218817 10.1634/stemcells.2007-0898

[389] Ma J, Chen M, Ai J, Young MJ, Ge J. Enhanced migration of engrafted retinal progenitor cells into the host retina via disruption of glial barriers. Molecular Vision. 2021;27:300–8. PMC8131176 34035644

[390] Suzuki T, Akimoto M, Imai H, Ueda Y, Mandai M, Yoshimura N, et al. Chondroitinase ABC treatment enhances synaptogenesis between transplant and host neurons in model of retinal degeneration. Cell Transplant. 2007;16:493–503. 17708339 10.3727/000000007783464966

[391] Singh RK, Occelli LM, Binette F, Petersen-Jones SM, Nasonkin IO. Transplantation of human embryonic stem cell-derived retinal tissue in the subretinal space of the cat eye. Stem Cells Dev. 2019;28:1151–66. 31210100 10.1089/scd.2019.0090 PMC6708274

[392] Ramirez AI, de Hoz R, Salobrar-Garcia E, Salazar JJ, Rojas B, Ajoy D, et al. The role of microglia in retinal neurodegeneration: Alzheimer’s disease, parkinson, and glaucoma. Front Aging Neurosci. 2017;9:214. 28729832 10.3389/fnagi.2017.00214 PMC5498525

[393] Rashid K, Akhtar-Schaefer I, Langmann T. Microglia in retinal degeneration. Front Immunol. 2019;10:1975. 31481963 10.3389/fimmu.2019.01975 PMC6710350

[394] Montilla A, Zabala A, Er-Lukowiak M, Rissiek B, Magnus T, Rodriguez-Iglesias N, et al. Microglia and meningeal macrophages depletion delays the onset of experimental autoimmune encephalomyelitis. Cell Death Dis. 2023;14:16. 36635255 10.1038/s41419-023-05551-3 PMC9835747

[395] Araque A, Navarrete M. Glial cells in neuronal network function. Philos Trans R Soc B Biol Sci. 2010;365:2375–81. 10.1098/rstb.2009.0313 PMC2894949 20603358

[396] Benfey N, Foubert D, Ruthazer ES. Glia regulate the development, function, and plasticity of the visual system from retina to cortex. Front Neural Circuit. 2022;16:826664. 10.3389/fncir.2022.826664 PMC8843846 35177968

[397] Rubino SJ, Mayo L, Wimmer I, Siedler V, Brunner F, Hametner S, et al. Acute microglia ablation induces neurodegeneration in the somatosensory system. Nat Commun. 2018;9:4578. 30385785 10.1038/s41467-018-05929-4 PMC6212411

[398] Roy S, Wang D, Rudzite AM, Perry B, Scalabrino ML, Thapa M, et al. Large-scale interrogation of retinal cell functions by 1-photon light-sheet microscopy. Cell Reports Methods. 2023;3:100453. 37159670 10.1016/j.crmeth.2023.100453 PMC10163030

[399] Khabou H, Orendorff E, Trapani F, Rucli M, Desrosiers M, Yger P, et al. Optogenetic Targeting of AII Amacrine Cells restores Retinal Computations performed by the Inner Retina. Biorxiv. 2022;2022.07.28.501925. 10.1016/j.omtm.2023.09.003 PMC10589896 37868206

[400] Rodgers J, Hughes S, Lindner M, Allen AE, Ebrahimi AS, Storchi R, et al. Functional integrity of visual coding following advanced photoreceptor degeneration. Curr Biol. 2023;33:474–486.e5. 36630957 10.1016/j.cub.2022.12.026 PMC7619324

[401] Gilhooley MJ, Lindner M, Palumaa T, Hughes S, Peirson SN, Hankins MW. A systematic comparison of optogenetic approaches to visual restoration. Mol Ther - Methods Clin Dev. 2022;25:111–23. 35402632 10.1016/j.omtm.2022.03.003 PMC8956963

[402] So K-F, Aguayo AJ. Lengthy regrowth of cut axons from ganglion cells after peripheral nerve transplantation into the retina of adult rats. Brain Res. 1985;328:349–54. 3986532 10.1016/0006-8993(85)91047-9

[403] Vidal-Sanz M, Bray G, Villegas-Perez M, Thanos S, Aguayo A. Axonal regeneration and synapse formation in the superior colliculus by retinal ganglion cells in the adult rat. J Neurosci. 1987;7:2894–909. 3625278 10.1523/JNEUROSCI.07-09-02894.1987 PMC6569122

[404] Vidal-Sanz M, Bray GM, Aguayo AJ. Regenerated synapses persist in the superior colliculus after the regrowth of retinal ganglion cell axons. J Neurocytol. 1991;20:940–52. 1809272 10.1007/BF01190471

[405] Laha B, Stafford BK, Huberman AD. Regenerating optic pathways from the eye to the brain. Science. 2017;356:1031–4. 28596336 10.1126/science.aal5060 PMC6333302

[406] Benowitz LI, He Z, Goldberg JL. Reaching the brain: advances in optic nerve regeneration. Exp Neurol. 2017;287:365–73. 26746987 10.1016/j.expneurol.2015.12.015

[407] Williams PR, Benowitz LI, Goldberg JL, He Z. Axon Regeneration in the Mammalian Optic Nerve. Annu Rev Vis Sci. 2020;6:195–213. 32936739 10.1146/annurev-vision-022720-094953

[408] Trakhtenberg EF, Li Y, Feng Q, Tso J, Rosenberg PA, Goldberg JL, et al. Zinc chelation and Klf9 knockdown cooperatively promote axon regeneration after optic nerve injury. Exp Neurol. 2018;300:22–9. 29106981 10.1016/j.expneurol.2017.10.025 PMC5745290

[409] Trakhtenberg EF, Wang Y, Morkin MI, Fernandez SG, Mlacker GM, Shechter JM, et al. Regulating Set-β’s Subcellular Localization Toggles Its Function between Inhibiting and Promoting Axon Growth and Regeneration. J Neurosci. 2014;34:7361–74. 24849368 10.1523/JNEUROSCI.3658-13.2014 PMC4028506

[410] Lukomska A, Theune WC, Xing J, Frost MP, Damania A, Gupta M, et al. Experimental gene expression of developmentally downregulated Crmp1, Crmp4, and Crmp5 promotes axon regeneration and retinal ganglion cell survival after optic nerve injury. Brain Res. 2023;1809:148368. 37059258 10.1016/j.brainres.2023.148368 PMC10227878

[411] Lukomska A, Kim J, Rheaume BA, Xing J, Hoyt A, Lecky E, et al. Developmentally upregulated transcriptional elongation factor a like 3 suppresses axon regeneration after optic nerve injury. Neurosci Lett. 2021;765:136260. 34560191 10.1016/j.neulet.2021.136260 PMC8572158

[412] Huebner EA, Strittmatter SM. Cell biology of the axon. Results Problems Cell Differ. 2009;48:305–60. 10.1007/400_2009_19 PMC2846285 19582408

[413] Bonilla IE, Tanabe K, Strittmatter SM. Small proline-rich repeat protein 1A is expressed by axotomized neurons and promotes axonal outgrowth. J Neurosci. 2002;22:1303–15. 11850458 10.1523/JNEUROSCI.22-04-01303.2002 PMC6757578

[414] Wong KA, Benowitz LI. Retinal ganglion cell survival and axon regeneration after optic nerve injury: role of inflammation and other factors. Int J Mol Sci. 2022;23:10179. 36077577 10.3390/ijms231710179 PMC9456227

[415] Moore DL, Blackmore MG, Hu Y, Kaestner KH, Bixby JL, Lemmon VP, et al. KLF family members regulate intrinsic axon regeneration ability. Science. 2009;326:298–301. 19815778 10.1126/science.1175737 PMC2882032

[416] Apara A, Galvao J, Wang Y, Blackmore M, Trillo A, Iwao K, et al. KLF9 and JNK3 interact to suppress axon regeneration in the adult CNS. J Neurosci. 2017;37:9632–44. 28871032 10.1523/JNEUROSCI.0643-16.2017 PMC5628408

[417] Koh S, Roy S, Eroglu O, Strader S, Chen WJ, Kay JN, et al. Thrombospondin-1 Promotes Circuit-Specific Synapse Formation via β1-Integrin. Biorxiv. 2019;866590.

[418] Bray ER, Yungher BJ, Levay K, Ribeiro M, Dvoryanchikov G, Ayupe AC, et al. Thrombospondin-1 Mediates Axon Regeneration in Retinal Ganglion Cells. Neuron. 2019;103:642–657.e7. 31255486 10.1016/j.neuron.2019.05.044 PMC6706310

[419] Yin Y, Henzl MT, Lorber B, Nakazawa T, Thomas TT, Jiang F, et al. Oncomodulin is a macrophage-derived signal for axon regeneration in retinal ganglion cells. Nat Neurosci. 2006;9:843–52. 16699509 10.1038/nn1701

[420] Vance JE, Campenot RB, Vance DE. The synthesis and transport of lipids for axonal growth and nerve regeneration. Biochimica Et Biophysica Acta Bba - Mol Cell Biology Lipids. 2000;1486:84–96. 10.1016/s1388-1981(00)00050-0 10856715

[421] Yang C, Wang X, Wang J, Wang X, Chen W, Lu N, et al. Rewiring neuronal glycerolipid metabolism determines the extent of axon regeneration. Neuron. 2020;105:276–292.e5. 31786011 10.1016/j.neuron.2019.10.009 PMC6975164

[422] Schuster CM, Davis GW, Fetter RD, Goodman CS. Genetic Dissection of Structural and Functional Components of Synaptic Plasticity. I. Fasciclin II Controls Synaptic Stabilization and Growth. Neuron. 1996;17:641–54. 10.1016/s0896-6273(00)80197-x 8893022

[423] Erskine L, Herrera E. Connecting the retina to the brain. ASN Neuro. 2014;6:1759091414562107. 25504540 10.1177/1759091414562107 PMC4720220

[424] Murcia-Belmonte V, Erskine L. Wiring the binocular visual pathways. Int J Mol Sci. 2019;20:3282. 31277365 10.3390/ijms20133282 PMC6651880

[425] Subramani M, Hook MV, Rajamoorthy M, Qiu F, Ahmad I. Human Retinal Ganglion Cells Respond to Evolutionarily Conserved Chemotropic Cues for Intra Retinal Guidance and Regeneration. Biorxiv. 2023;2023.02.01.526677. 10.1093/stmcls/sxad061 PMC13032126 37591511

[426] Harada C, Kimura A, Guo X, Namekata K, Harada T. Recent advances in genetically modified animal models of glaucoma and their roles in drug repositioning. Br J Ophthalmol. 2019;103:161–6. 30366949 10.1136/bjophthalmol-2018-312724 PMC6362806

[427] Patel M, Ahn S, Koh WG. Topographical pattern for neuronal tissue engineering. J Ind Eng Chem. 2022;114:19–32.

[428] Hoffman-Kim D, Mitchel JA, Bellamkonda RV. Topography, cell response, and nerve regeneration. Annu Rev Biomed Eng. 2010;12:203–31. 20438370 10.1146/annurev-bioeng-070909-105351 PMC3016849

[429] Kuwajima T, Yoshida Y, Takegahara N, Petros TJ, Kumanogoh A, Jessell TM, et al. Optic Chiasm Presentation of Semaphorin6D in the Context of Plexin-A1 and Nr-CAM Promotes Retinal Axon Midline Crossing. Neuron. 2012;74:676–90. 22632726 10.1016/j.neuron.2012.03.025 PMC3361695

[430] Conceição R, Evans RS, Pearson CS, Hänzi B, Osborne A, Deshpande SS, et al. Expression of developmentally important axon guidance cues in the adult optic chiasm. Invest Ophth Vis Sci. 2019;60:4727–39. 10.1167/iovs.19-26732 PMC6859889 31731293

[431] Knickmeyer MD, Mateo JL, Heermann S. BMP signaling interferes with optic chiasm formation and retinal ganglion cell pathfinding in zebrafish. Int J Mol Sci. 2021;22:4560. 33925390 10.3390/ijms22094560 PMC8123821

[432] Erskine L, Herrera E. The retinal ganglion cell axon’s journey: Insights into molecular mechanisms of axon guidance. Dev Biol. 2007;308:1–14. 17560562 10.1016/j.ydbio.2007.05.013

[433] Mason C, Slavi N. Retinal ganglion cell axon wiring establishing the binocular circuit. Annu Rev Vis Sci. 2020;6:215–36. 32396770 10.1146/annurev-vision-091517-034306

[434] Lim JHA, Stafford BK, Nguyen PL, Lien BV, Wang C, Zukor K, et al. Neural activity promotes long-distance, target-specific regeneration of adult retinal axons. Nat Neurosci. 2016;19:1073–84. 27399843 10.1038/nn.4340 PMC5708130

[435] Varadarajan SG, Huberman AD. Assembly and repair of eye-to-brain connections. Curr Opin Neurobiol. 2018;53:198–209. 30339988 10.1016/j.conb.2018.10.001 PMC6504177

[436] Li S, Yang C, Zhang L, Gao X, Wang X, Liu W, et al. Promoting axon regeneration in the adult CNS by modulation of the melanopsin/GPCR signaling. Proc Natl Acad Sci. 2016;113:1937–42. 26831088 10.1073/pnas.1523645113 PMC4763730

[437] Graham DM, Wong KY. Melanopsin-expressing, Intrinsically Photosensitive Retinal Ganglion Cells (ipRGCs). Webvision: The Organization of the Retina and Visual System. 2016.

[438] Li Y, He X, Kawaguchi R, Zhang Y, Wang Q, Monavarfeshani A, et al. Microglia-organized scar-free spinal cord repair in neonatal mice. Nature. 2020;587:613–8. 33029008 10.1038/s41586-020-2795-6 PMC7704837

[439] Fu H, Zhao Y, Hu D, Wang S, Yu T, Zhang L. Depletion of microglia exacerbates injury and impairs function recovery after spinal cord injury in mice. Cell Death Dis. 2020;11:528. 32661227 10.1038/s41419-020-2733-4 PMC7359318

[440] Reichenbach A, Bringmann A. Glia of the human retina. Glia. 2020;68:768–96. 31793693 10.1002/glia.23727

[441] García M, Forster V, Hicks D, Vecino E. Effects of müller glia on cell survival and neuritogenesis in adult porcine retina in vitro. Investig Ophthalmol Vis Sci. 2002;43:3735–43. 12454045

[442] Kugler EC, Greenwood J, MacDonald RB. The “Neuro-Glial-Vascular” Unit: The Role of Glia in Neurovascular Unit Formation and Dysfunction. Front Cell Dev Biol. 2021;9:732820. 34646826 10.3389/fcell.2021.732820 PMC8502923

[443] Toy D, Namgung U. Role of glial cells in axonal regeneration. Exp Neurobiology. 2013;22:68–76. 10.5607/en.2013.22.2.68 PMC3699676 23833555

[444] Yuan Y, Wang Y, Wu S, Zhao MY. Review: Myelin clearance is critical for regeneration after peripheral nerve injury. Front Neurol. 2022;13:908148. 36588879 10.3389/fneur.2022.908148 PMC9801717

[445] Provis JM, Diaz CM, Penfold PL. Microglia in human retina: a heterogeneous population with distinct ontogenies. Perspect Dev Neurobi. 1996;3:213–22. 8931095

[446] Li F, Sami A, Noristani HN, Slattery K, Qiu J, Groves T, et al. Glial metabolic rewiring promotes axon regeneration and functional recovery in the central nervous system. Cell Metab. 2020;32:767–785.e7. 32941799 10.1016/j.cmet.2020.08.015 PMC7642184

[447] Xing J, Lukomska A, Rheaume BA, Kim J, Sajid MS, Damania A, et al. Post-injury born oligodendrocytes incorporate into the glial scar and contribute to the inhibition of axon regeneration. Development. 2023;150:201311. 10.1242/dev.201311 PMC10163352 36971369

[448] Fague L, Liu YA, Marsh-Armstrong N. The basic science of optic nerve regeneration. Ann Transl Med. 2021;9:1276. 10.21037/atm-20-5351 PMC8421956 34532413

[449] Stevenson R, Samokhina E, Rossetti I, Morley JW, Buskila Y. Neuromodulation of glial function during neurodegeneration. Front Cell Neurosci. 2020;14:278. 32973460 10.3389/fncel.2020.00278 PMC7473408

[450] Patani R, Hardingham GE, Liddelow SA. Functional roles of reactive astrocytes in neuroinflammation and neurodegeneration. Nat Rev Neurol. 2023;19:395–409. 10.1038/s41582-023-00822-1 37308616

[451] Gallego BI, Salazar JJ, de Hoz R, Rojas B, Ramírez AI, Salinas-Navarro M, et al. IOP induces upregulation of GFAP and MHC-II and microglia reactivity in mice retina contralateral to experimental glaucoma. J Neuroinflammation. 2012;9:92. 22583833 10.1186/1742-2094-9-92 PMC3410794

[452] Rojas B, Gallego BI, Ramírez AI, Salazar JJ, de Hoz R, Valiente-Soriano FJ, et al. Microglia in mouse retina contralateral to experimental glaucoma exhibit multiple signs of activation in all retinal layers. J Neuroinflammation. 2014;11:133. 25064005 10.1186/1742-2094-11-133 PMC4128533

[453] Zhou M, Bear J, Roberts PA, Janiak FK, Semmelhack J, Yoshimatsu T, et al. Zebrafish retinal ganglion cells asymmetrically encode spectral and temporal information across visual space. Curr Biol. 2020;30:2927–2942.e7. 32531283 10.1016/j.cub.2020.05.055 PMC7416113

[454] Gestri G, Link BA, Neuhauss SCF. The visual system of zebrafish and its use to model human ocular diseases. Dev Neurobiol. 2012;72:302–27. 21595048 10.1002/dneu.20919 PMC3202066

[455] Sharf T, Kalakuntla T, Lee DJ, Gokoffski KK. Electrical devices for visual restoration. Surv Ophthalmol. 2022;67:793–800. 34487742 10.1016/j.survophthal.2021.08.008 PMC9241872

[456] Gokoffski KK, Jia X, Shvarts D, Xia G, Zhao M. Physiologic electrical fields direct retinal ganglion cell axon growth in vitro. Invest Ophth Vis Sci. 2019;60:3659–68. 10.1167/iovs.18-25118 PMC6716951 31469406

[457] Paknahad J, Machnoor M, Lazzi G, Gokoffski KK. The influence of electrode properties on induced voltage gradient along the rat optic nerve. Ieee J Electromagn Rf Microwaves Medicine Biology. 2022;6:321–30. 10.1109/jerm.2022.3165171 PMC10004096 36910030

[458] Varadarajan SG, Wang F, Dhande OS, Le P, Duan X, Huberman AD. Postsynaptic neuronal activity promotes regeneration of retinal axons. Cell Rep. 2023;42:112476. 37141093 10.1016/j.celrep.2023.112476 PMC10247459

[459] Noble M, Tseng KC (Chris), Li H, Elfar JC. 4-Aminopyridine as a Single Agent Diagnostic and Treatment for Severe Nerve Crush Injury. Mil Med. 2019;184:379–85. 10.1093/milmed/usy399 PMC6433095 30901424

[460] Tseng K, Li H, Clark A, Sundem L, Zuscik M, Noble M, et al. 4-Aminopyridine promotes functional recovery and remyelination in acute peripheral nerve injury. Embo Mol Med. 2016;8:1409–20. 27861125 10.15252/emmm.201506035 PMC5167128

[461] Sabel BA. Restoration of vision I: neurobiological mechanisms of restoration and plasticity after brain damage - a review. Restor Neurol Neuros. 1999;15:177–200. 12671232

[462] Ito Y, Shimazawa M, Hara H. Review: an approach for neuroprotective therapies of secondary brain damage after excitotoxic retinal injury in mice. Cns Neurosci Ther. 2010;16:e169–79. 20553302 10.1111/j.1755-5949.2010.00176.x PMC6493845

[463] Joly O, Frankó E. Neuroimaging of amblyopia and binocular vision: a review. Frontiers Integr Neurosci. 2014;8:62. 10.3389/fnint.2014.00062 PMC4123726 25147511

[464] You Y, Gupta VK, Graham SL, Klistorner A. Anterograde degeneration along the visual pathway after optic nerve injury. PLoS One. 2012;7:e52061. 23300590 10.1371/journal.pone.0052061 PMC3530579

[465] Tanaka H, Ito Y, Nakamura S, Shimazawa M, Hara H. Involvement of brain-derived neurotrophic factor in time-dependent neurodegeneration in the murine superior colliculus after intravitreal injection of N-methyl-D-aspartate. Mol Vis. 2008;15:662–9. PMC2664844 19347051

[466] Duncan RO, Sample PA, Weinreb RN, Bowd C, Zangwill LM. Retinotopic organization of primary visual cortex in glaucoma: Comparing fMRI measurements of cortical function with visual field loss. Prog Retin Eye Res. 2007;26:38–56. 17126063 10.1016/j.preteyeres.2006.10.001 PMC1940234

[467] Wang J, Li T, Sabel BA, Chen Z, Wen H, Li J, et al. Structural brain alterations in primary open angle glaucoma: a 3T MRI study. Sci Rep. 2016;6:18969. 26743811 10.1038/srep18969 PMC4705520

[468] Chapman GB, Tarboush R, Connaughton VP. The effects of rearing light level and duration differences on the optic nerve, brain, and associated structures in developing zebrafish larvae: a light and transmission electron microscope study. Anat Rec: Adv Integr Anat Evol Biol. 2012;295:515–31. 10.1002/ar.22403 22252993

[469] de Lima S, Koriyama Y, Kurimoto T, Oliveira JT, Yin Y, Li Y, et al. Full-length axon regeneration in the adult mouse optic nerve and partial recovery of simple visual behaviors. Proc National Acad Sci. 2012;109:9149–54. 10.1073/pnas.1119449109 PMC3384191 22615390

[470] Hong G, Yang X, Zhou T, Lieber CM. Mesh electronics: a new paradigm for tissue-like brain probes. Curr Opin Neurobiol. 2018;50:33–41. 29202327 10.1016/j.conb.2017.11.007 PMC5984112

[471] Zhou T, Hong G, Fu T-M, Yang X, Schuhmann TG, Viveros RD, et al. Syringe-injectable mesh electronics integrate seamlessly with minimal chronic immune response in the brain. Proc Natl Acad Sci. 2017;114:5894–9. 28533392 10.1073/pnas.1705509114 PMC5468665

[472] Ross JW, de Castro JPF, Zhao J, Samuel M, Walters E, Rios C, et al. Generation of an inbred miniature pig model of retinitis pigmentosa. Investig Opthalmol Vis Sci. 2012;53:501. 10.1167/iovs.11-8784 PMC3292381 22247487

[473] Shrader SM, Greentree WF. Göttingen minipigs in ocular research. Toxicol Pathol. 2018;46:403–7. 29683084 10.1177/0192623318770379

[474] Weber AJ, Harman CD. BDNF treatment and extended recovery from optic nerve trauma in the cat. Investig Opthalmol Vis Sci. 2013;54:6594. 10.1167/iovs.13-12683 PMC3803139 23989190

[475] Diekmann H, Kalbhen P, Fischer D. Characterization of optic nerve regeneration using transgenic zebrafish. Front Cell Neurosci. 2015;9:118. 25914619 10.3389/fncel.2015.00118 PMC4391235

[476] Williams DL. Regenerating reptile retinas: a comparative approach to restoring retinal ganglion cell function. Eye. 2017;31:167–72. 27834958 10.1038/eye.2016.224 PMC5306453

[477] Vergara MN, Rio-Tsonis KD. Retinal regeneration in the Xenopus laevis tadpole: a new model system. Mol Vis. 2008;15:1000–13. PMC2684558 19461929

[478] Belrose JL, Prasad A, Sammons MA, Gibbs KM, Szaro BG. Comparative gene expression profiling between optic nerve and spinal cord injury in Xenopus laevis reveals a core set of genes inherent in successful regeneration of vertebrate central nervous system axons. BMC Genomics. 2020;21:540. 32758133 10.1186/s12864-020-06954-8 PMC7430912

[479] Beckers A, Masin L, Dyck AV, Bergmans S, Vanhunsel S, Zhang A, et al. Optic nerve injury-induced regeneration in the adult zebrafish is accompanied by spatiotemporal changes in mitochondrial dynamics. Neural Regen Res. 2022;18:219–25. 10.4103/1673-5374.344837 PMC9241429 35799546

[480] Whitworth GB, Misaghi BC, Rosenthal DM, Mills EA, Heinen DJ, Watson AH, et al. Translational profiling of retinal ganglion cell optic nerve regeneration in Xenopus laevis. Dev Biol. 2017;426:360–73. 27471010 10.1016/j.ydbio.2016.06.003 PMC5897040

[481] Templeton JP, Geisert EE. A practical approach to optic nerve crush in the mouse. Mol Vis. 2011;18:2147–52. PMC3413441 22876142

[482] Fischer D, Harvey AR, Pernet V, Lemmon VP, Park KK. Optic nerve regeneration in mammals: regenerated or spared axons? Exp Neurol. 2017;296:83–8. 28716559 10.1016/j.expneurol.2017.07.008 PMC5564230

[483] Sun W, Chao G, Shang M, Wu Q, Xia Y, Wei Q, et al. Optic nerve injury models under varying forces. Int Ophthalmol. 2023;43:757–69. 10.1007/s10792-022-02476-2 PMC10042766 36038691

[484] Cameron E, Xia X, Galvao J, Ashouri M, Kapiloff M, Goldberg J. Optic Nerve Crush in Mice to Study Retinal Ganglion Cell Survival and Regeneration. Bio-protocol. 2020;10:e3559. 10.21769/BioProtoc.3559 PMC7197875 32368566

[485] Yao F, Zhang E, Gao Z, Ji H, Marmouri M, Xia X. Did you choose appropriate tracer for retrograde tracing of retinal ganglion cells? The differences between cholera toxin subunit B and Fluorogold. PLoS ONE. 2018;13:e0205133. 30289890 10.1371/journal.pone.0205133 PMC6173421

[486] Yin Y, Benowitz LI. Glaucoma, methods and protocols. Methods Mol Biol. 2017;1695:187–205. 10.1007/978-1-4939-7407-8_16 29190028

[487] Zor F, Polat M, Kulahci Y, Sahin H, Aral AM, Erbas VE, et al. Demonstration of technical feasibility and viability of whole eye transplantation in a rodent model. J Plastic Reconstr Aesthetic Surg. 2019;72:1640–50. 10.1016/j.bjps.2019.05.042 31377202

[488] Bourne D, Li Y, Komatsu C, Miller MR, Davidson EH, He L, et al. Whole-eye transplantation: a look into the past and vision for the future. Eye. 2017;31:179–84. 27983731 10.1038/eye.2016.272 PMC5306462

[489] Gramlich OW, Joachim SC, Gottschling PF, Laspas P, Cuny CS, Pfeiffer N, et al. Ophthalmopathology in rats with MBP-induced experimental autoimmune encephalomyelitis. Graefe’s Arch Clin Exp Ophthalmol. 2011;249:1009–20. 21344308 10.1007/s00417-011-1633-9

[490] Bernstein SL, Johnson MA, Miller NR. Nonarteritic anterior ischemic optic neuropathy (NAION) and its experimental models. Prog Retin Eye Res. 2011;30:167–87. 21376134 10.1016/j.preteyeres.2011.02.003 PMC3367439

[491] Grinsell D, Keating CP. Peripheral nerve reconstruction after injury: a review of clinical and experimental therapies. Biomed Res Int. 2014;2014:1–13. 10.1155/2014/698256 PMC4167952 25276813

